# Double Rotors with Fluxional Axles: Domino Rotation and Azide–Alkyne Huisgen Cycloaddition Catalysis

**DOI:** 10.1002/anie.202002739

**Published:** 2020-06-10

**Authors:** Abir Goswami, Michael Schmittel

**Affiliations:** ^1^ Center of Micro and Nanochemistry and Engineering, Organische Chemie I University of Siegen Adolf-Reichwein Str. 2 57068 Siegen Germany

**Keywords:** catalysis, multicomponent assembly, rotational exchange, self-sorting, supramolecular machines

## Abstract

The simple preparation of the multicomponent devices [Cu_4_(**A**)_2_]^4+^ and [Cu_2_(**A**)(**B**)]^2+^, both rotors with fluxional axles undergoing domino rotation, highlights the potential of self‐sorting. The concept of domino rotation requires the interconversion of axle and rotator, allowing the spatiotemporal decoupling of two degenerate exchange processes in [Cu_4_(**A**)_2_]^4+^ occurring at 142 kHz. Addition of two equiv of **B** to rotor [Cu_4_(**A**)_2_]^4+^ afforded the heteromeric two‐axle rotor [Cu_2_(**A**)(**B**)]^2+^ with two distinct exchange processes (64.0 kHz and 0.55 Hz). The motion requiring a pyridine→zinc porphyrin bond cleavage is 1.2×10^5^ times faster than that operating via a terpyridine→[Cu(phenAr_2_)]^+^ rupture. Finally, both rotors are catalysts due to their copper(I) content. The fast domino rotor (142 kHz) was shown to suppress product inhibition in the catalysis of the azide–alkyne Huisgen cycloaddition.

## Graphical abstract

**Well sorted**: The simple preparation of the homodimeric rotor [Cu_4_(**A**)_2_]^4+^ and the heteromeric two‐axle rotor [Cu_2_(**A**)(**B**)]^2+^ highlights the potential of self‐sorting. Here, both rotors have fluxional axles undergoing domino rotation. The fast domino rotor [Cu_4_(**A**)_2_]^4+^ (142 kHz) suppresses product inhibition in the catalysis of the azide–alkyne Huisgen cycloaddition.

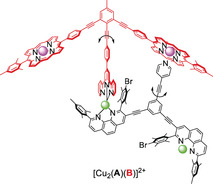

Dynamic functional devices composed of multiple molecular components are attracting ever‐increasing interest for two major reasons.^1^, ^2^, ^3^ Firstly, the impressive properties of multicomponent machines have been amply demonstrated by nature,^4^ and secondly, the facile exchange of components in such systems opens the door for self‐repair during operation or even evolution toward novel emerging properties.

Correlated motion and transmission of movement have inspired the creativity of chemists and have led to exciting devices, such as molecular motors^5^ and gears,^6^ ball bearings,^2^ molecular muscles,^7^ rotaxanes,^8^ molecular turnstiles,^9^ caterpillar tracks^10^ and rotary transduction modules.^11^ In all cases, though, the correlated motions happen simultaneously.

While fluxional molecules involving bond cleavage/formation have a long‐standing history,^12^ related dynamics within multiple degenerate structures in supramolecular multicomponent devices is scarce.^13^, ^14^ Herein, for the first time, multicomponent rotors^15^, ^16^, ^17^, ^18^, ^19^ are equipped with fluxional axles allowing them to undergo domino^20^ rotation. Such domino rotors are characterized by a unique feature: the rotator arm of the first rotor subunit intra(supra)molecularly interconverts into the axle of the second rotor subunit. The homodimeric double rotor [Cu_4_(**A**)_2_]^4+^ is held together by two pyridine (py)→copper(I) phenanthroline interactions (=HETPYP binding:^21^ Heteroleptic Pyridine and Phenanthroline complexation). Two further copper(I) phenanthroline sites without additional ligands serve as recipient stations for the rotator arm (Scheme 1). By design, only one of the pyridine arms of **A** serves as a rotational axis at a given time. In the homodimeric rotor the domino rotations occur via isoenergetic transition states.

**Scheme 1:**
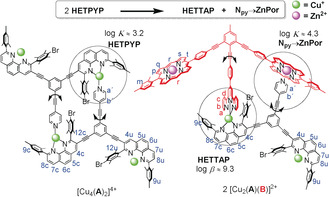
Two two‐axle double rotors. Log*K* denotes the binding constant of the single‐step pyridine association and log*β* describes the overall stability.

In order to generate two diverse domino rotational exchange processes with distinct activation barriers, we constructed the heteromeric double rotor [Cu_2_(**A**)(**B**)]^2+^ using two different binding motifs, a HETTAP (=Heteroleptic Terpyridine and Phenanthroline complexation)^22^ and a pyridine→zinc porphyrin (*N*_py_→ZnPor)^10^ coordination. Finally, in both rotors the effect of rotational motion on suppression of product inhibition was probed in a copper(I)‐catalyzed^23^, ^24^ azide–alkyne Huisgen^25^ cycloaddition. Although both rotors have the same kind of copper(I) centers, their catalytic activity is different due to dissimilar rotational rates.

The design of ligands **A** and **B** was guided by the geometric fit at the coordination sites in both aggregates [Cu_4_(**A**)_2_]^4+^ and [Cu_2_(**A**)(**B**)]^2+^. With ligand **B** being known from former work,^26^ we had only to synthesize ligand **A**, in which two shielded phenanthrolines and one pyridyl unit are connected to the 1,3,5‐positions of a benzene core. The geometry of **A** should lead to a dimeric parallelogram‐type structure in [Cu_4_(**A**)_2_]^4+^ with two antiparallel pyridyl units operating as axles. After mixing two equiv of copper(I) and one equiv of **A**, the two‐component dimer [Cu_4_(**A**)_2_]^4+^ was furnished quantitatively, as evidenced by spectroscopic data (^1^H NMR, ^1^H–^1^H COSY, UV/Vis, ESI‐MS). The single peak in the ESI mass spectrum at *m*/*z*=684.8 with correct isotopic distribution (SI, Figure S37), the single set of signals in the ^1^H‐DOSY {*D*=4.1×10^−10^ m^2^ s^−1^, *r*≈12.9 Å, (Supporting Information, Figure S21)}, and the correct elemental analysis confirmed quantitative formation of [Cu_4_(**A**)_2_]^4+^.

The single set of protons (4‐H, 5‐H, 6‐H, 7‐H, 8‐H) for all four phenanthrolines unmistakably suggested rapid exchange of the pyridine arms across all four copper(I) sites, requiring fast *N*_py_→[Cu(phenAr_2_)]^+^ (=HETPYP) bond cleavage. At low temperature (−50 °C) the phenanthroline signals in the ^1^H NMR spectrum (Figure 1 A) separated into two sets (1:1). Kinetic analysis provided the exchange frequency (*k*) at different temperatures with *k*_298_=142 kHz (at 298 K). The activation data were determined as Δ*H* ^≠^=51.1±0.7 kJ mol^−1^, Δ*S* ^≠^=25.5±3.0 J mol^−1^ K^−1^, and Δ*G* ^≠^_298_=43.5 kJ mol^−1^ (Supporting Information, Figure S24). Since the kinetic data for exchange in [Cu_4_(**A**)_2_]^4+^ are very similar to those of previously reported nanorotors that operate via single *N*_py_→[Cu(phenAr_2_)]^+^ dissociation (Δ*G* ^≠^_298_=46.6 kJ mol^−1^),1b, 16, 27, 32 there is convincing evidence that only one HETPYP interaction is cleaved at any given time in [Cu_4_(**A**)_2_]^4+^. Hence, the mechanism of exchange seems to follow an intramolecular nondirectional rotation involving two axles with dissociation of one *N*_py_→[Cu(phenAr_2_)]^+^ interaction being the rate‐limiting step. Due to symmetry, both rotational axles in the homodimeric rotor [Cu_4_(**A**)_2_]^4+^ have equal probability to dissociate.

**Figure 1:**
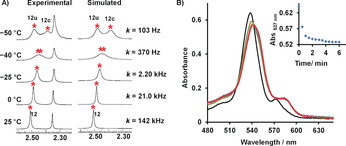
A) Partial variable‐temperature (VT) ^1^H NMR spectra of homodimeric rotor [Cu_4_(**A**)_2_]^4+^ in CD_2_Cl_2_. Experimental and theoretical splitting of proton signal 12‐H furnished rate data for rotation at different temperatures. B) UV/Vis spectral changes with time after addition of 0.5 equiv of [Cu_4_(**A**)_2_]^4+^ to **B** (1.2×10^−5^ m). Inset: Change of absorbance at *λ*=537 nm with time. Half‐life *t*_1/2_=30 s.

For the heteromeric rotor, we have chosen ligands **A** and **B** held together by two orthogonal dynamic interactions,^26^ that is, the weak *N*_py_→ZnPor interaction (Scheme 2; [(**2**)⋅(**4**)], log*K*_**2**→**4**_=4.3) and the robust HETTAP linkage (for [Cu(**1**)(**3**)]^+^, log *β*=9.3).^19^ Ligand **B** was designed in a way that the pyridine terminus of ligand **A** should bind to the ZnPor of **B**, while the tridentate terpyridine (tpy) ligand of **B** is connected with the copper(I) phenanthroline unit of **A**. When the HETTAP complex is intact it will serve as rotational axle, leading to an exchange of the pyridine terminus of **A** between both ZnPor sites of **B**. Similarly, when the *N*_py_→ZnPor interaction serves as rotational axle, both copper(I)‐loaded phenanthrolines will exchange their position by HETTAP dissociation/association. The *N*_py_→ZnPor bond cleavage should have a rather low energy of activation,^26^ whereas the HETTAP binding is known for its slow dynamics as demonstrated by Sauvage using interlocked molecular machines.7a, 28

**Scheme 2:**
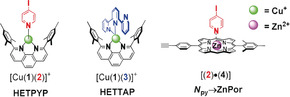
Heteroleptic binding motifs used to set up fluxional axles.

The heteromeric rotor [Cu_2_(**A**)(**B**)]^2+^ may be prepared directly from all reactants or via reshuffling of components by adding 2.0 equiv of **B** to [Cu_4_(**A**)_2_]^4+^ in *d*_2_‐dichloromethane at 25 °C (Figure 2). A time‐dependent UV/Vis spectroscopic study confirmed that conversion of the homodimeric [Cu_4_(**A**)_2_]^4+^ to the heteromeric [Cu_2_(**A**)(**B**)]^2+^ took 6 min at room temperature (Figure 1 B). The overall equilibrium constant for this transformation was determined as log *β*=5.98 per reacted [Cu_4_(**A**)_2_]^4+^ from a UV/Vis titration of [Cu_4_(**A**)_2_]^4+^ to **B** using a multivariate spectrophotometric analysis (Supporting Information, Figure S41). Formation of [Cu_2_(**A**)(**B**)]^2+^ was ascertained by spectroscopic data (^1^H NMR, ^1^H–^1^H COSY, UV/Vis, ESI‐MS) and elemental analysis. For instance, the ESI mass spectrum of the solution showed a single peak at *m*/*z*=1486.1 with the expected isotopic distribution (Supporting Information, Figure S39). In the ^1^H NMR spectrum, the chemical shift of the terpyridine protons (a‐H, b‐H, c‐H, d‐H, e‐H) of **B** indicated the presence of a HETTAP complexation site. The upfield shift of the pyridine protons a′‐H and b′‐H of ligand **A** from 6.93 and 7.23 ppm to 2.20 and 5.44 ppm, respectively, verified the *N*_py_→ZnPor binding. This coordination motif was further validated by a redshift of the ZnPor's Q‐band from 537 to 544 nm in the UV/Vis spectrum (Supporting Information, Figure S43). Finally, DOSY and elemental analysis confirmed quantitative formation of [Cu_2_(**A**)(**B**)]^2+^ (Supporting Information, Figure S22).

**Figure 2:**
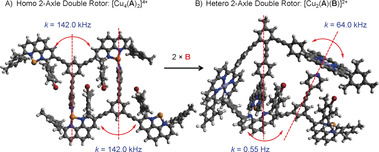
PM6‐optimized structures of [Cu_2_(**A**)(**B**)]^2+^ and [Cu_4_(**A**)_2_]^4+^. The transformation of rotors is exergonic: 2×HETPYP→HETTAP+*N*_py_→ZnPor. For stability constants (log*β*/K) see Figure 1 and ref. 19.

Rapid exchange of the pyridyl head of **A** between both ZnPor sites of **B** in [Cu_2_(**A**)(**B**)]^2+^ was indicted by a single set of ZnPor signals in the ^1^H NMR spectrum. In contrast, two individual signal sets for both phenanthrolines suggested that exchange at the HETTAP sites was slow on the NMR timescale (at 25 °C).

In order to measure the fast rotational dynamics in rotor [Cu_2_(**A**)(**B**)]^2+^ we analyzed the ^1^H NMR signal of the porphyrin protons r‐H. While it showed up as a sharp singlet (*δ*=10.23 ppm) at room temperature (Figure 3 A), it diagnostically separated into two singlets (1:1) at *δ*=10.22 and 10.19 ppm at −75 °C. The signal at *δ*=10.19 ppm was assigned to the pyridine‐coordinated ZnPor, whereas the conformationally unrestricted second zinc porphyrin furnished a signal at *δ*=10.22 ppm. A kinetic analysis provided the exchange frequency (*k*) as *k*_298_=64.0×10^3^ s^−1^ at 298 K (Supporting Information, Figure S23). Using the kinetic data over the whole temperature range furnished the activation parameters as Δ*H* ^≠^=46.9±0.4 kJ mol^−1^ and Δ*S* ^≠^=4.8±1.2 J mol^−1^ K^−1^ and the activation free energy for spinning at 298 K as Δ*G* ^≠^_298_=45.5 kJ mol^−1^ (Supporting Information, Figure S23).

**Figure 3:**
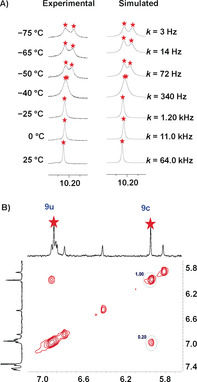
A) ^1^H VT‐NMR spectra of heteromeric rotor [Cu_2_(**A**)(**B**)]^2+^ in CD_2_Cl_2_/CD_3_CN (5:1). Experimental and theoretical splitting of the signal of proton r‐H furnished rate data at different temperatures. B) Partial ^1^H–^1^H ROESY NMR spectra (400 MHz, 298 K) of [Cu_2_(**A**)(**B**)]^2+^ in CD_2_Cl_2_/CD_3_CN (5:1).

The kinetics of the slow exchange requiring cleavage at the HETTAP site was evaluated by a ^1^H–^1^H ROESY (Figure 3 B) experiment in CD_2_Cl_2_/CD_3_CN (5:1) because a cross correlation was observed between proton 9u‐H of the copper(I)‐loaded phenanthroline (at *δ*=6.96 ppm) and proton 9c‐H of the HETTAP complexed phenanthroline signal (at *δ*=5.98 ppm).^29^ The activation parameters for the corresponding exchange were determined at 298 K (*k*_298_=0.55 s^−1^ and Δ*G* ^≠^_298_=74.8 kJ mol^−1^) (SI, Figure S25).

From the activation data of rotor [Cu_2_(**A**)(**B**)]^2+^ (Δ*G* ^≠^_298_=45.5 kJ mol^−1^) and those of previously reported rotors (46.6 kJ mol^−1^),^26^ we may safely conclude that the fast exchange is the result of the cleavage of a single axial *N*_py_→ZnPor interaction. In order to achieve a better understanding of the rate‐limiting step in the slow exchange process involving the HETTAP site, we self‐assembled the monocopper complex [Cu(**A**)(**B**)]^+^ from [Cu(CH_3_CN)_4_]PF_6_ and ligands **A** and **B** in 1:1:1 ratio in *d*_2_‐dichloromethane at 25 °C. Two sets of phenanthroline signals and a single set of ZnPor signals were observed in the ^1^H NMR spectrum (Figure 4) of [Cu(**A**)(**B**)]^+^ at room temperature similar to double rotor [Cu_2_(**A**)(**B**)]^2+^. However, there was no cross correlation between the copper(I)‐loaded and free phenanthroline signals of rotor [Cu(**A**)(**B**)]^+^ in the ^1^H–^1^H ROESY spectrum (Supporting Information, Figure S26). This finding suggested that the copper(I) ion does not travel along with the terpyridine (tpy) unit. Thus, in [Cu_2_(**A**)(**B**)]^2+^ the tpy→[Cu(phenAr_2_)]^+^ dissociation seems to be relevant in the rate‐limiting step of exchange at the HETTAP site and not the phenAr_2_→[Cu(tpy)]^+^ dissociation. This interpretation is in line with the relative binding constants in mixed terpyridine–phenanthroline copper(I) complexes, since phenanthroline is more strongly bound (log*K* ${}_{\left[\mathrm{Cu}\left(1\right)\right]{}^{+}}$ =5.1) than terpyridine (log*K* ${}_{3\to \left[\mathrm{Cu}\left(1\right)\right]{}^{+}}$ =4.2).^19^, ^28^, ^30^

**Figure 4:**
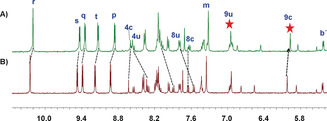
Partial ^1^H NMR spectra (400 MHz, 298 K) of A) [Cu_2_(**A**)(**B**)]^2+^, and B) [Cu(**A**)(**B**)]^+^ in CD_2_Cl_2_/CD_3_CN (5:1).

After a successful demonstration of the dynamic behavior of homodimeric and heteromeric domino rotors, their catalytic activity as catalysts was tested. Both two‐axle rotors display at any given time coordinatively free copper(I) phenanthroline units that have the potential to catalyze a click reaction. Thus, a mixture of [Cu_4_(**A**)_2_]^4+^ (0.90 mm), **5**, and **6** in 1:20:20 ratio was dissolved in CD_2_Cl_2_/CD_3_CN (5:1) and heated at 50 °C (Figure 5). After 2 h of heating, 63 % of the click product **7** was detected by ^1^H NMR analysis (Supporting Information, Chapter 6). Analogously, [Cu(**1**)]^+^ (3.6 mm; the fourfold concentration accounts for a proper comparison with [Cu_4_(**A**)_2_]^4+^ containing four copper centers) as reference catalyst, **5**, and **6** in a 1:5:5 ratio were dissolved in CD_2_Cl_2_/CD_3_CN (5:1) and heated at 50 °C. Only 26 % of product **7** was observed after 2 h, indicating a low turnover number and sizeable product inhibition. By comparing complexes of **7** with [Cu_4_(**A**)_2_]^4+^ and model phenanthroline [Cu(**1**)]^+^, liberation of **7** into solution was observed in the rotor on the basis of ^1^H NMR signal shifts, suggesting a notable reduction of product inhibition (Supporting Information, Figure S20). In addition, it was shown that deliberate addition of product **7** to [Cu_4_(**A**)_2_]^4+^ reduced the yield of the catalytic reaction (Supporting Information, Figure S31). Actually, a linear correlation was seen between loss in the catalytic yield (%) and the amount (mol %) of externally added product (Figure 5 b). The effect of higher turnover number due to increased product liberation in the fast rotor [Cu_4_(**A**)_2_]^4+^ comes as no surprise. Similar multicomponent rotors^31^ have emerged as an attractive class of catalysts because they are able to mimic sophisticated machinery from nature, such as ATP synthase,^4^ in their ability to suppress product inhibition through a nanomechanical motion. Thus rotating catalysts, even when rotation is stochastic, are more efficient than their static prototypes,^32^ a result that we see confirmed by comparing [Cu_4_(**A**)_2_]^4+^ with [Cu(**1**)]^+^ using accurately the same amount of copper(I) (Table 1).

**Figure 5:**
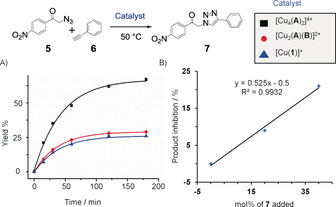
Effect of speed changes on the yield of the click reaction. A) Product formation as function of time allowing determination of the initial rate *v*_0_ (Supporting Information, Chapter 8) of the catalyzed click reaction between **5** and **6**. B) Amount of product inhibition (%) in the presence of deliberately added amounts of product **7** to the reaction mixture (analyzed after 30 min of reaction time).

**Table 1:** Exchange frequency of catalysts, their catalytic reaction yields, and the rates *v*_0_ at time *t*=0. 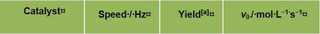

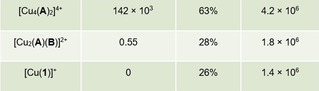

[a] Yields determined from three independent runs. For conditions see text and the Supporting Information, Chapter 6.

In comparison, a mixture of the heteromeric two‐axle rotor [Cu_2_(**A**)(**B**)]^2+^ (1.8 mm), **5**, and **6** in a 1:10:10 ratio of furnished rather low yield (28 %) under analogous conditions. This effect on the yield and the low rotational exchange (*k*_298_=0.55 s^−1^) at the copper(I) sites are consonant to those of the reference reaction; apparently a slow motion does not provide a significant reduction of product inhibition. The faster the rotational exchange at the active copper(I)‐loaded phenanthroline stations, the higher the catalytic activity,^32^ actually also with other acetylenes (Figure S33).

In conclusion, we have fabricated a new class of domino nanorotors with two fluxional axles. The rotational dynamics in the homodimeric two‐axle double rotor is governed by the dissociation of the *N*_py_→[Cu(phenAr_2_)]^+^ interaction. The full exchange at all four copper phenanthroline sites requires that both pyridine arms act as fluxional axles. In the case of the heteromeric two‐axle rotor, rotational exchange at the HETTAP site is 1.2×10^5^ times slower than the swapping of the pyridine arm between the two ZnPor units. Here we were able to individually control both fluxional axles and their dynamics.

Finally, we have utilized the speed change on going from the homodimeric to heteromeric two‐axle rotor to modulate the product inhibition of a click reaction. Attractive future goals are to modify the distinct rotational modes in heteromeric domino rotors by changing the components (**A** or **B** or metal ions, for example, Ag^+^, Zn^2+^) and to implement more than two fluxional axles in domino rotation.

## Conflict of interest

The authors declare no conflict of interest.

## Supporting information

As a service to our authors and readers, this journal provides supporting information supplied by the authors. Such materials are peer reviewed and may be re‐organized for online delivery, but are not copy‐edited or typeset. Technical support issues arising from supporting information (other than missing files) should be addressed to the authors.

Supplementary

Supplementary

Supplementary
