# Supplementary figures and images for: Double Rotors with Fluxional Axles: Domino Rotation and Azide–Alkyne Huisgen Cycloaddition Catalysis
Source: Angew Chem Int Ed Engl. 2020 Jun 10;59(30):12362–6. doi: 10.1002/anie.202002739 (PMC7383839; doi:10.1002/anie.202002739)

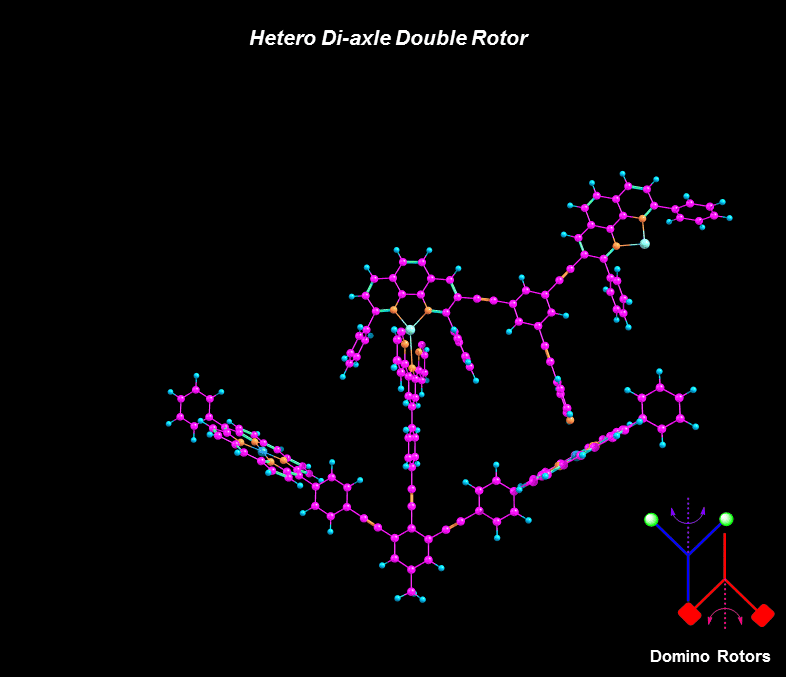

Supplement: Supplementary file 1 — Supplementary [file ANIE-59-12362-s001.gif]
