# Supplementary material for: Double Rotors with Fluxional Axles: Domino Rotation and Azide–Alkyne Huisgen Cycloaddition Catalysis
Source: Angew Chem Int Ed Engl. 2020 Jun 10;59(30):12362–6. doi: 10.1002/anie.202002739 (PMC7383839; doi:10.1002/anie.202002739)
Supplement: Supplementary file 2 — Supplementary [file ANIE-59-12362-s002.pdf]

Angewandte Chemie
Eine Zeitschrift der Gesellschaft Deutscher Chemiker

Supporting Information

Double Rotors with Fluxional Axles: Domino Rotation and Azide–Alkyne Huisgen Cycloaddition Catalysis

*Abir Goswami and Michael Schmittel**

anie_202002739_sm_miscellaneous_information.pdf

Table of Contents

1. Synthesis………………………………………………………………….. S2-S5
2. Synthesis and characterization of complexes……………………………….. S6-S11
3. NMR spectra………………………………………………………………… S12-S21
4. DOSY NMR spectra………………………………………………………… S22-S23
5. Variable temperature study, ROESY and determination of kinetic parameters… S24-S27
6. Catalytic experiments……………………………………………………….. S28-S32
7. ESI-MS spectra……………………………………………………………… S33-S37
8. Elucidation of v_0 for the reactions using different catalyst ……………………….S37
9. UV-Vis data…………………………………………………………………..S37-S39
10. Computational data…………………………………………………………..S40-S48
11. References……………………………………………………………………….S48

1. Synthesis

General Remarks

All solvents were dried by distillation prior to use while commercial reagents (**8**, **9**, **12**) were used without any further purification. Bruker Avance (400 MHz), Jeol ECZ 500 and Varian VNMR-S 600 (600 MHz) spectrometers were used to measure ^{1}H and ^{13}C NMR spectra applying the deuterated solvent as the lock and residual protiated solvent as internal reference ($CDCl_3$: δ_H 7.26 ppm, δ_C 77.0 ppm; CD_2Cl_2: δ_H 5.32 ppm, δ_C 53.8 ppm, THF-d_8: δ_H 1.72 ppm, 3.58 ppm, δ_C 25.3 ppm, 67.2 ppm). The following abbreviations were used to define NMR peak pattern: s = singlet, d = doublet, t = triplet, dd = doublet of doublets, ddd = doublet of doublets of doublets, td = triplet of doublets, br = broad, m= multiplet. Coupling constant values are given in Hertz (Hz) and, wherever possible, assignment of protons is provided. The numbering of different carbons in different molecular skeletons does not necessarily follow IUPAC nomenclature rules; it was exclusively implemented for assigning NMR signals. All electrospray ionization (ESI-MS) spectra were recorded on a Thermo-Quest LCQ deca and theoretical isotopic distributions of the mass signals were calculated using IsoPro 3.0 software. Melting points of compounds were measured on a BÜCHI 510 instrument and are not corrected. Infrared spectra were recorded on a Perkin Elmer Spectrum Two FT-IR instrument. Elemental analysis was performed using the EA-3000 CHNS analyzer. UV-vis spectra were recorded on a Cary Win 50 (298 K) spectrometer. Binding constants were determined through UV-vis titrations in combination with a 1:1 binding formula of two ligands or with SPECFIT/32TM global analysis system by Spectrum Software Associates (Marlborough, MA). Column chromatography was performed either on silica gel (60-400 mesh) or neutral alumina (Fluka, 0.05-0.15 mm, Brockmann Activity 1). Merck silica gel (60 F254) or neutral alumina (150 F254) sheets were used for thin layer chromatography (TLC). All rotor preparations were performed directly in the NMR tube using CD_2Cl_2 as solvent. Compounds **B**,[1] **11**,[2] were synthesized according to literature known procedures.

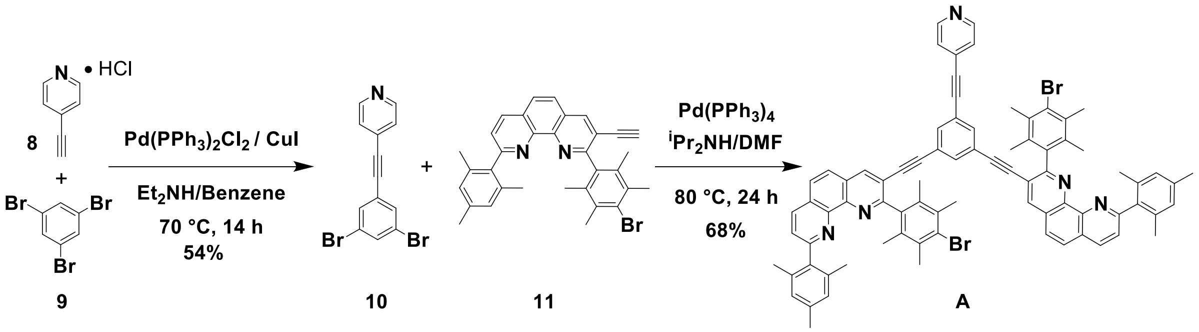

Scheme 1. Synthesis of ligand **A**.

4-((3,5-Dibromophenyl)ethynyl)pyridine (10)

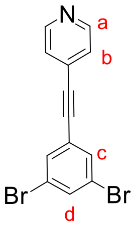

10

In an oven-dried 100 mL sealed tube, 1,3,5-tribromobenzene (**9**) (2.00 g, 6.35 mmol) and 4-ethynylpyridine hydrochloride (**8**) (500 mg, 3.58 mmol) were dissolved in dry benzene (30 mL) and Et_2NH (20 mL) and thoroughly deaerated by purging with N_2. Then $Pd(PPh_3)_2Cl_2$ (250 mg, 0.356 mmol) and CuI (80.0 mg, 0.420 mmol) were added and the mixture was refluxed at 70 °C for 14 h. The reaction mixture was cooled down to room temperature and the solvents were removed. The residue was subjected to column chromatography (silica gel, CH_2Cl_2, R_f = 0.3) to afford 650 mg of compound **10** as colorless solid (1.92 mmol, 54%). **Melting point** = 216 °C. **IR (KBr):** $\tilde{\nu}$ = 437, 477, 538, 571, 651, 727, 792, 819, 911, 993, 1062, 1091, 1147, 1204, 1315, 1396, 1403, 1417, 1489, 1527, 1547, 1923, 2157, 2221, 2955, 3051 cm^{-1}. **^{1}H NMR (500 MHz, $CDCl_3$):** δ = 7.36 (d, ^3J = 5.8 Hz, 2H, b-H), 7.62 (d, ^4J = 1.8 Hz, 2H, c-H), 7.68 (t, ^4J = 1.8 Hz, 1H, d-H), 8.62 (d, ^3J = 5.8 Hz, 2H, a-H) ppm. **^{13}C NMR (100 MHz, $CDCl_3$):** δ = 89.6, 91.3, 123.6, 126.2, 126.3, 131.2, 134.0, 135.7, 150.7 ppm. **ESI-MS:** *m/z* (%) 338.2 (100) [**10** + H]$^+$. **Elemental analysis:** Calculated for $C_{13}H_7Br_2N$: C, 46.33; H, 2.09; N, 4.16. Found: C, 46.41; H, 1.92; N, 4.19.

3,3'-(5-(Pyridin-4-ylethynyl)-1,3-phenylene)bis(ethyne-2,1-diyl)bis(2-(4-bromo-2,3,5,6-tetramethylphenyl)-9-mesityl-1,10-phenanthroline) (A)

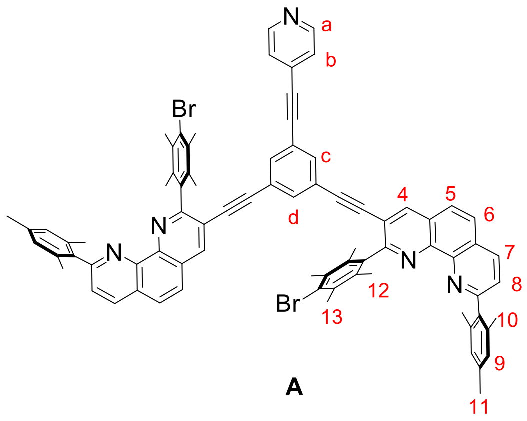

A

Compound **10** (80.0 mg, 0.237 mmol) and 2-(4-bromo-2,3,5,6-tetramethylphenyl)-3-ethynyl-9-mesityl-1,10-phenanthroline (**11**) (400 mg, 0.750 mmol) were taken in oven-dried 100 mL tube and dissolved in dry DMF (20 mL) and $^{i}Pr_2NH$ (20 mL). The solution was degassed twice by using the freeze-pump-thaw method. Finally, $Pd(PPh_3)_4$ (28.0 mg, 24.0 µmol) was added under N_2 atmosphere and the mixture was allowed to stir at 80 °C for 24 h followed by evaporation to dryness. The crude product was purified by column chromatography (aluminum oxide (neutral), CH_2Cl_2, R_f = 0.3) providing 200 mg of **A** as colorless solid (16.1 µmol, 68%). **Melting point** >250 °C; **IR (KBr):** $\tilde{\nu}$ = 537, 601, 643, 681, 792, 828, 853, 897, 927, 987, 1147, 1169, 1216, 1382, 1453, 1471, 1537, 1592, 1931, 2213, 2913, 3059 cm^{-1}. **^{1}H NMR (400 MHz, $CDCl_3$):** δ = 2.02 (s, 12H, 13-H), 2.10 (s, 12H, 10-H), 2.31 (s, 6H, 11-H), 2.49 (s, 12H, 12-H), 6.89 (t, ^4J = 1.6 Hz, 1H, d-H), 6.93 (s, 4H, 9-H), 7.03 (d, ^4J = 1.6 Hz, 2H, c-H), 7.47 (d, ^3J = 5.8 Hz, 2H, b-H), 7.60 (d, ^3J = 8.0 Hz, 2H, 8-H), 7.88 (d, ^3J = 8.8 Hz, 2H, 6/5-H), 7.93 (d, ^3J = 8.8 Hz, 2H, 5/6-H), 8.31 (d, ^3J = 8.0 Hz, 2H, 7-H), 8.51 (s, 2H, 4-H), 8.66 (d, ^3J = 5.8 Hz, 2H, a-H) ppm. **^{13}C NMR (100 MHz, $CDCl_3$):** δ = 18.6, 20.5, 21.0, 21.1, 87.8, 88.6, 91.9, 93.5, 119.4, 123.1, 123.7, 125.4, 125.6, 125.7, 126.8, 127.3, 127.7, 128.5, 129.3, 131.0, 133.6, 133.8, 134.1, 134.4, 135.9, 136.1, 137.6, 137.9, 138.5, 139.1, 145.0, 145.9, 149.7, 160.7, 162.6 ppm. **ESI-MS:** *m/z* (%) 1242.5 (100) [**A** + H]$^+$. **Elemental analysis:** Calcd. for $C_{79}H_{63}Br_2N_5 \cdot 2CH_2Cl_2$: C, 68.90; H, 4.78; N, 4.96. Found: C, 69.19; H, 4.71; N, 5.06.

Characterization Data of Stator (B) [1]

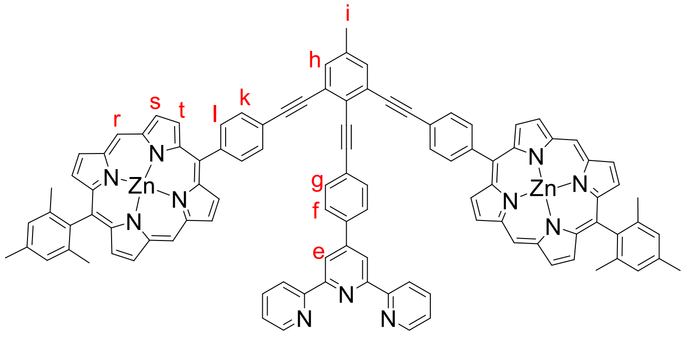

B

Melting point: > 250 °C. **IR (KBr):** $\tilde{\nu}$ = 564, 608, 634, 702, 720, 734, 785, 813, 833, 858, 906, 995, 1059, 1142, 1212, 1287, 1315, 1391, 1439, 1496, 1521, 1608, 2227, 2915 cm^{-1}. **^{1}H NMR (400 MHz, CD_2Cl_2):** δ = 1.82 (s, 12H, n-H), 2.62 (s, 3H, i-H), 2.70 (s, 6H, o-H), 7.11 (ddd, ^3J = 8.0 Hz, ^3J = 5.0 Hz, ^4J = 1.2 Hz, 2H, b-H), 7.38 (s, 4H, m-H), 7.70 (td, ^3J = 8.0 Hz, ^4J = 1.2 Hz, 2H, c-H), 7.73 (s, 2H, h-H), 8.11 (d, ^3J = 8.4 Hz, 2H, g/f-H), 8.15 (d, ^3J = 8.4 Hz, 2H, f/g-H), 8.19 (d, ^3J = 8.0 Hz, 4H, k-H), 8.33 (ddd, ^3J = 5.0 Hz, ^4J = 1.2 Hz, ^5J = 0.8 Hz, 2H, a-H), 8.38 (d, ^3J = 8.0 Hz, 4H, l-H), 8.47 (ddd, ^3J = 8.0 Hz, ^4J = 1.2 Hz, ^5J = 0.8 Hz, 2H, d-H), 8.75 (s, 2H, e-H), 8.95 (d, ^3J = 4.4 Hz, 4H, p-H), 9.23 (d, ^3J = 4.4 Hz, 4H, t-H), 9.43 (d, ^3J = 4.4 Hz, 4H, q-H), 9.51 (d, ^3J = 4.4 Hz, 4H, s-H), 10.28 (s, 4H, r-H). **^{13}C NMR (100 MHz, d_8-THF:CD_2Cl_2 = 4:1):** δ = 20.6, 21.0, 21.5, 89.2, 89.5, 94.3, 97.3, 105.6, 117.7, 118.4, 118.6, 120.9, 122.5, 124.0, 125.0, 125.6, 126.8, 127.7, 127.9, 130.1, 130.7, 131.7, 131.9, 132.1, 132.7, 132.7, 135.3, 136.7, 137.5, 138.9, 139.0, 139.3, 139.8, 144.5, 149.2, 149.3, 149.8, 149.8, 149.9, 149.9, 156.1, 156.4 ppm. **ESI-MS:** *m/z* (%) 1604.7 (100) [**B** + H]$^+$. **Elemental analysis:** Calcd. for $C_{104}H_{69}N_{11}Zn_2$•H_2O: C, 77.03; H, 4.41; N, 9.50.Found: C, 77.04; H, 4.15; N, 9.40.

2. Synthesis and characterization of complexes

a) Model Complex **C1 = [2→4]**

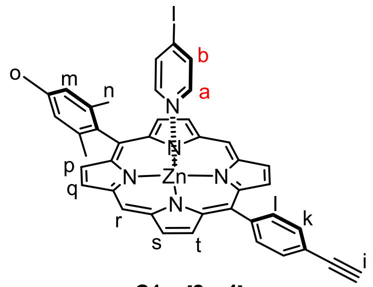

C1 = [**2**→**4**]

In an NMR tube, zinc porphyrin **4** (0.456 mg, 0.770 µmol) and 4-iodopyridine (**2**) (0.158 mg, 0.771 µmol) were dissolved in 500 µL of CD_2Cl_2 and the NMR spectra was recorded. Yield: quantitative. **Melting point:** > 200 °C; **IR (KBr):** $\tilde{\nu}$ = 558, 614, 697, 721, 787, 809, 838, 886, 909, 995, 1057, 1121, 1143,1158, 1182, 1209, 1228, 1288, 1313, 1374, 1395, 1437, 1522, 1559, 1612, 2137, 2915, 3051, 3079 cm^{-1}. **^{1}H NMR (400 MHz, CD_2Cl_2):** δ = 1.77 (s, 6H, n-H), 2.66 (s, 3H, o-H), 3.02 (brs, 2H, a-H), 3.40 (s, 1H, i-H), 6.12 (d, ^3J = 5.6 Hz, 2H, b-H), 7.33 (s, 2H, m-H), 7.93 (d, ^3J = 8.0 Hz, 2H, k-H), 8.25 (d, ^3J = 8.0 Hz, 2H, l-H), 8.88 (d, ^3J = 4.5 Hz, 2H, p-H), 9.06 (d, ^3J = 4.5 Hz, 2H, t-H), 9.36 (d, ^3J = 4.5 Hz, 2H, q-H), 9.41 (d, ^3J = 4.5 Hz, 2H, s-H), 10.21 (s, 2H, r-H) ppm.

d) Model complex **C2 =** [Cu(**1**)(**2**)]$^+$

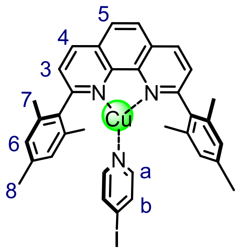

C2 = [Cu(**1**)(**2**)]$^+$

In an NMR tube, phenanthroline **1** (0.460 mg, 1.10 µmol) and 4-iodopyridine (**2**) (0.226 mg, 1.10 µmol) as well as $[Cu(CH_3CN)_4]PF_6$ (0.412 mg, 1.10 µmol) were dissolved in 500 µL of CD_2Cl_2. Yield by NMR: quantitative. **^{1}H NMR (CD_2Cl_2, 400 MHz):** δ 2.03 (s, 12H, 7-H), 2.36 (s, 6H, 8-H), 6.51 (brs, 2H, a-H), 6.97 (s, 4H, 6-H), 7.43 (brs, 2H, b-H), 7.93 (d, ^3J = 8.4 Hz, 2H, 3-H), 8.18 (s, 2H, 5-H), 8.72 (d, ^3J = 8.4 Hz, 2H, 4-H) ppm.

e) Model complex **C3** = [Cu(**1**)(**3**)]$^{+}$

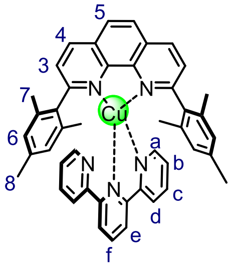

C3 = [Cu(**1**)(**3**)]$^{+}$

In an NMR tube, phenanthroline **1** (0.468 mg, 1.12 µmol), terpyridine (**6**) (0.263 mg, 1.12 µmol), and [Cu(CH_3CN)$_4$)]PF_6 (0.419 mg, 1.12 µmol) were dissolved in 500 of µL CD_2Cl_2. NMR spectra were recorded showing quantitative formation of the copper(I) HETTAP complex. **^{1}H NMR (400 MHz, CD_2Cl_2):** δ = 1.56 (s, 12H, 7-H), 1.95 (s, 6H, 8-H), 6.27 (s, 4H, 6-H), 7.03 (ddd, ^3J = 7.4 Hz, ^3J = 5.6 Hz, ^4J = 1.6 Hz, 2H, b-H), 7.48 (td, ^3J = 7.4 Hz, ^4J = 1.6 Hz, 2H, c-H), 7.59 (d, ^3J = 5.6 Hz, 2H, a-H), 7.71 (d, ^3J = 7.4 Hz, 2H, d-H), 7.75 (d, ^3J = 8.2 Hz, 2H, 3-H), 7.90 (d, ^3J = 7.4 Hz, 2H, e-H), 8.00 (t, ^3J = 7.4 Hz, 1H, f-H), 8.14 (s, 2H, 5-H), 8.57 (d, ^3J = 8.2 Hz, 2H, 4-H) ppm.

Synthesis of homodimeric double rotor C4 = [Cu$_4$(**A**)$_2$]$^{4+}$

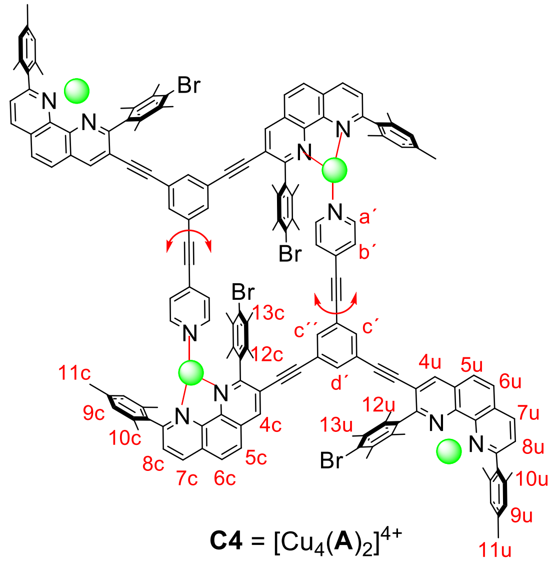

C4 = [Cu$_4$(**A**)$_2$]$^{4+}$

In an NMR tube, ligand **A** (0.660 mg, 0.531 µmol) and [Cu(CH_3CN)$_4$)]PF_6 (0.396 mg, 1.06 µmol) were dissolved in 500 µL of CD_2Cl_2. NMR spectra were measured immediately. Yield:

quantitative. **Melting point:** > 250 °C. **IR (KBr):** $\tilde{\nu}$ = 574, 635, 713, 744, 757, 781, 812, 1033, 1051, 1221, 1261, 1343, 1461, 1457, 1514, 1617, 1651, 2012, 2218, 2857, 2957 cm^{-1}. **^{1}H NMR (400 MHz, CD_2Cl_2):** δ = 2.02 (s, 24H, 13c+13u-H), 2.06 (s, 24H, 10c+10u-H), 2.36 (s, 12-H, 11c+11u-H), 2.52 (s, 24H, 12c+12u-H), 6.93 (br, 4H, a´-H), 7.01 (s, 8H, 9c+9u-H), 7.08 (br, 4H, c´+c´´-H), 7.23 (br, 6H, b´+d´-H), 7.96 (d, ^3J = 8.0 Hz, 4H, 8c+8u-H), 8.22 (s, 8H, 5c+6c+5u+6u-H), 8.72 (d, ^3J = 8.0 Hz, 4H, 7c+7u-H), 8.90 (s, 4H, 4c+4u-H), ppm. **ESI-MS:** *m*/*z* (%) 684.8 (100) [[Cu_4(**A**)$_2$]$^{4+}$]. **Elemental analysis:** Calculated for $C_{158}H_{126}Br_4Cu_4F_{24}N_{10}P_4$•$CH_2Cl_2$•$3H_2O$: C, 55.24; H, 3.91; N, 4.05. Found: C, 55.23; H, 3.60; N, 3.97.

Synthesis of nanorotor C5 = [Cu(**A**)(**B**)]$^+$

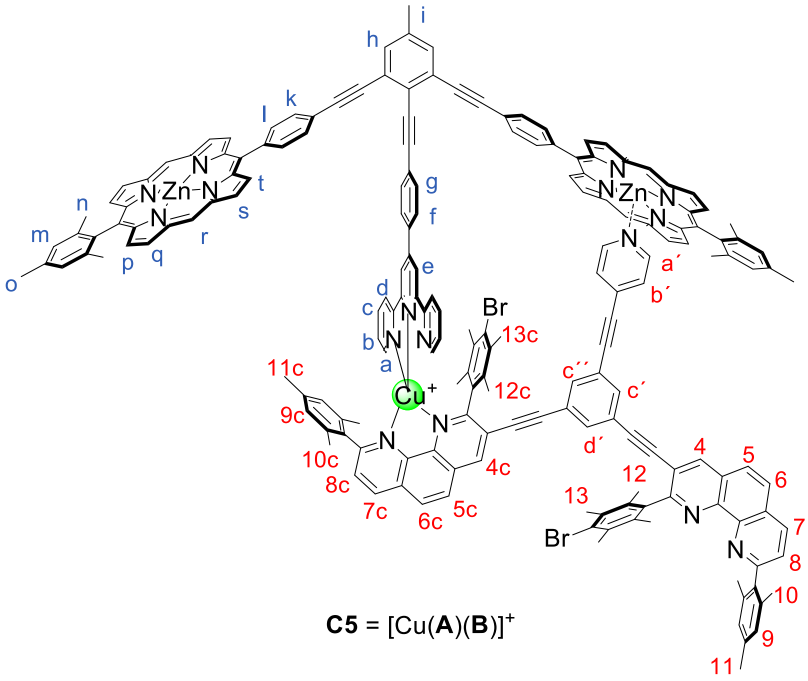

C5 = [Cu(**A**)(**B**)]$^+$

In an NMR tube, ligand **B** (0.533 mg, 0.332 µmol), **A** (0.412 mg, 0.332 µmol) and [Cu(CH_3CN)$_4$)]PF_6 (0.123 mg, 0.333 µmol) were dissolved in 500 µL of CD_2Cl_2 and ^{1}H NMR spectra were measured immediately. NMR and ESI-MS spectra confirm quantitative formation of nanorotor **C5** = [Cu(**A**)(**B**)]$^+$. **Melting point** > 250 °C. **IR (KBr):** $\tilde{\nu}$ = 512, 568, 653, 732, 738, 756, 843, 954, 1027, 1122, 1155, 1209, 1221, 1243, 1322, 1397, 1445, 1517, 1547, 1615, 2216, 2853, 2921, 3016 cm^{-1}. **^{1}H NMR (600 MHz, CD_2Cl_2):** δ = 1.24 (s, 6H, 13c-H), 1.32 (s,

6H, 10c-H), 1.41 (s, 3H, 11c-H), 1.54 (merged with H_2O, 6H, 12c-H), 1.86 (s, 18H, n+13-H), 2.03 (s, 6H, 10-H), 2.24 (d, ^3J = 6.4 Hz, 2H, a´-H), 2.31 (s, 6H, 12-H), 2.37 (s, 3H, 11-H), 2.65 (s, 3H, i-H), 2.70 (s, 6H, o-H), 5.48 (d, ^3J = 6.4 Hz, 2H, b´-H), 5.91 (s, 1H, c´´-H), 6.06 (s, 2H, 9c-H), 6.60 (s, 1H, c´-H), 6.82 (s, 1H, d´-H), 6.98 (s, 2H, 9-H), 7.00 (ddd, ^3J = 7.8 Hz, ^3J = 4.8 Hz, ^4J = 1.2 Hz, 2H, b-H), 7.39 (s, 4H, m-H), 7.38-7.47 (m, 4H, a+c-H), 7.59 (d, ^3J = 8.8 Hz, 1H, 8-H), 7.69 (d, ^3J = 8.8 Hz, 1H, 8c-H), 7.78 (s, 2H, h-H), 7.82 (dd, ^3J = 7.8 Hz, ^4J = 1.2 Hz, 2H, d-H), 7.83 (d, ^3J = 8.8 Hz, 1H, 6/5-H), 7.93 (d, ^3J = 8.8 Hz, 1H, 5/6-H), 8.01(d, ^3J = 8.0 Hz, 2H, f/g-H), 8.15 (d, ^3J = 8.8 Hz, 1H, 6c/5c-H), 8.18 (s, 2H, e-H), 8.19 (d, ^3J = 8.8 Hz, 1H, 5c/6c-H), 8.21 (d, ^3J = 8.0 Hz, 2H, g/f -H), 8.23 (d, ^3J = 8.0 Hz, 4H, k-H), 8.34 (d, ^3J = 8.8 Hz, 1H, 7-H), 8.37 (s, 1H, 4-H), 8.43 (d, ^3J = 8.0 Hz, 4H, l-H), 8.58 (d, ^3J = 8.8 Hz, 1H, 7c-H), 8.67 (s, 1H, 4c-H), 8.97 (d, ^3J = 4.4 Hz, 4H, p-H), 9.22 (d, ^3J = 4.4 Hz, 4H, t-H), 9.43 (d, ^3J = 4.4 Hz, 4H, q-H), 9.52 (d, ^3J = 4.4 Hz, 4H, s-H), 10.29 (s, 4H, r-H) ppm. **ESI-MS:** *m*/*z* (%) 2908.7 (100) [Cu(**A**)(**B**)]$^+$, 1454.6 (50) [Cu(**A**)(**B**)(H)]$^{2+}$. **Elemental analysis:** Calculated for $C_{183}H_{132}Br_2CuF_6N_{16}PZn_2$•$CH_2Cl_2$•$H_2O$: C, 68.53; H, 4.29; N, 6.91. Found: C, 68.29; H, 4.01; N, 6.77.

Synthesis of nanorotor C6 = [Cu_2(**A**)(**B**)]$^{2+}$

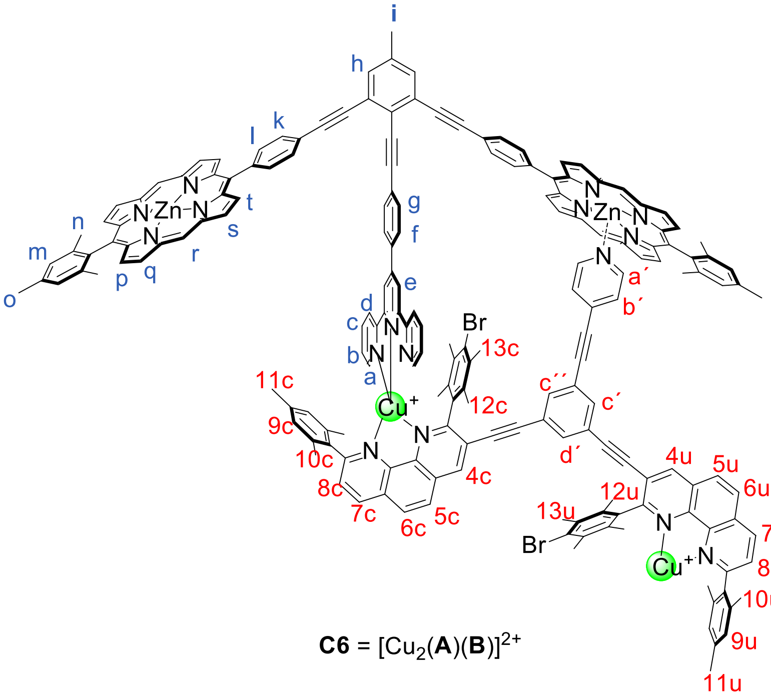

C6 = [Cu_2(**A**)(**B**)]$^{2+}$

In an NMR tube, ligand **B** (0.853 mg, 0.532 μmol), **A** (0.661 mg, 0.532 μmol) and $[Cu(CH_3CN)_4)]PF_6$ (0.397 mg, 1.06 μmol) were dissolved in 500 μL of CD_2Cl_2 and ^{1}H NMR spectra were measured immediately. NMR and ESI-MS spectra confirm quantitative formation of nanorotor **C6** = $[Cu_2$(**A**)(**B**)$]^{2+}$. **Melting point** > 250 °C. **IR (KBr):** $\tilde{\nu}$ = 518, 681, 703, 714, 724, 782, 811, 841, 992, 1027, 1102, 1128, 1224, 1253, 1334, 1397, 1445, 1513, 1542, 1601, 2213, 2882, 2922, 3012 cm^{-1}. **^{1}H NMR (600 MHz, CD_2Cl_2:CD_3CN (5:1)):** δ = 1.18 (s, 6H, 13c-H), 1.26 (s, 6H, 10c-H), 1.47 (s, 3H, 11c-H), 1.51 (s, 6H, 12c-H), 1.76 (s, 6H, 13u-H), 1.90 (s, 12H, n-H), 1.54 (merged with water, 6H, 10u-H), 2.20 (d, ^3J = 6.4 Hz, 2H, a´-H), 2.25 (s, 6H, 12u-H), 2.34 (s, 3H, 11u-H), 2.59 (s, 3H, i-H), 2.64 (s, 6H, o-H), 5.44 (d, ^3J = 6.4 Hz, 2H, b´-H), 5.86 (s, 1H, c´´-H), 5.98 (s, 2H, 9c-H), 6.47 (s, 1H, c´-H), 6.85 (s, 1H, d´-H), 6.94 (ddd, ^3J = 7.8 Hz, ^3J = 4.8 Hz, ^4J = 1.2 Hz, 2H, b-H), 6.96 (s, 2H, 9u-H), 7.39 (s, 4H, m-H), 7.39-7.45 (m, 4H, a+c-H), 7.64 (d, ^3J = 8.8 Hz, 1H, 8c-H), 7.72 (s, 2H, h-H), 7.80 (d, ^3J = 8.8 Hz, 1H, 8u-H), 7.81 (dd, ^3J = 7.8 Hz, ^4J = 1.2 Hz, 2H, d-H), 7.99 (d, ^3J = 8.0 Hz, 2H, f/g -H), 8.09 (d, ^3J = 8.8 Hz, 1H, 6u/5u-H), 8.15 (d, ^3J = 8.0 Hz, 2H, g/f-H), 8.15-8.17 (m, 5H, 5u/6u+k-H), 8.17 (s, 2H, e-H), 8.18 (d, ^3J = 8.8 Hz, 1H, 5c/6c-H), 8.19 (d, ^3J = 8.8 Hz, 1H, 6c/5c-H), 8.38 (d, ^3J = 8.0 Hz, 2H, l-H), 8.56 (d, ^3J = 8.8 Hz, 1H, 7u-H), 8.58 (s, 1H, 4u-H), 8.57 (d, ^3J = 8.8 Hz, 1H, 7c-H), 8.61 (s, 1H, 4c-H), 8.87 (d, ^3J = 4.4 Hz, 4H, p-H), 9.15 (d, ^3J = 4.4 Hz, 4H, t-H), 9.36 (d, ^3J = 4.4 Hz, 4H, q-H), 9.45 (d, ^3J = 4.4 Hz, 4H, s-H), 10.23 (s, 4H, r-H) ppm. **ESI-MS:** *m*/*z* (%) 1486.1 (100) [Cu(**A**)(**B**)$]^{2+}$. **Elemental analysis:** Calculated for $C_{183}H_{132}Br_2Cu_2F_{12}N_{16}P_2Zn_2$•$2CH_2Cl_2$: C, 64.73; H, 3.99; N, 6.53. Found: C, 64.87; H, 3.70; N, 6.62.

Characterization of C7 = [Cu(**1**)(**7**)$]^+$:

^{1}H NMR (400 MHz, CD_2Cl_2): δ = 1.91 (s, 12H, 10-H), 2.24 (s, 6H, 11-H), 5.98 (s, 2H, c-H), 6.77 (s, 2H, 9-H), 7.31-7.35 (m, 3H, [f+g]-H), 7.43-7.46 (m, 2H, e-H), 7.89 (d, ^3J = 8.2 Hz, 2H, 3-H), 7.92 (s, 1H, d-H), 8.14 (s, 2H, 5-H), 8.28 (d, ^3J = 8.4 Hz, 2H, a-H), 8.43 (d, ^3J = 8.4 Hz, 2H, b-H), 8.66 (d, ^3J = 8.2 Hz, 2H, 4-H). **ESI-MS:** *m*/*z* (%) 787.7 (100) [Cu(**1**)(**7**)$]^+$.

3. NMR spectra

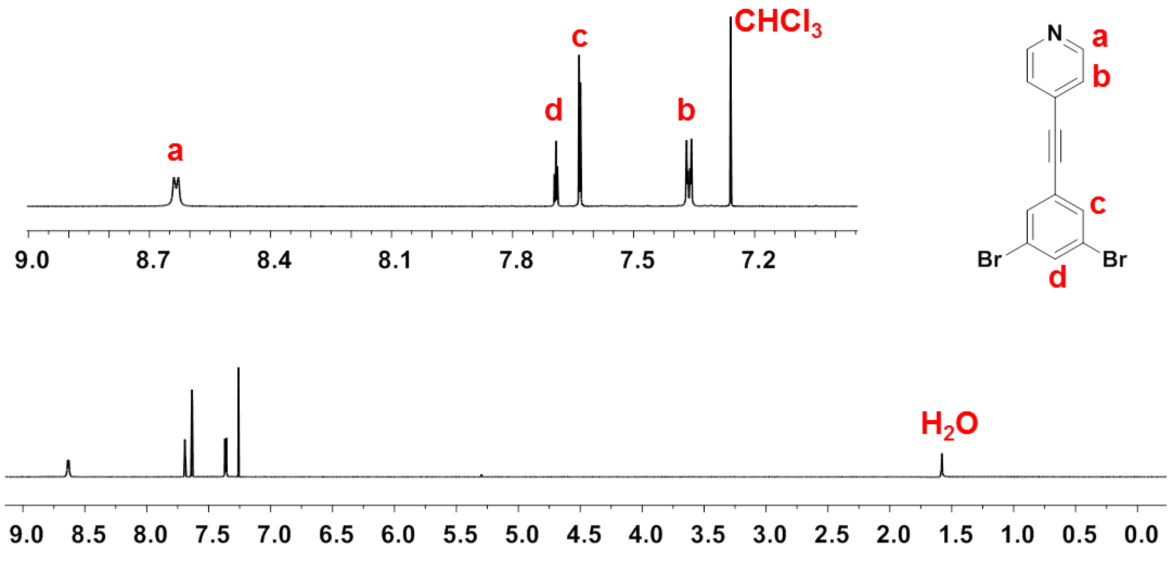

Figure S1. ^{1}H NMR spectrum of **10** in $CDCl_3$ (500 MHz, 298 K).

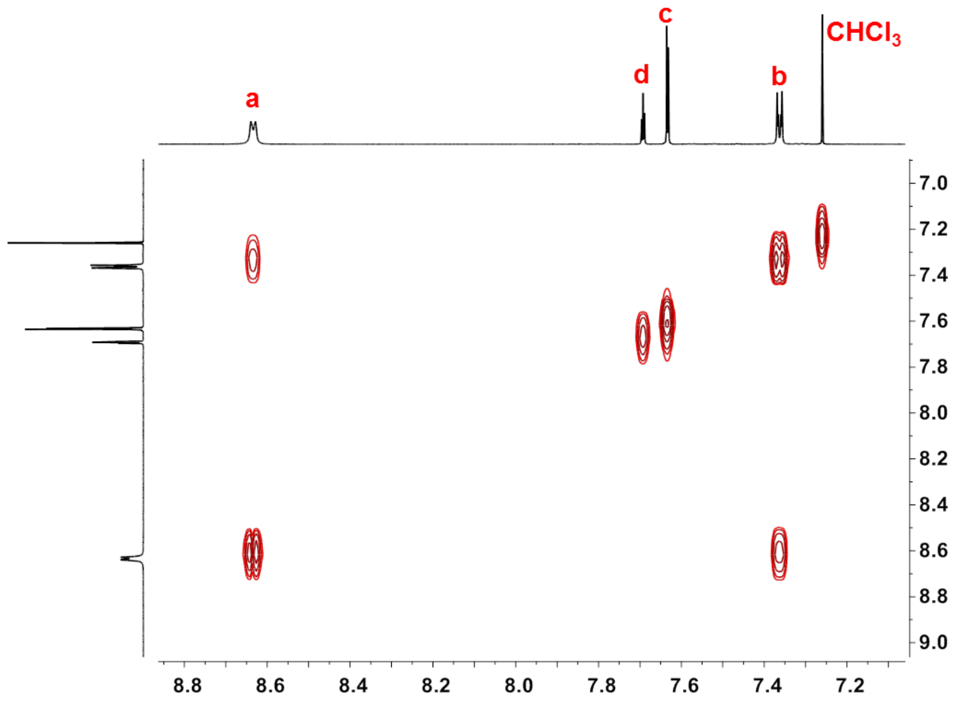

Figure S2. ^{1}H-^{1}H COSY spectrum of **10** in $CDCl_3$ (500 MHz, 298 K).

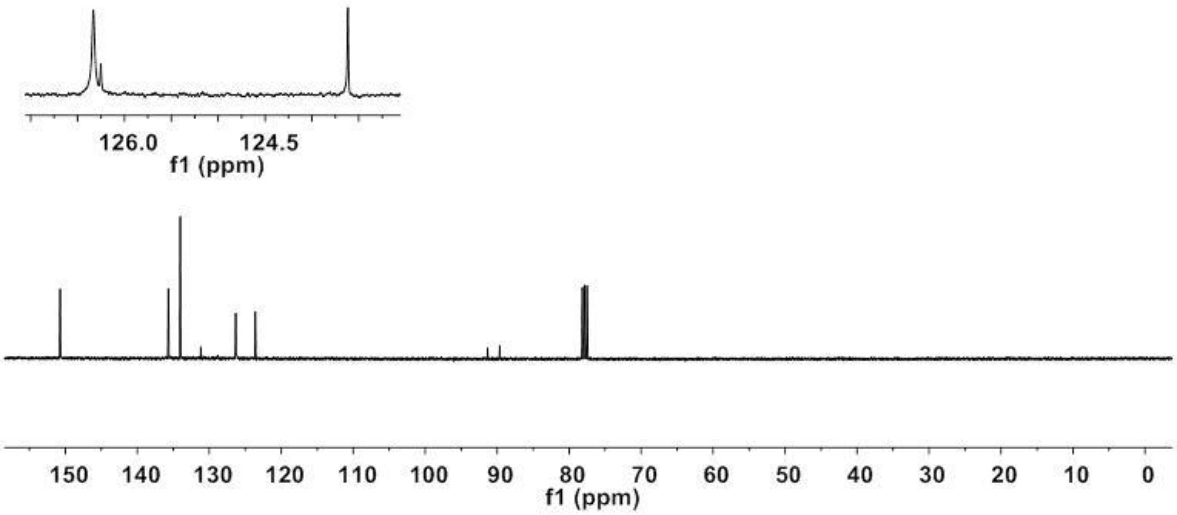

Figure S3. ^{13}C NMR spectrum of **10** in $CDCl_3$ (100 MHz, 298 K).

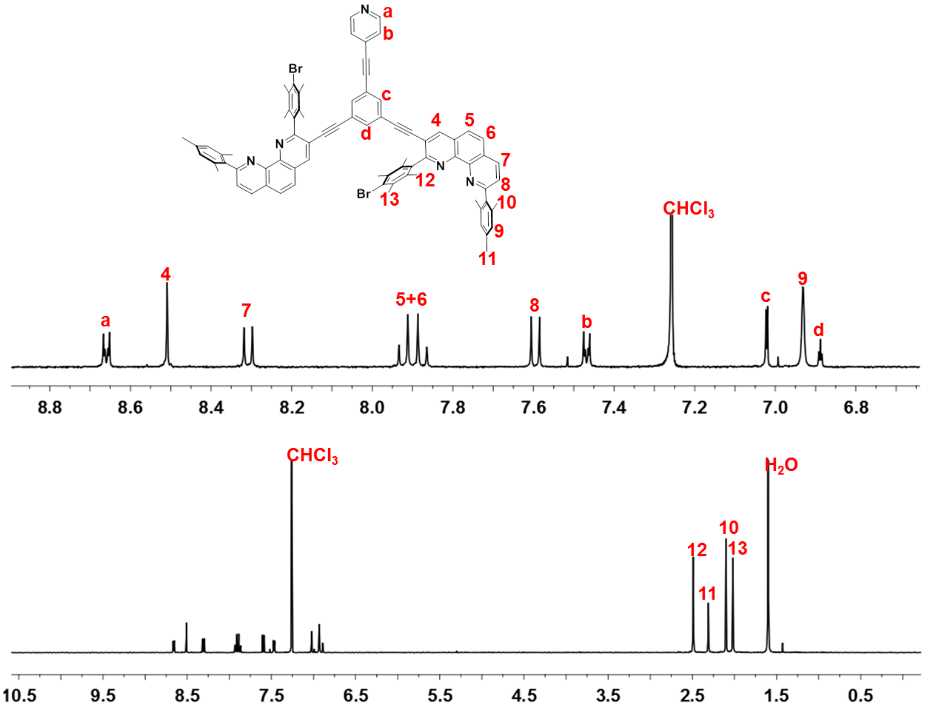

Figure S4. ^{1}H NMR spectrum of **A** in $CDCl_3$ (400 MHz, 298 K).

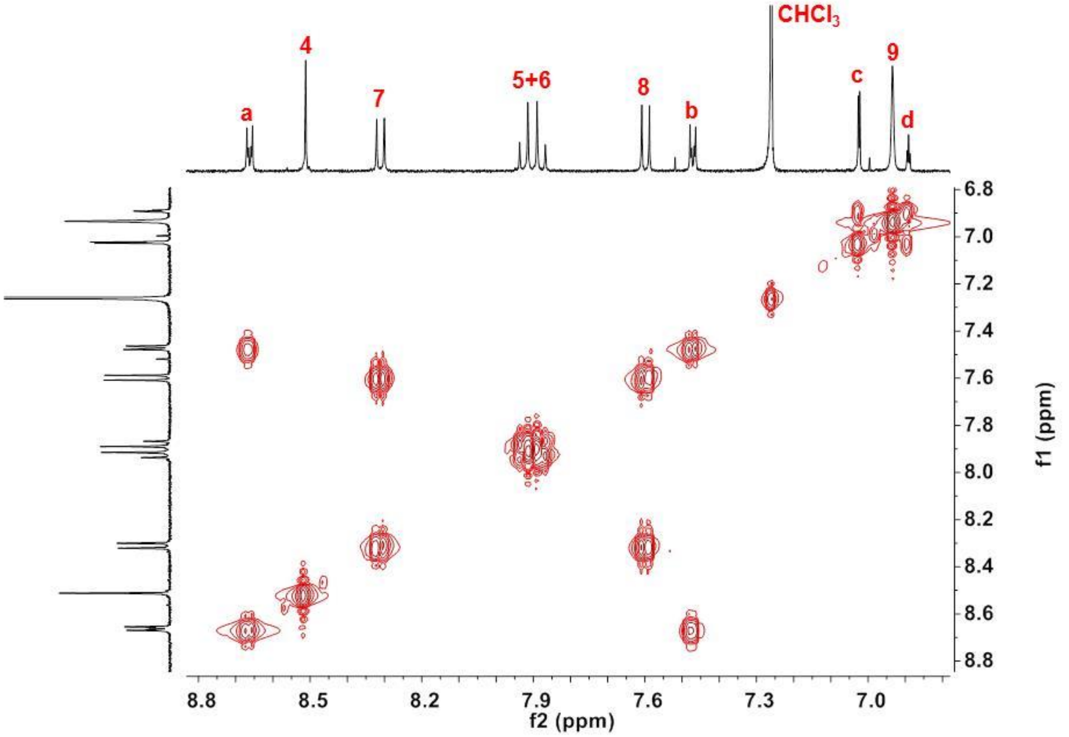

Figure S5. ^{1}H-^{1}H COSY spectrum of **A** in $CDCl_3$ (400 MHz, 298 K).

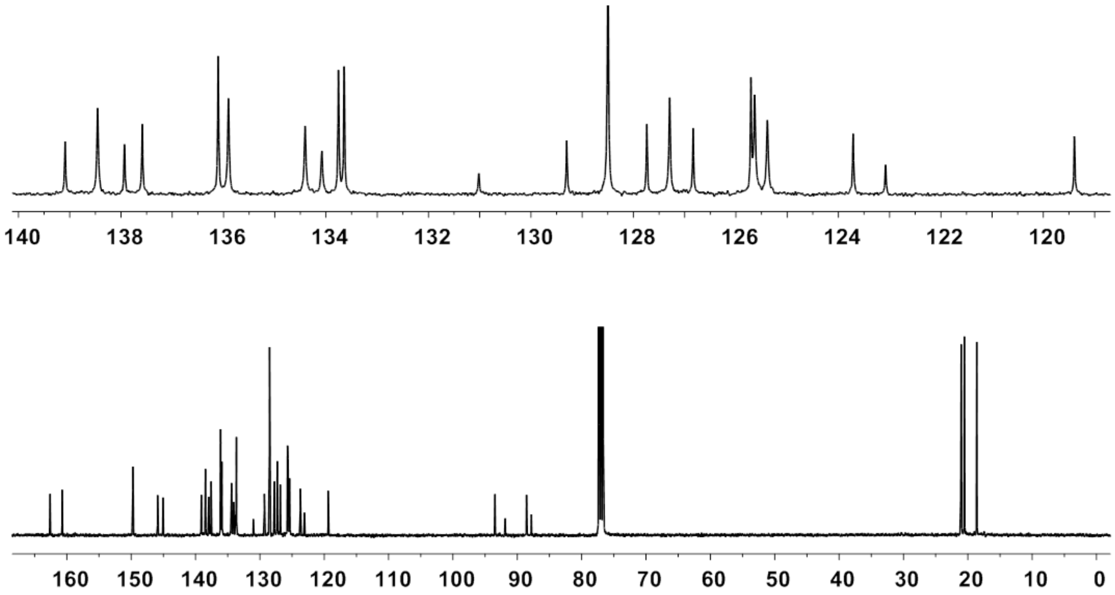

Figure S6. ^{13}C NMR spectrum of **A** in $CDCl_3$ (100 MHz, 298 K).

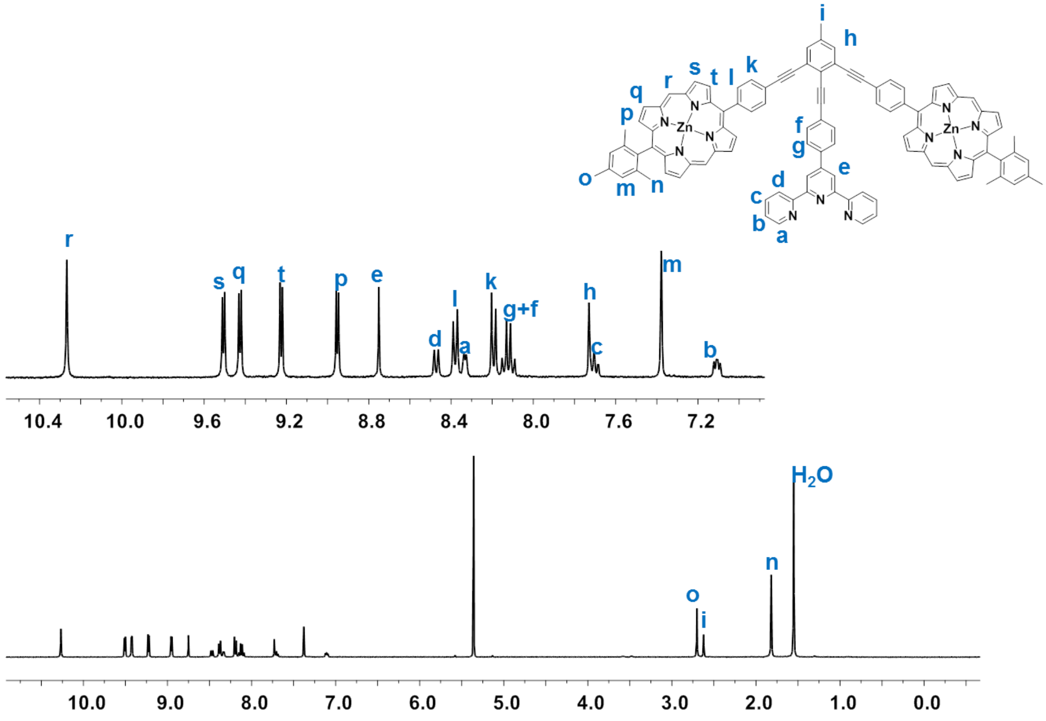

Figure S7. ^{1}H NMR spectrum of stator **B** in CD_2Cl_2 (400 MHz, 298 K).

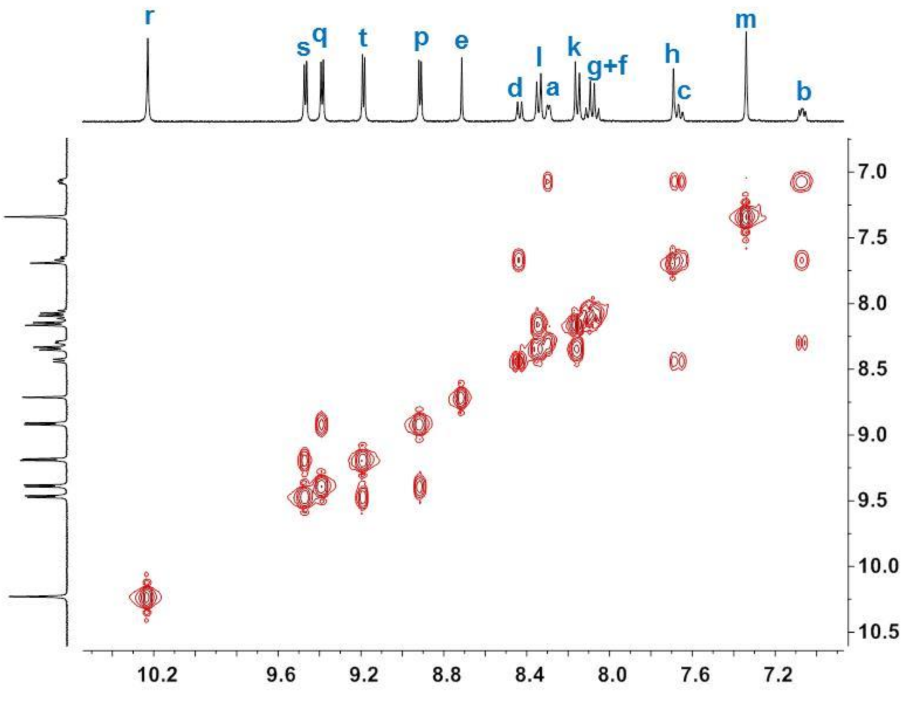

Figure S8. ^{1}H-^{1}H COSY spectrum of stator **B** in CD_2Cl_2 (400 MHz, 298 K).

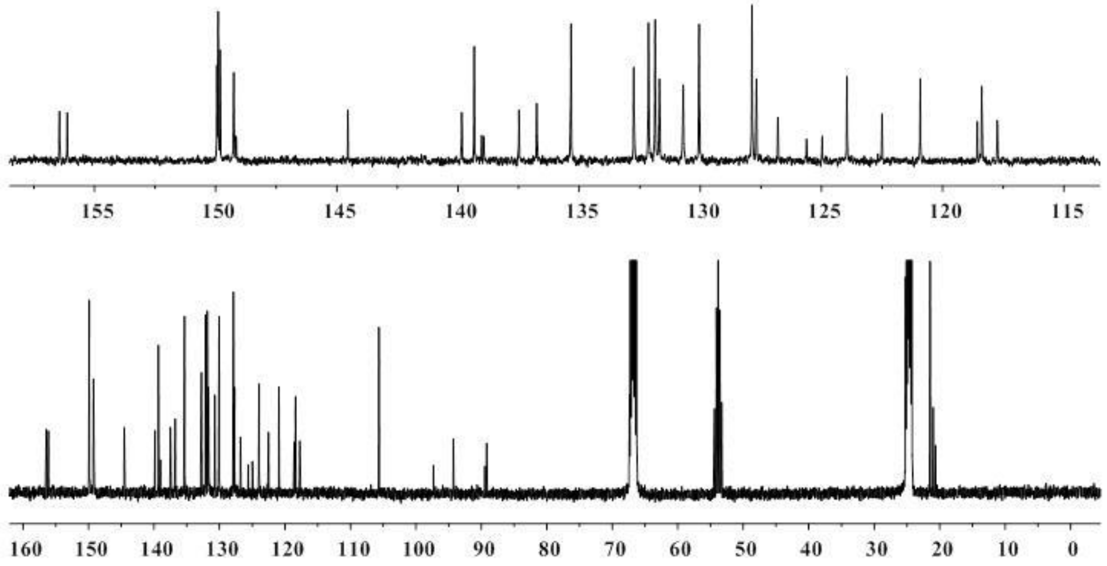

Figure S9. ^{13}C NMR spectrum of stator **B** in THF-d_8:CD_2Cl_2 (4:1) (100 MHz, 298 K).

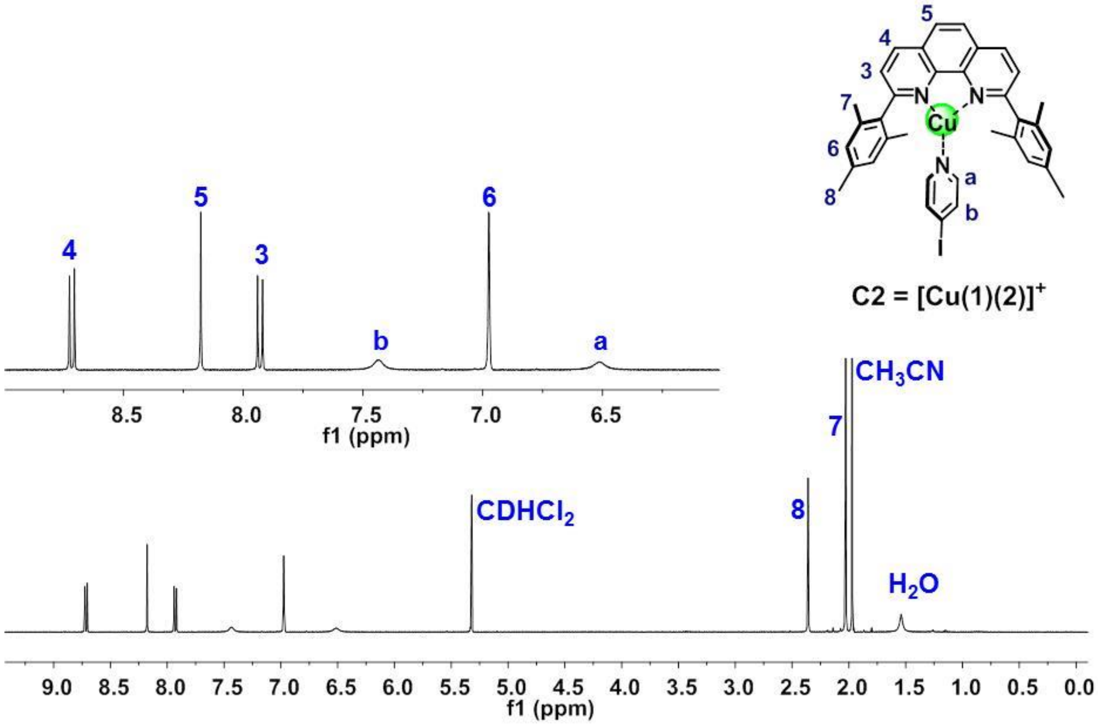

Figure S10. ^{1}H NMR spectrum of $[Cu(\mathbf{1})(\mathbf{2})]^+$ in CD_2Cl_2 (400 MHz, 298 K).

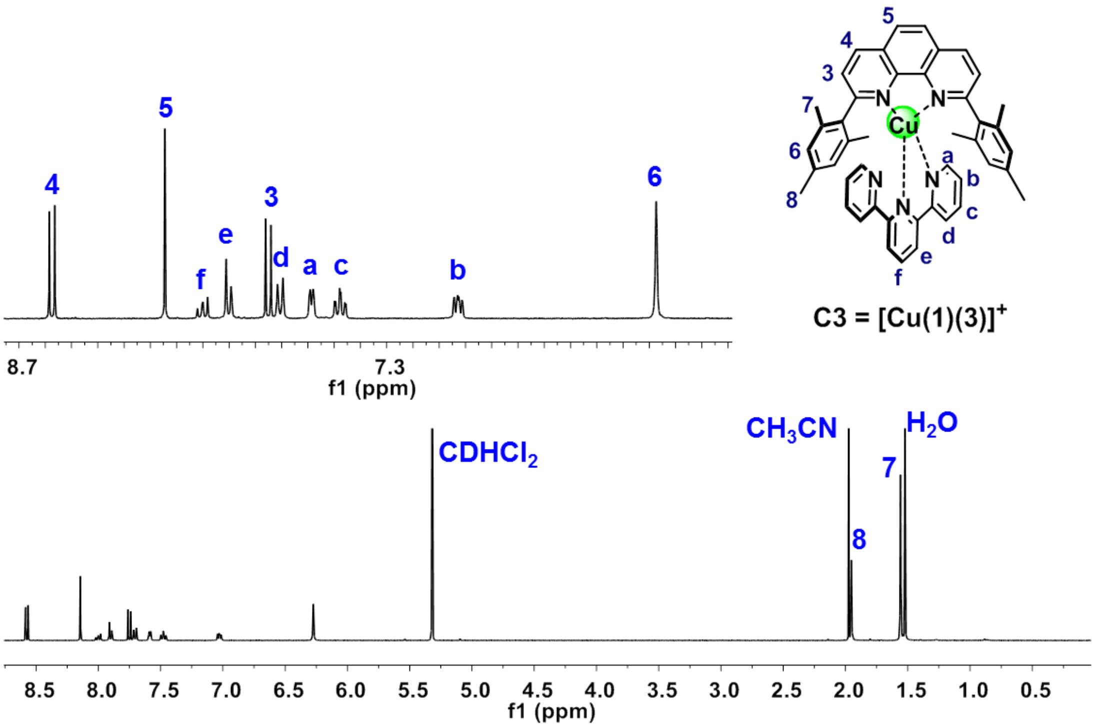

Figure S11. ^{1}H NMR spectrum of [Cu(**1**)(**3**)]$^+$ in CD_2Cl_2 (500 MHz, 298 K).

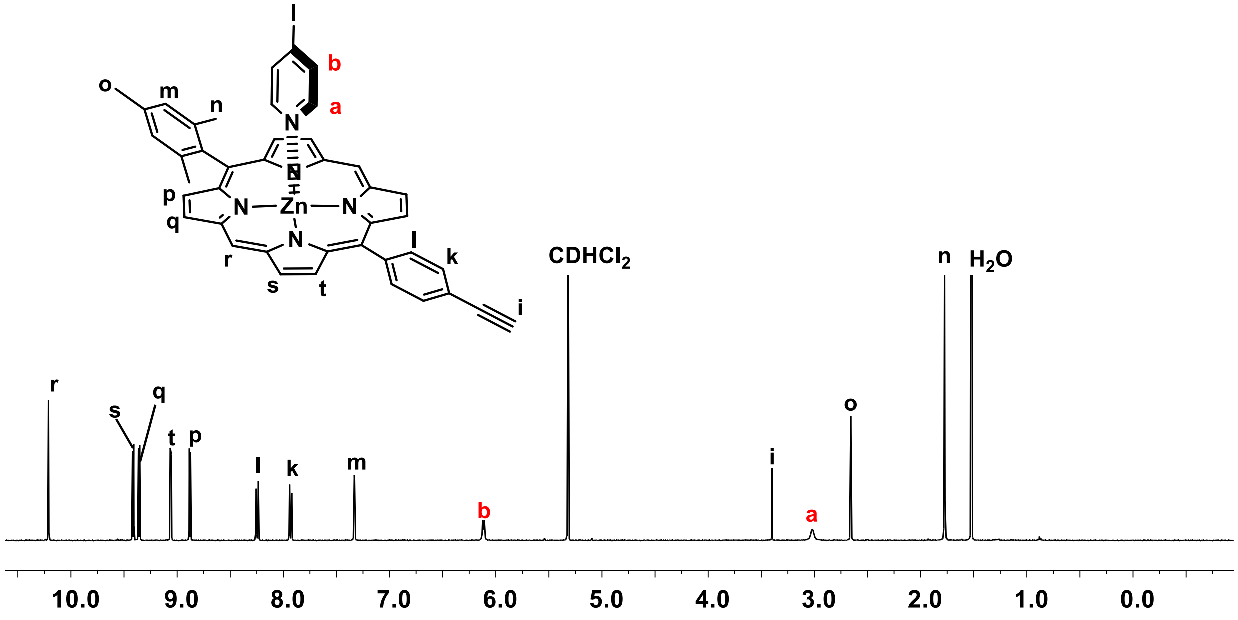

Figure S12. ^{1}H NMR spectrum of [(**2**)•(**4**)] in CD_2Cl_2 (400 MHz, 298 K).

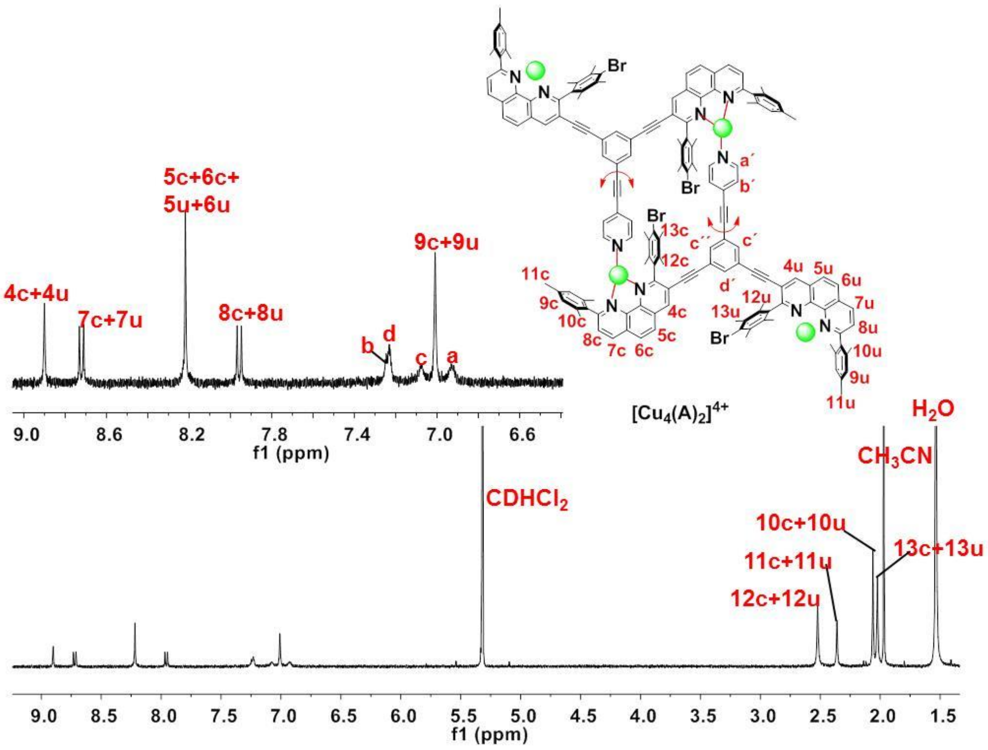

Figure S13. ^{1}H NMR spectrum of $[Cu_4(\mathbf{A})_2]^{4+}$ in CD_2Cl_2 (400 MHz, 298 K).

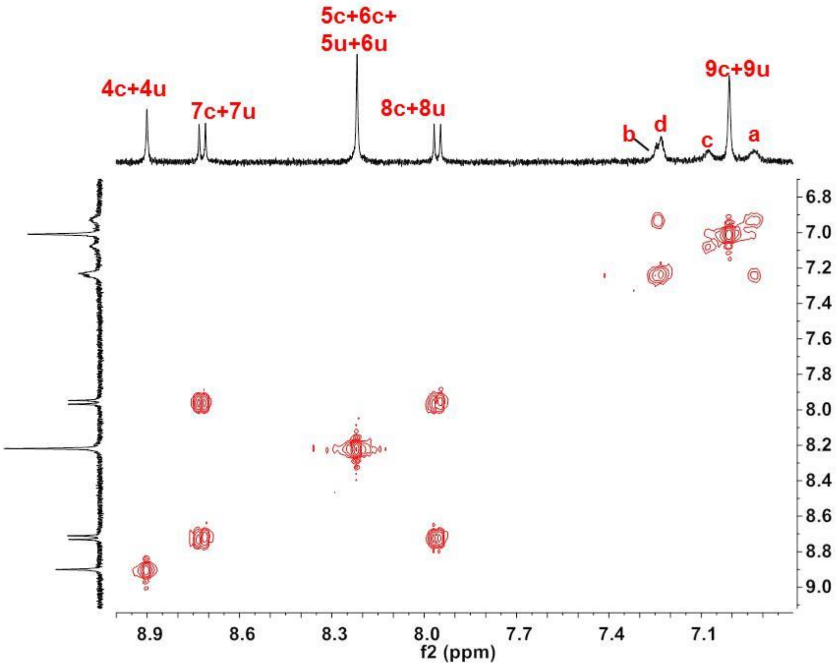

Figure S14. ^{1}H-^{1}H COSY spectrum of $[Cu_4(\mathbf{A})_2]^{4+}$ in CD_2Cl_2 (400 MHz, 298 K).

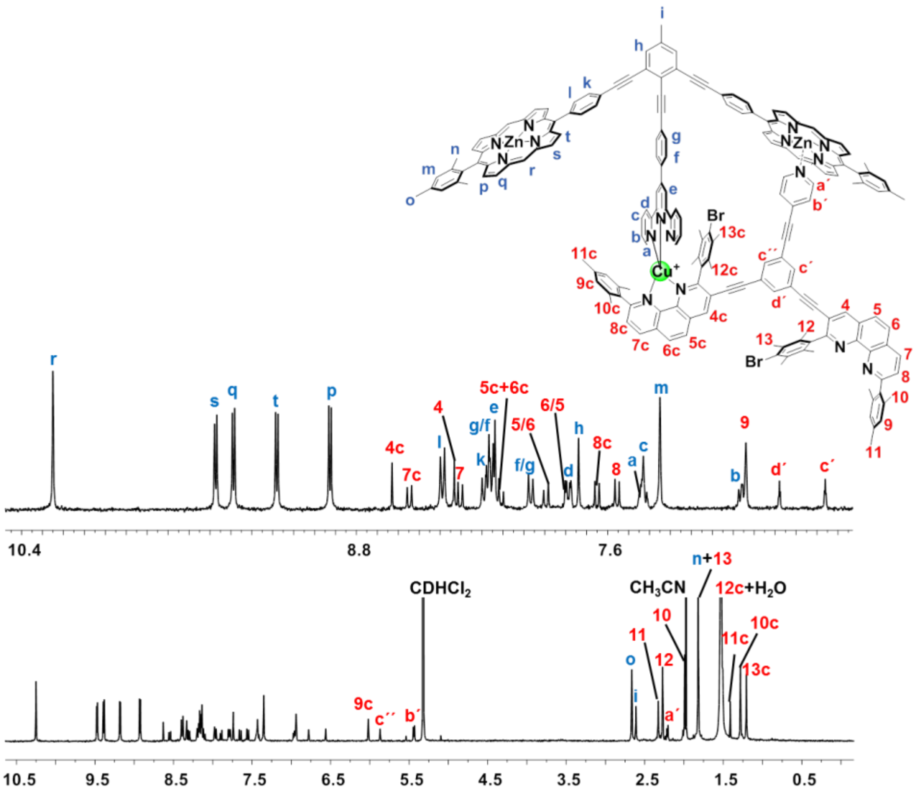

Figure S15. ^{1}H NMR spectrum of [Cu(**A**)(**B**)]$^+$ in CD_2Cl_2 (600 MHz, 298 K).

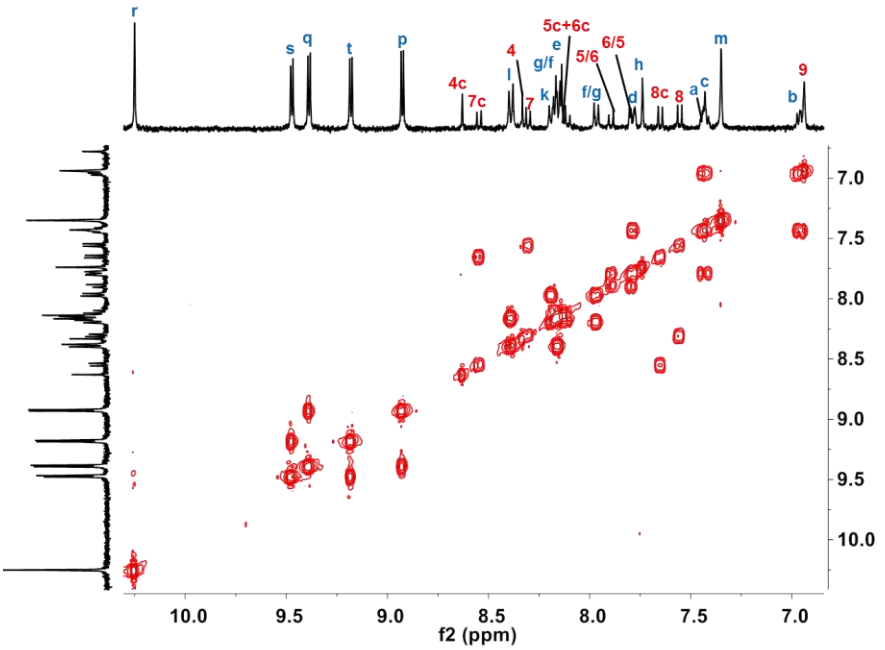

Figure S16. ^{1}H-^{1}H COSY spectrum of [Cu(**A**)(**B**)]$^+$ in CD_2Cl_2 (600 MHz, 298 K)

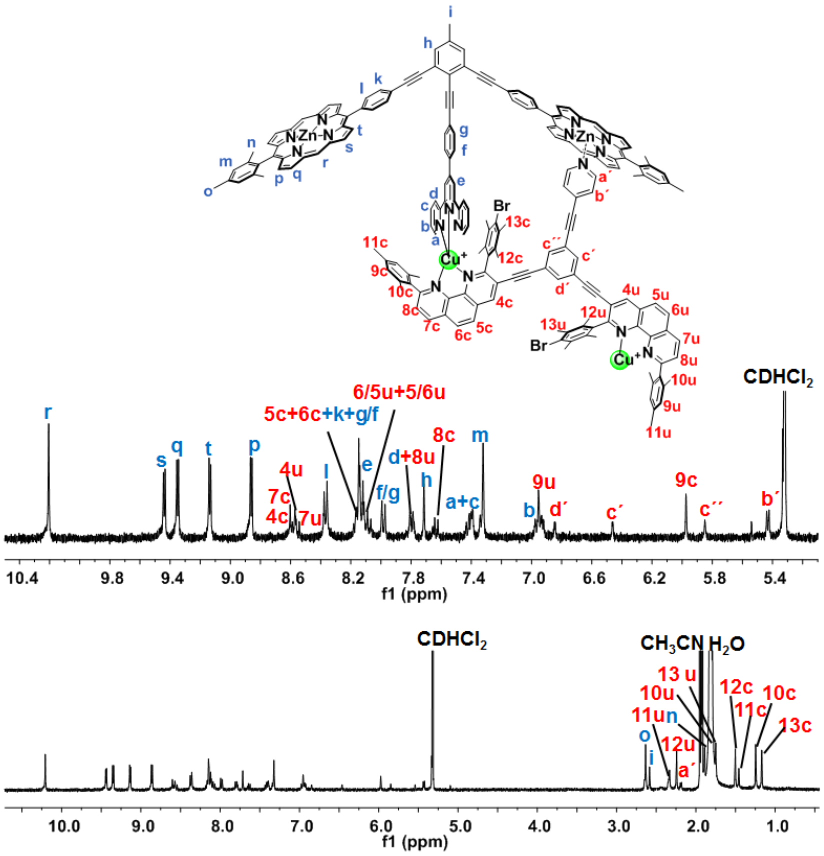

Figure S17. ^{1}H NMR spectrum of $[Cu_2(\mathbf{A})(\mathbf{B})]^{2+}$ in CD_2Cl_2:CD_3CN (5:1) (600 MHz, 298 K).

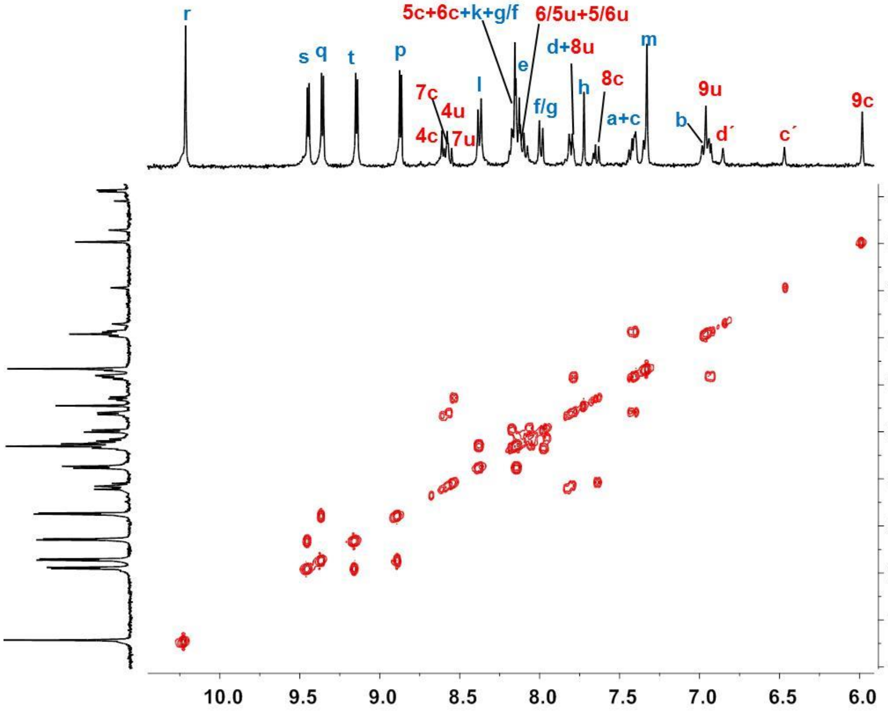

Figure S18. ^{1}H-^{1}H COSY spectrum of $[Cu_2(\mathbf{A})(\mathbf{B})]^{2+}$ in CD_2Cl_2:CD_3CN(5:1)(600 MHz, 298 K).

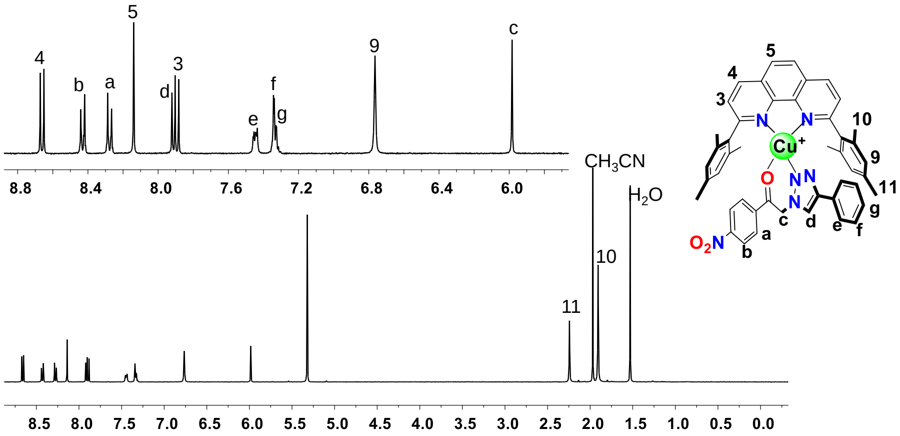

Figure S19. ^{1}H NMR spectrum of $[Cu(\mathbf{1})(\mathbf{7})]^+$ in CD_2Cl_2 (400 MHz, 298 K).

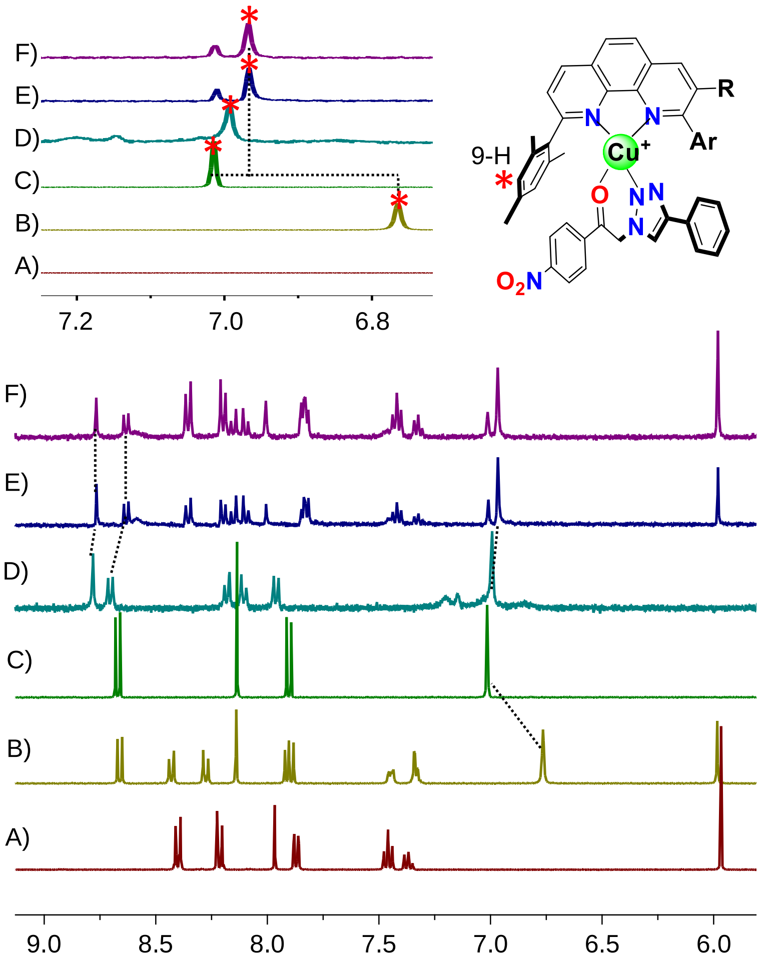

Figure S20. Chemical shift (Top: zoom spectra) of mesityl 9-H of phenanthroline in various setting. Partial ^{1}H NMR (400 MHz, 298 K) of (A) **7**, (B) $[Cu(\mathbf{1})(\mathbf{7})]^+$, (C) $[Cu(\mathbf{1})]^+$, (D) $[Cu_4(\mathbf{A})_2]^{4+}$, (E) $[Cu_4(\mathbf{A})_2]^{4+}$ + 2.0 equiv of **7** and (E) $[Cu_4(\mathbf{A})_2]^{4+}$ + 4.0 equiv of **7** in CD_2Cl_2:CD_3CN (5:1). Due to binding of ligand **7**, the static complex $[Cu(\mathbf{1})(\mathbf{7})]^+$ exhibits the proton signal of 9-H at 6.77 ppm as compared to $[Cu(\mathbf{1})]^+$ (7.02 ppm). The dynamic complex $(\mathbf{7})_2[Cu_4(\mathbf{A})_2]^{4+}$ shows the same signal at 6.97 ppm. Its reference, $[Cu_4(\mathbf{A})_2]^{4+}$, has the same proton signal located at 7.02 ppm. Thus, in $(\mathbf{7})_2[Cu_4(\mathbf{A})_2]^{4+}$ ligand **7** is expelled from the copper phenanthroline sites into solution.

4. DOSY NMR spectra

Calculation of hydrodynamic radius from:

a) **DOSY**: The diffusion coefficient D for $[Cu_4(\mathbf{A})_2]^{4+}$ and $[Cu_2(\mathbf{A})(\mathbf{B})]^{2+}$ was obtained from their DOSY spectrum. The corresponding hydrodynamic radius was calculated by using the Stokes-Einstein equation

$$r = k_B T / 6\pi\eta D$$

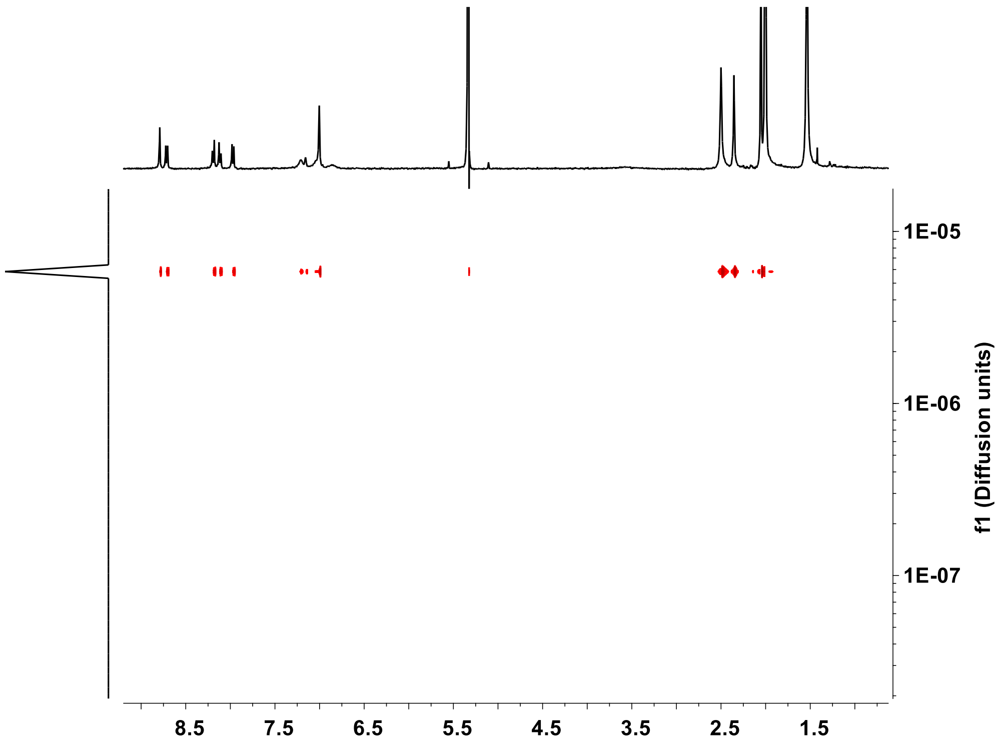

Figure S21. ^{1}H-DOSY NMR of $[Cu_4(\mathbf{A})_2]^{4+}$ in CD_2Cl_2 (600 MHz, 298 K). Diffusion coefficient $D = 4.1 \times 10^{-10}$ m^2 s^{-1}, hydrodynamic radius r = 12.9 Å.

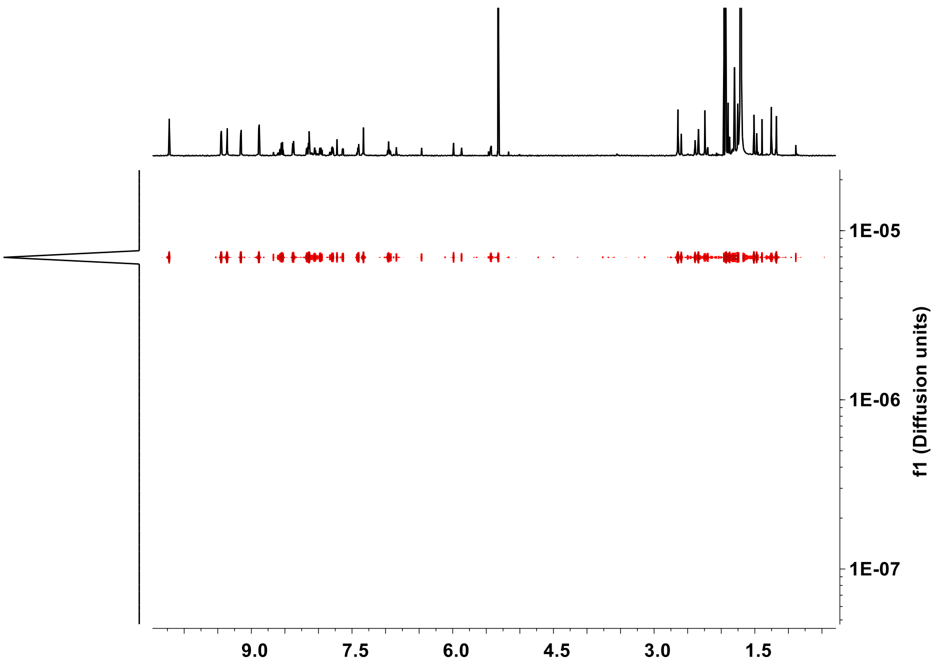

Figure S22. ^{1}H-DOSY NMR of $[Cu_2(\mathbf{A})(\mathbf{B})]^{2+}$ in CD_2Cl_2:CD_3CN (5:1) (600 MHz, 298 K). Diffusion coefficient $D = 4.8 \times 10^{-10}$ m^2 s^{-1}, hydrodynamic radius $r = 11.1$ Å.

5. Variable temperature studies, ROESY and determination of kinetic parameters

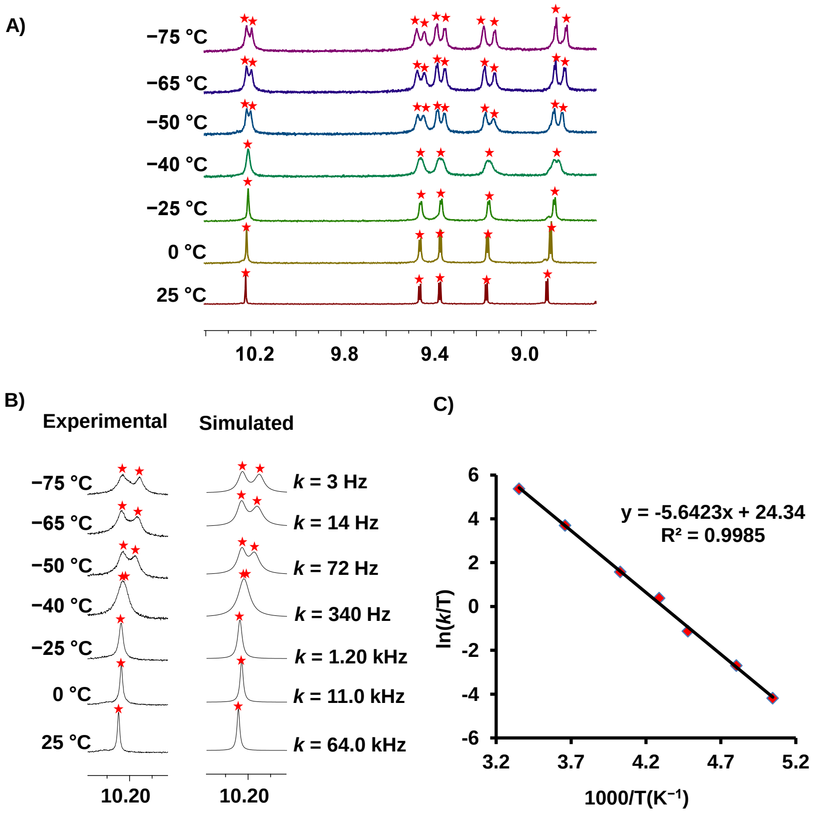

Figure S23. Partial ^{1}H VT-NMR spectra (CD_2Cl_2:CD_3CN (5:1), 600 MHz) of $[Cu_2(\mathbf{A})(\mathbf{B})]^{2+}$at different temperatures showing (A) experimental, and (B) theoretical splitting of r-H with corresponding rate constants. (C) Eyring plot for the rotational dynamics: $\Delta H^{‡}$ = 46.9 ± 0.4 kJ mol^{-1}, $\Delta S^{‡}$ = 4.8 ± 1.2 J mol^{-1} K^{-1}, $\Delta G^{‡}{}_{298}$ = 45.5 kJ mol^{-1}.

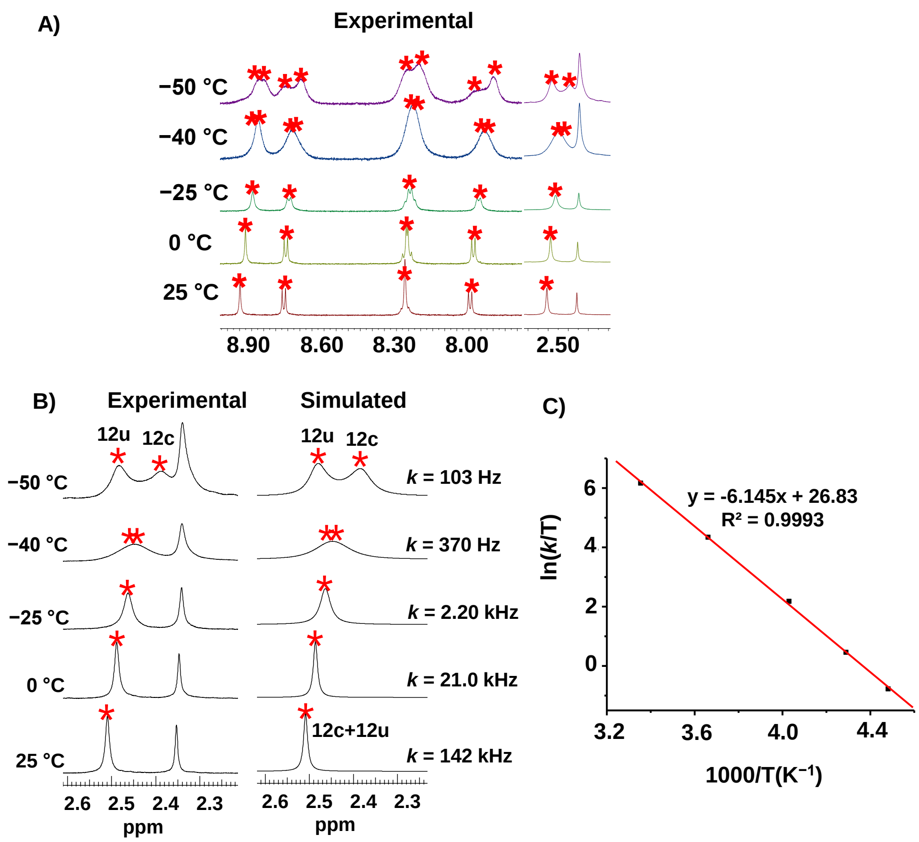

Figure S24. Partial ^{1}H VT-NMR (CD_2Cl_2, 600 MHz) of $[Cu_4(\mathbf{A})_2]^{4+}$ showing (A) experimental and (B) theoretical splitting of proton 12-H with corresponding rate constants. (C) Eyring plot for rotational dynamics in $[Cu_4(\mathbf{A})_2]^{4+}$: $\Delta H^{\ddagger} = 51.1 \pm 0.7$ kJ mol^{-1}, $\Delta S^{\ddagger} = 25.5 \pm 3.0$ J mol^{-1} K^{-1}, $\Delta G^{\ddagger}_{298} = 43.5$ kJ mol^{-1}.

Rate constant calculation by volume intensity of the ROESY cross peaks

$I_D/I_C = (1 - kt_M)/kt_M$ or $k = 1/[t_M(I_D/I_C + 1)]$

Where I_D : intensity of diagonal peak

I_C : intensity of cross peak

t_M: mixing time (0.3 s)

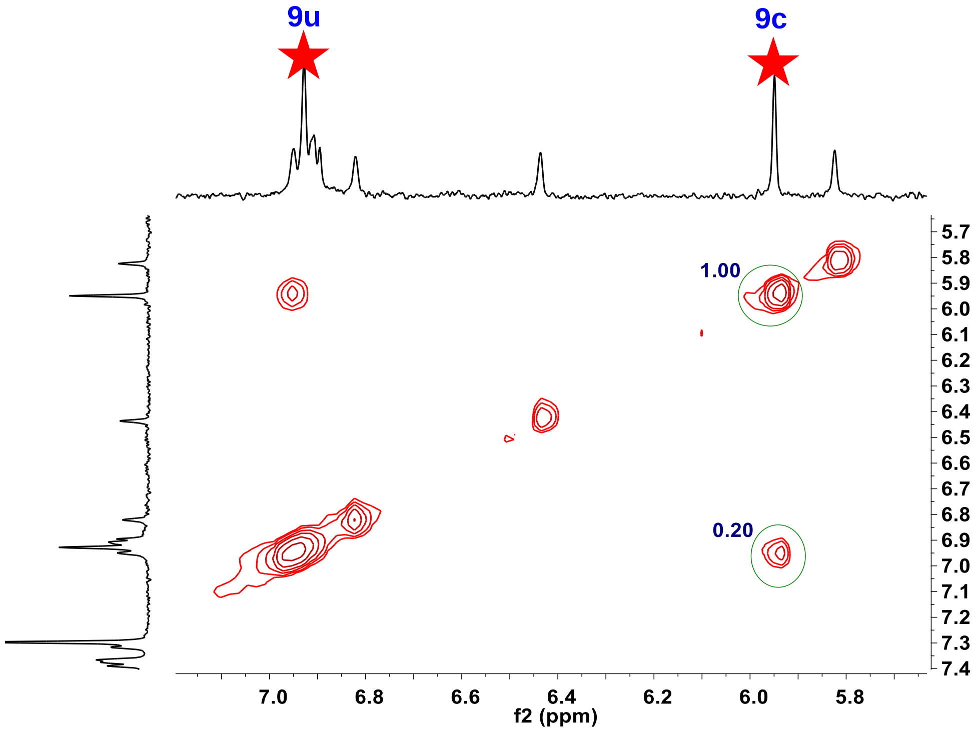

Figure S25. Partial ^{1}H-^{1}H ROESY (600 MHz, CD_2Cl_2:CD_3CN (5:1)) of $[Cu_2(\mathbf{A})(\mathbf{B})]^{2+}$ with volume intensity of cross and diagonal peaks.

Eyring Equation; $k = (k_B T/h)\exp(-\Delta G^{\neq}/RT)$

k_B: Boltzmann constant

h: Planck's constant

$k_{298} = 0.55\ s^{-1}$

$\Delta G^{\neq}_{298} = 74.8\ kJ\ mol^{-1}$

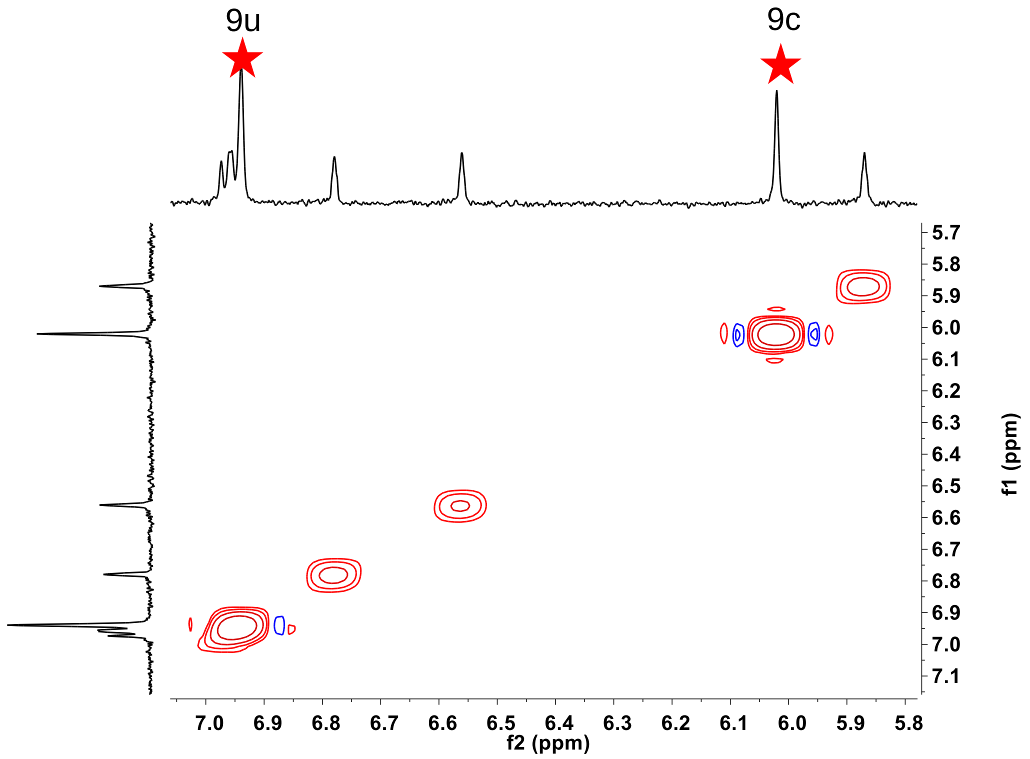

Figure S26. Partial ^{1}H-^{1}H ROESY (600 MHz, CD_2Cl_2:CD_3CN (5:1)) of [Cu(**A**)(**B**)]$^+$ shows no cross correlation between 9c and 9u-H

6. Catalytic experiments

6.1. Characterization of click product 7

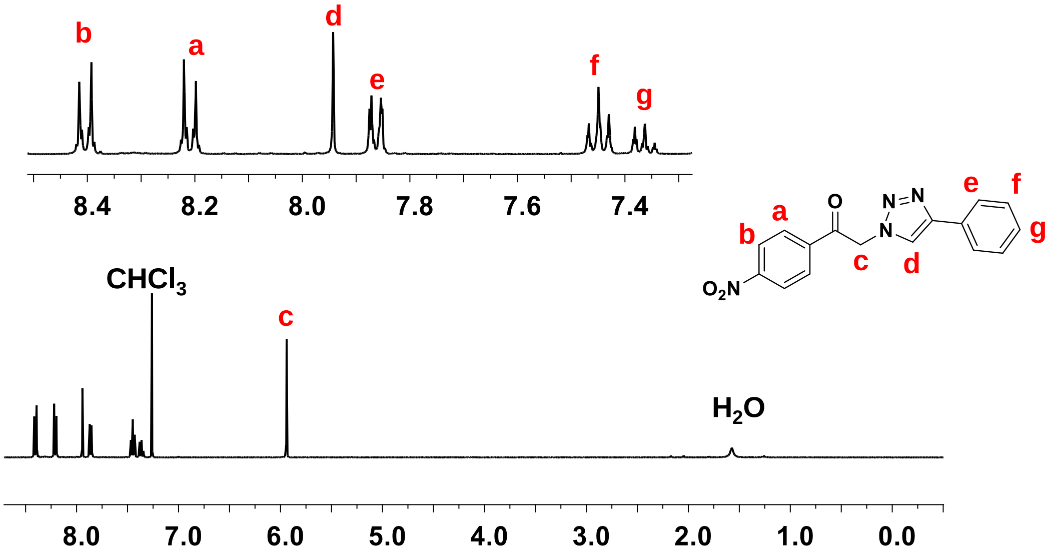

Figure S27. ^{1}H NMR (400 MHz, 298 K) spectrum of the click product **7** in $CDCl_3$.

6.2. General procedure

Solid reactants were transferred to the NMR tube and dissolved in CD_2Cl_2:CD_3CN (5:1). The mixture was heated at 50 °C for 2 h and the yield of the click product **7** (singlet at δ 5.98 ppm) was determined using 1,3,5-trimethoxybenzene (**12**) as an internal standard (singlet at δ 6.04 ppm).

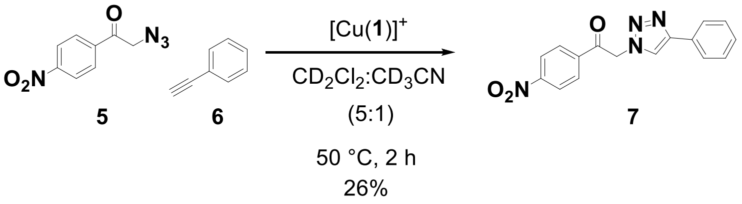

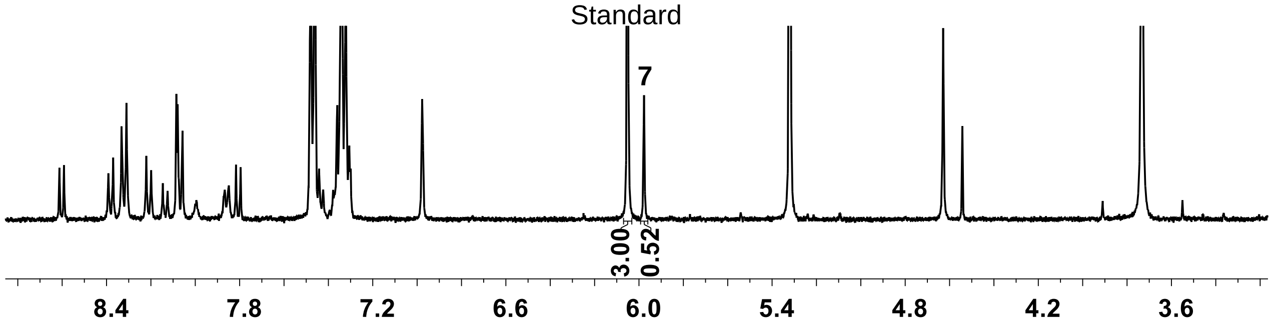

Figure S28. ^{1}H NMR (500 MHz, CD_2Cl_2:CD_3CN = 5:1, 298 K) spectrum obtained after heating the reaction mixture of compounds **5**, **6**, **1**, $[Cu(CH_3CN)_4]PF_6$ (≈ 3.6 mM) and standard **12** in 5:5:1:1:5 ratio at 50 °C for 2 h. Formation of 26% of product **7** was observed in ^{1}H NMR.

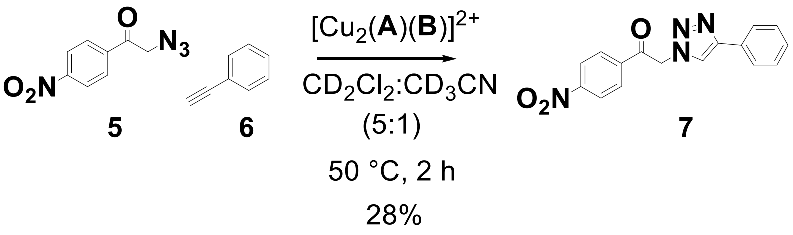

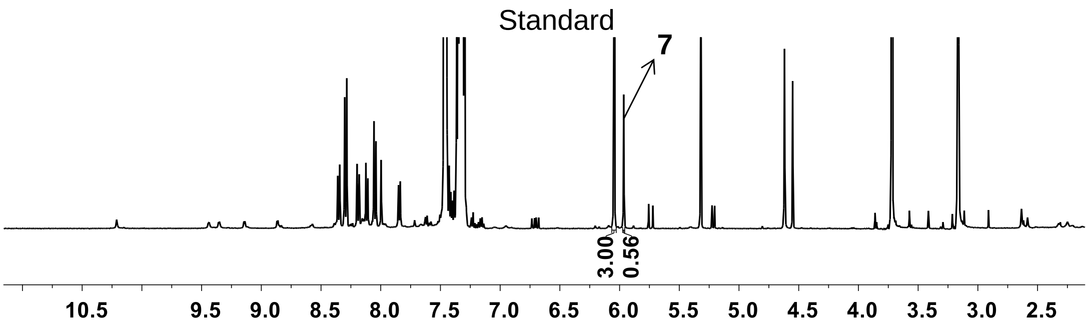

Figure S29. ^{1}H NMR (500 MHz, CD_2Cl_2:CD_3CN = 5:1, 298 K) spectrum obtained after heating the reaction mixture of compounds **5**, **6**, **A**, **B**, $[Cu(CH_3CN)_4]PF_6$ (≈ 3.6 mM) and standard **12** in 10:10:1:1:2:10 ratio at 50 °C for 2 h. The integration demonstrated that **7** were formed in 28% yield.

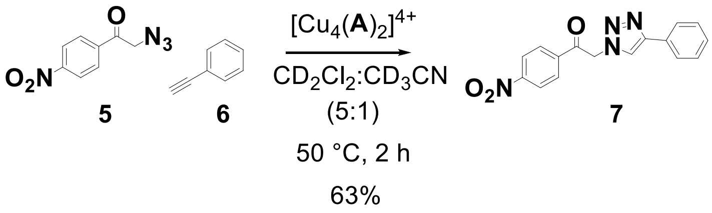

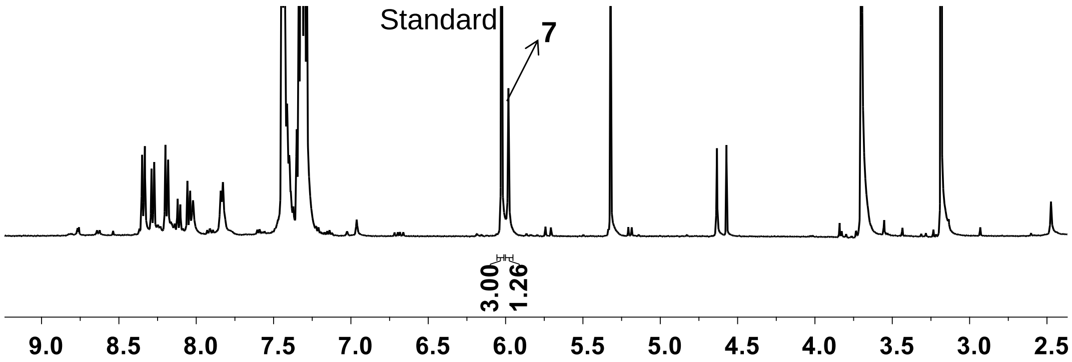

Figure S30. ^{1}H NMR (500 MHz, CD_2Cl_2:CD_3CN = 5:1, 298 K) spectrum obtained after heating the reaction mixture of compounds **5**, **6**, **A**, $[Cu(CH_3CN)_4]PF_6$ (≈ 3.6 mM) and standard **12** in 10:10:1:2:10 ratio at 50 °C for 2 h. 63% product **7** was observed in ^{1}H NMR.

Reduction of yield by adding deliberate amounts of product prior to the reaction.

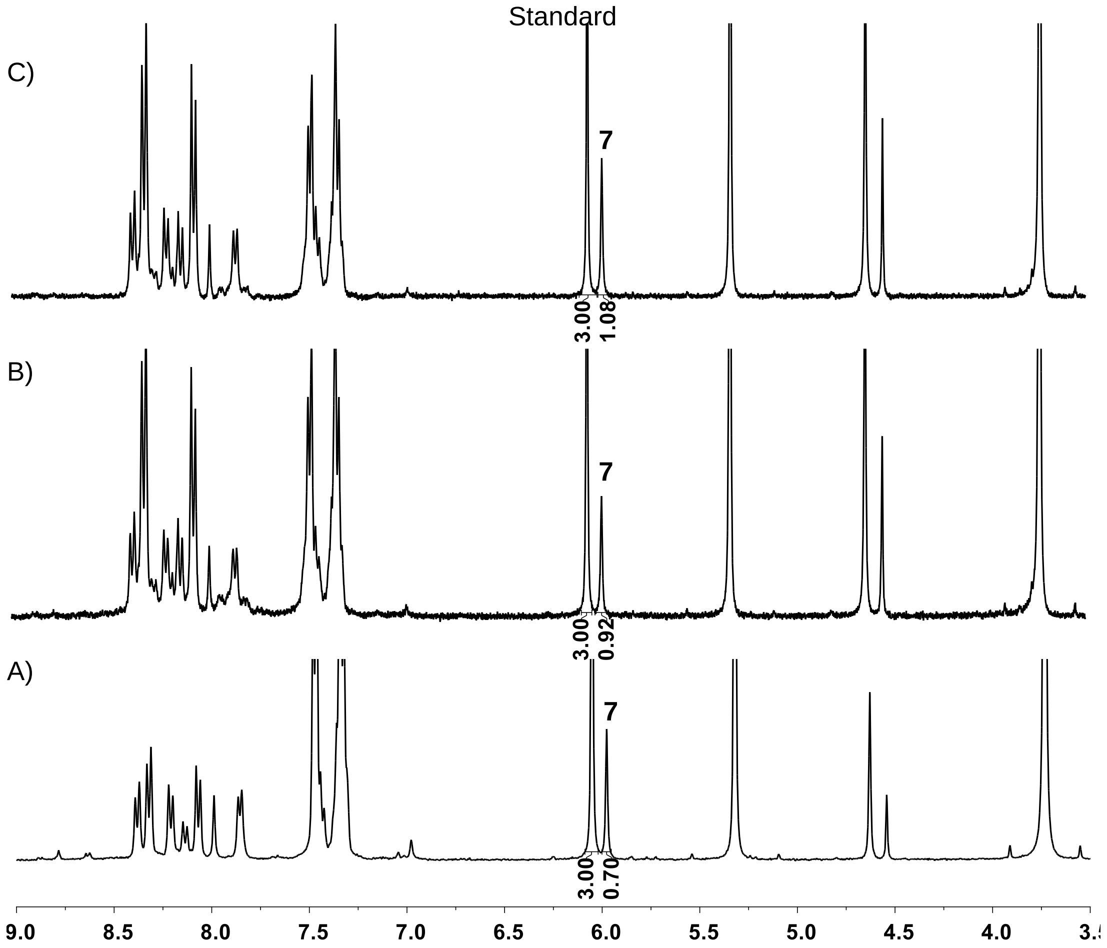

Figure S31. ^{1}H NMR (400 MHz, CD_2Cl_2:CD_3CN = 5:1, 298 K) spectrum obtained (A) after heating the reaction mixture of compounds **5**, **6**, **A**, $[Cu(CH_3CN)_4]PF_6$ (≈ 3.6 mM) and standard **12** in 10:10:1:2:10 ratio at 50 °C for 30 min. Yield of **7** is 35%. (B) After addition of the product **7** (3.6 mM, 20 mol% with respect to standard **12**) followed by heating at 50 °C for 30 min of another mixture as in A. Formation of 26% (46%−20%) of product **7** was observed. (C) After addition of the product **7** (7.2 mM 40 mol% with respect to standard **12**) followed by heating at 50 °C for 30 min of another mixture as in A. Formation of 14% (54%−40%) of product **7** was observed.

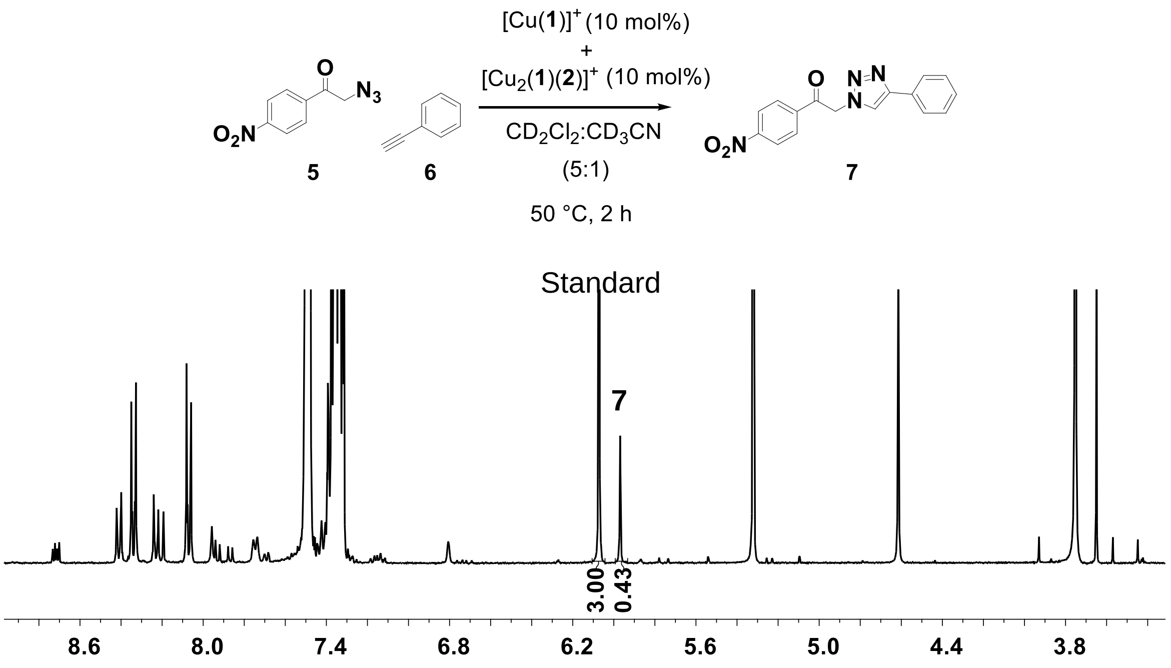

Figure S32. ^{1}H NMR (500 MHz, CD_2Cl_2:CD_3CN = 5:1, 298 K) spectrum obtained after heating the reaction mixture of compounds **5**, **6**, **1**, **2**, [Cu(CH_3CN)$_4$]PF_6 (≈ 3.6 mM) and standard **12** in 10:10:2:1:2:10 ratio at 50 °C for 2 h. Formation of 22% of product **7** was observed in ^{1}H NMR. Conclusion: pyridine does not accelerate the reaction.

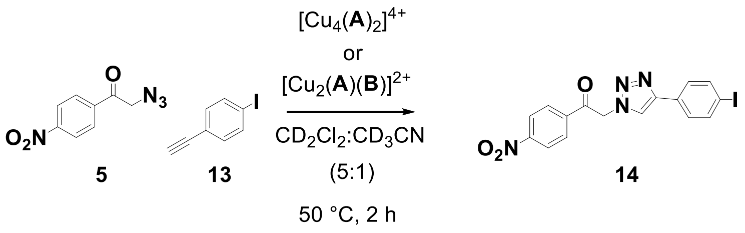

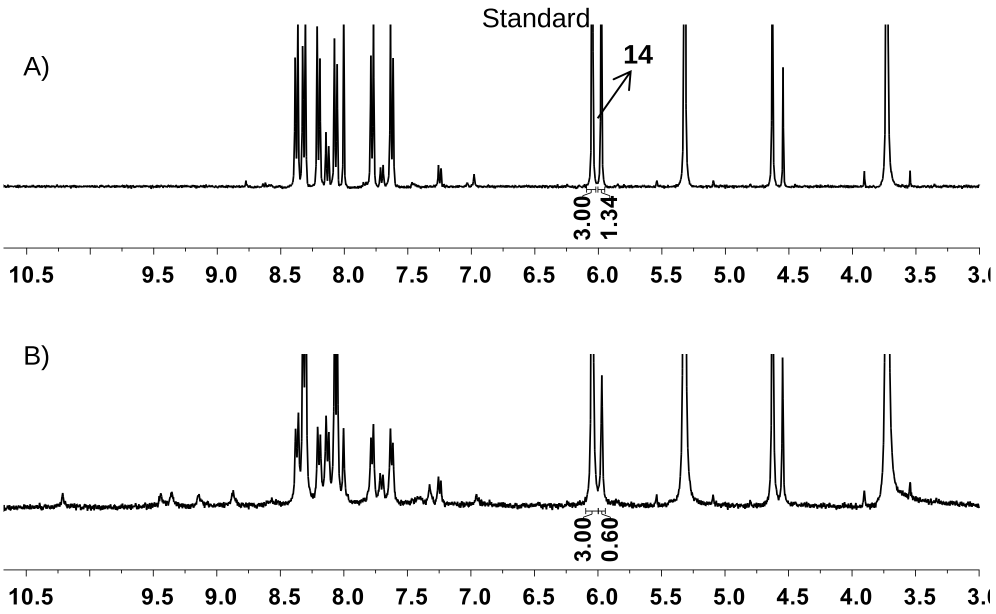

Figure S33. ^{1}H NMR (400 MHz, CD_2Cl_2:CD_3CN = 5:1, 298 K) spectrum obtained (A) after heating the reaction mixture of compounds **5**, **13**, **A**, [Cu(CH_3CN)$_4$]PF_6 (≈ 3.6 mM) and standard **12** in 10:10:1:2:10 ratio at 50 °C for 2 h. Yield of product **14** was 67%. (B) After heating the reaction mixture of compounds **5**, **13**, **A**, **B**, [Cu(CH_3CN)$_4$]PF_6 (≈ 3.6 mM) and standard **12** in 10:10:1:1:2:10 ratio at 50 °C for 2 h. The integration demonstrated that **14** was formed in 30% yield.

7. ESI-MS spectra

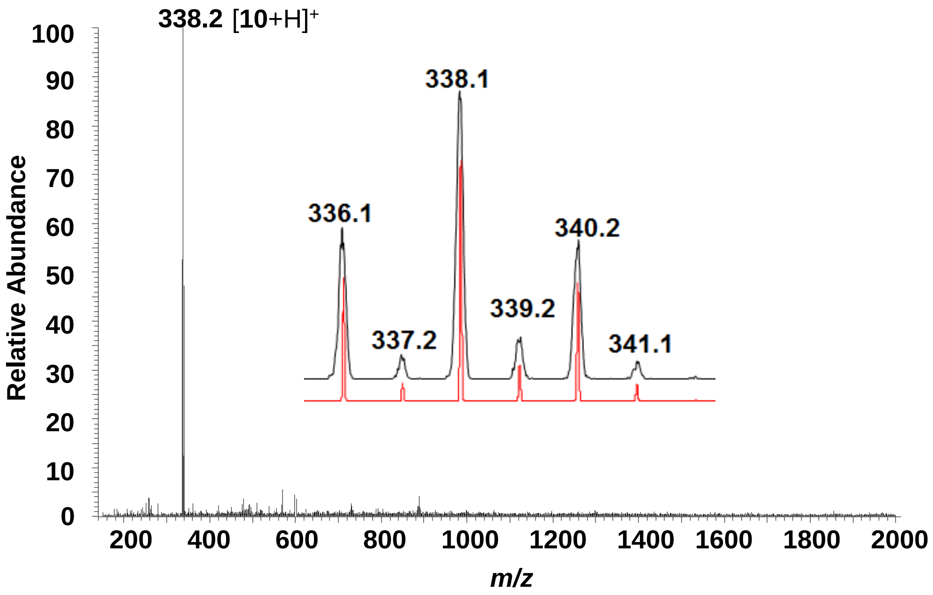

Figure S34. ESI-MS of compound **10** after protonation.

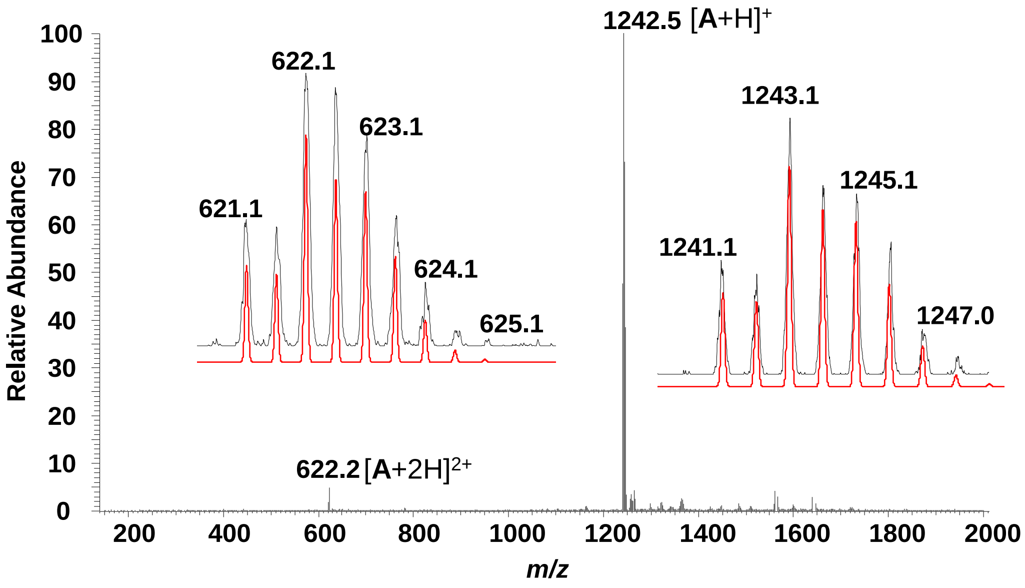

Figure S35. ESI-MS of **A** after protonation.

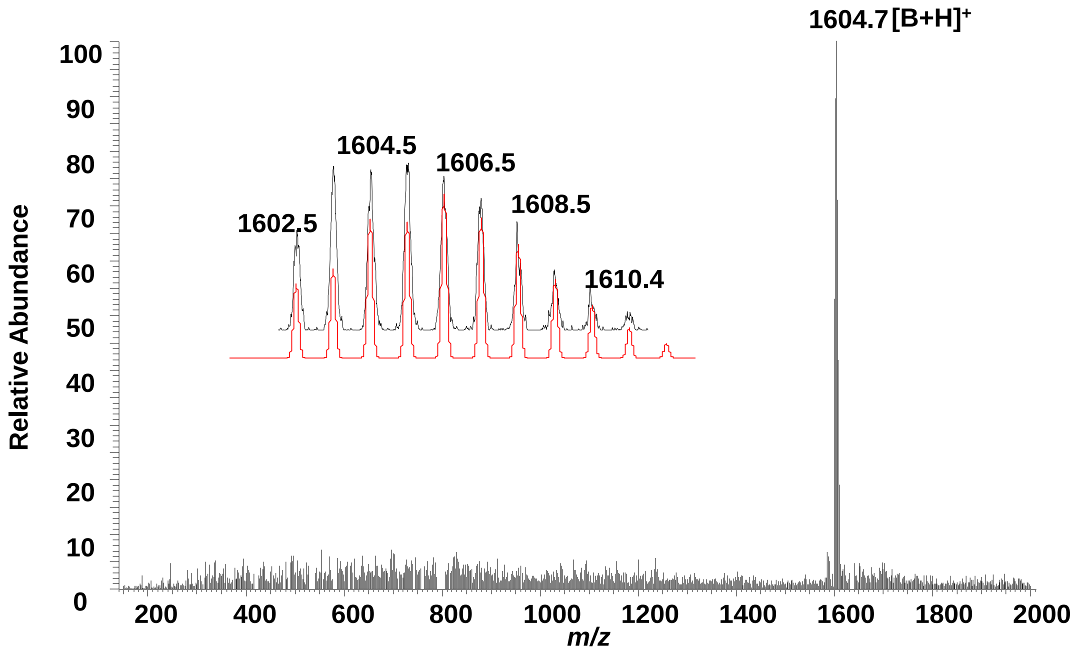

Figure S36. ESI-MS of compound **B** after protonation.

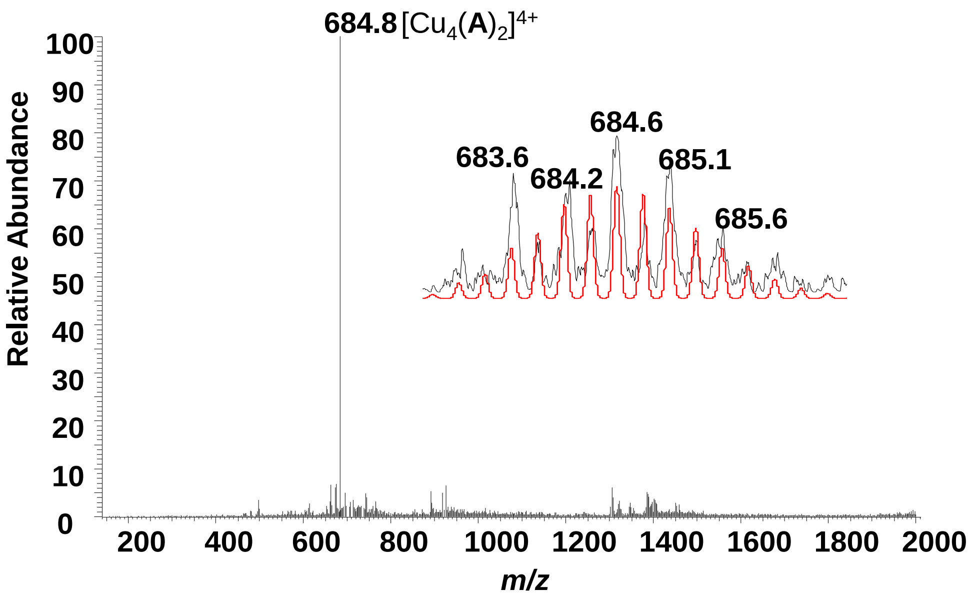

Figure S37. ESI-MS of $[Cu_4(\mathbf{A})_2]^{4+}$.

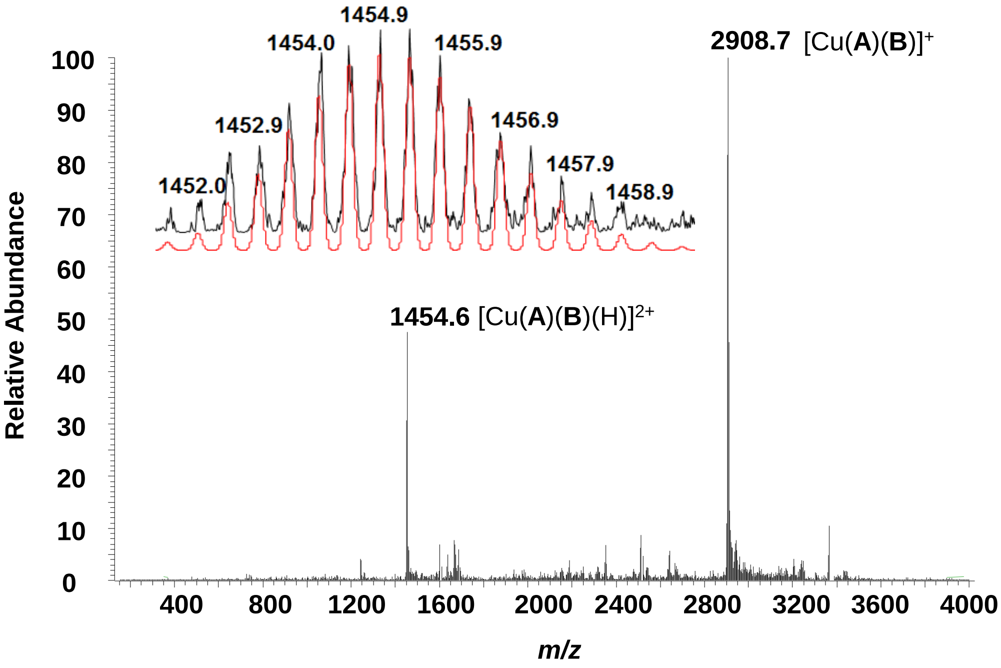

Figure S38. ESI-MS of [Cu(**A**)(**B**)]$^{+}$.

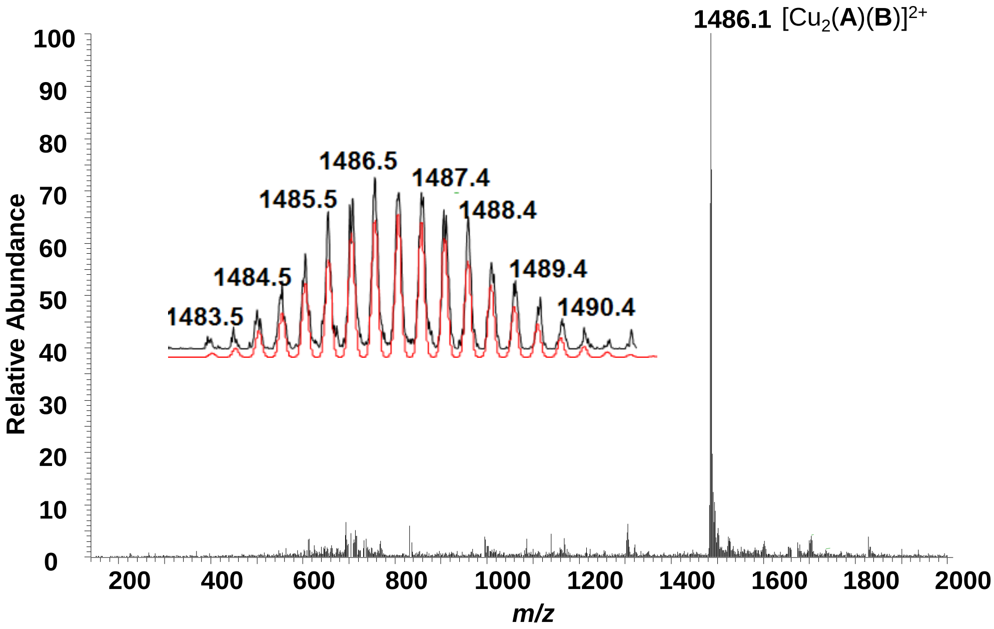

Figure S39. ESI-MS of [Cu$_2$(**A**)(**B**)]$^{2+}$.

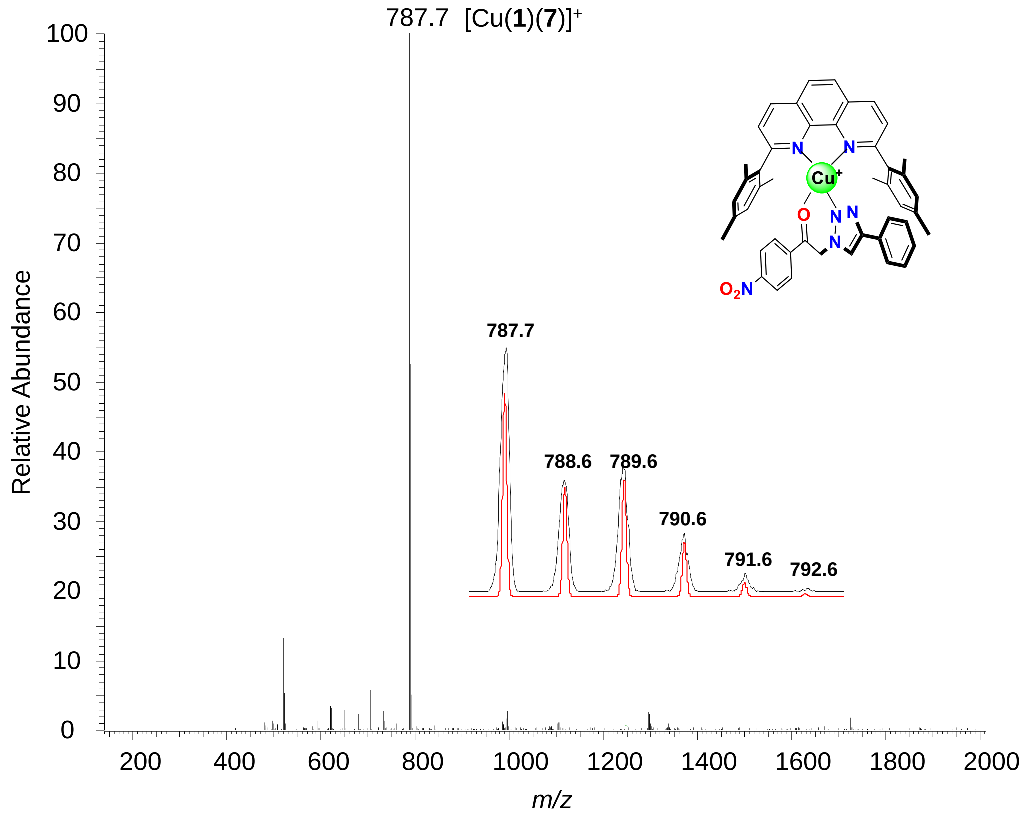

Figure S40. ESI-MS spectrum of [Cu(**1**)(**7**)]$^+$ in CD_2Cl_2.

8. Elucidation of v_0 for the reactions using different catalyst

A mixture of substrate **5** (18.0 mM), **6** (18.0 mM), standard **12** (18.0 mM), catalyst $[Cu_4(\mathbf{A})_2]^{4+}$ (0.9 mM), (or $[Cu_2(\mathbf{A})(\mathbf{B})]^{2+}$ (1.8 mM) or $[Cu(\mathbf{1})]^{+}$ (3.6 mM)) was taken in an NMR tube and heated at 50 °C after dissolving in CD_2Cl_2:CD_3CN (5:1). ^{1}H NMR (400 MHz, 298 K) spectra were recorded to follow the formation of product **7** with time. The results are depicted in the publication in Figure 5A. With the formed product given in mol L^{-1} the reaction rate v_0 was determined as the slope between time zero and 2 min:

$v_0 = 4.2 \times 10^{-6}$ mol L^{-1} s^{-1} (catalyst: $[Cu_4(\mathbf{A})_2]^{4+}$)

$v_0 = 1.8 \times 10^{-6}$ mol L^{-1} s^{-1} (catalyst: $[Cu_2(\mathbf{A})(\mathbf{B})]^{2+}$)

$v_0 = 1.4 \times 10^{-6}$ mol L^{-1} s^{-1} (catalyst: $[Cu(\mathbf{1})]^{+}$)

9. UV-Vis data

Measurement of thermodynamic constants

A UV-vis titration was used to measure the thermodynamic driving force of the complexation of $[Cu_4(\mathbf{A})_2]^{4+}$ + 2 × **B**.

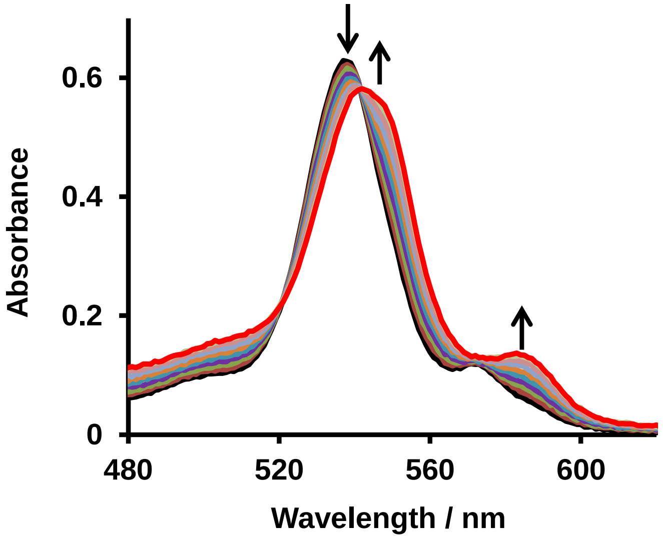

Figure S41. UV-vis titration of complex **B** (1.69×10^{-5} M) vs. $[Cu_4(\mathbf{A})_2]^{4+}$ (1.03×10^{-3} M) in CH_2Cl_2 at 298 K. Overall "equilibrium" constant was determined to be log $K = 5.98 \pm 0.51$ using SPECFIT software.

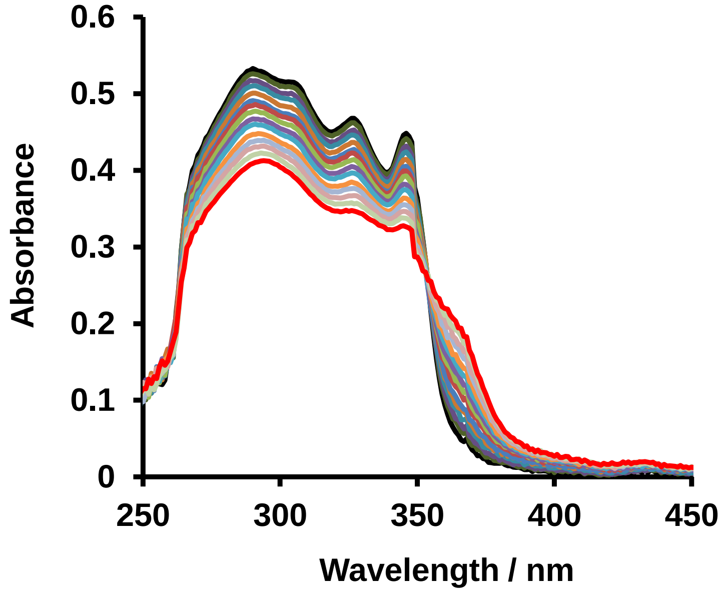

Figure S42. UV-vis titration of **A** (4.8×10^{-5} M) vs. $[Cu(CH_3CN)_4]PF_6$ (2.4×10^{-3} M) in CH_2Cl_2 at 298 K. Binding constant was determined to be log β = 12.24 ± 0.16 using SPECFIT software.

Kinetic investigation

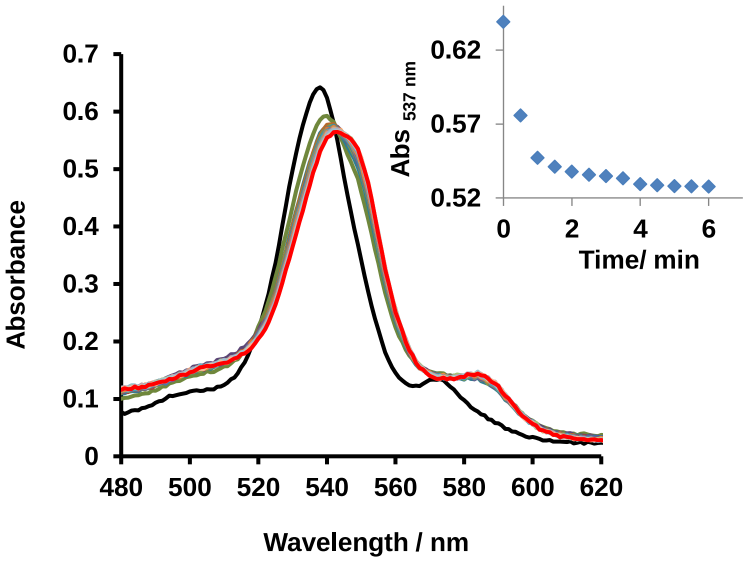

Figure S43. UV−vis spectra of the reaction between **B** and [Cu_4(**A**)$_2$]$^{4+}$ (1.2×10^{-5} M) at 298 K in CH_2Cl_2 indicating formation of [Cu_2(**A**)(**B**)]$^{2+}$ with time. Inset: Change of absorbance at λ = 537 nm with time. Half-life was determined as $t_{1/2}$ = 30 sec.

10. Computational data.

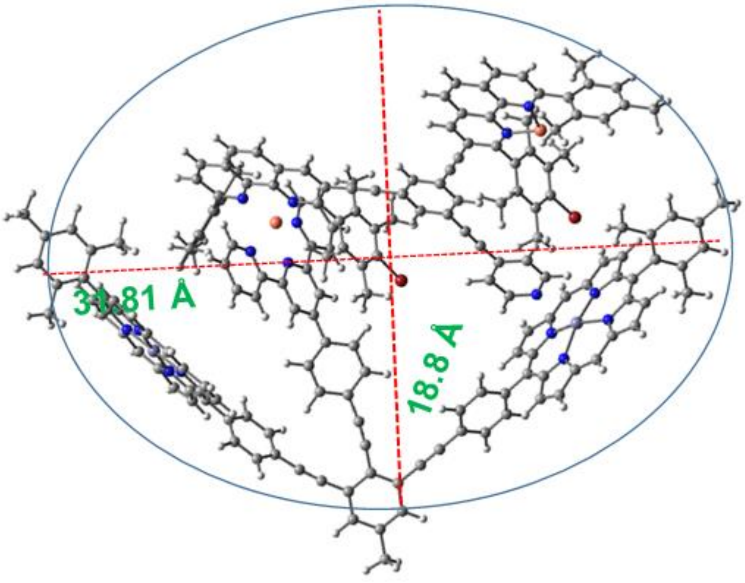

Figure S44. Hydrodynamic radius of [Cu_2(**A**)(**B**)]$^{2+}$ from computed structure calculated using PM6 level. Radius r_{com} = (31.8+18.8)/4 = 12.7 Å. Experimentally found radius r_{exp} = 11.1 Å.

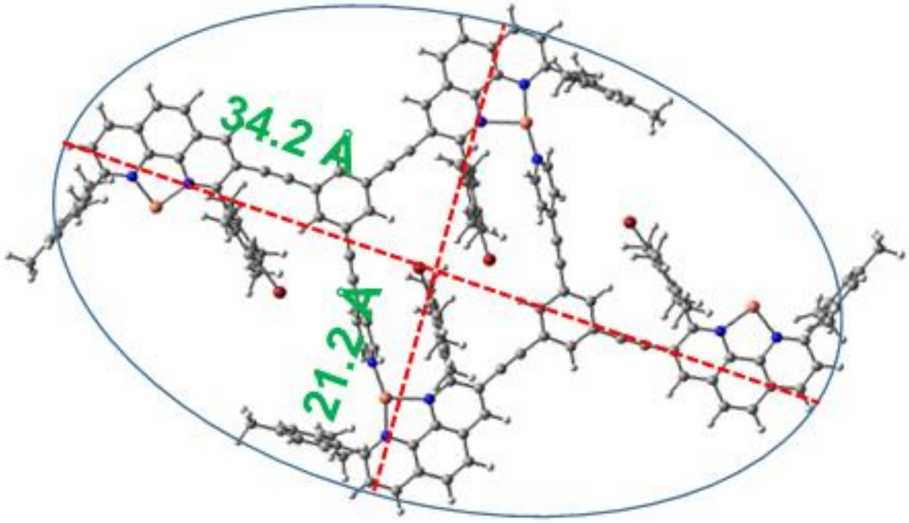

Figure S45. Hydrodynamic radius of [Cu_4(**A**)]$^{4+}$ from computed structure calculated using PM6 level. Radius r_{com} = (34.2+21.2)/4 = 13.9 Å. Experimentally found radius r_{exp} = 12.9 Å.

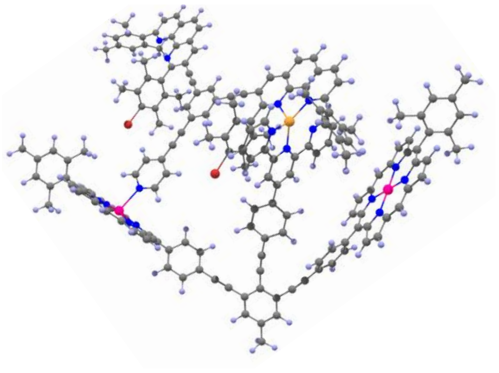

Figure S46. PM6-optimized structure of rotor [Cu(**A**)(**B**)]$^{+}$.

C	-4.032500000	10.403400000	-3.152400000
C	-4.433500000	11.621300000	-3.726300000
C	-6.651700000	10.716900000	-4.147400000
C	-6.268700000	9.498900000	-3.574500000
C	-4.950700000	9.330300000	-3.066600000
H	-3.720300000	12.444100000	-3.786100000
H	-7.664200000	10.836100000	-4.532500000
C	-4.594100000	8.084600000	-2.503700000
C	-4.375200000	6.981700000	-2.054200000
C	-5.734300000	11.777400000	-4.221600000
C	-4.282700000	5.691700000	-1.484400000
C	-5.340000000	4.780000000	-1.691500000
C	-3.163400000	5.300400000	-0.726800000
C	-5.282000000	3.506500000	-1.133500000
H	-6.204900000	5.082100000	-2.288500000
C	-3.114100000	4.025300000	-0.164900000
H	-2.340700000	6.000900000	-0.578600000
C	-4.171200000	3.122200000	-0.360400000
H	-6.109000000	2.812500000	-1.289500000
H	-2.241700000	3.727600000	0.419800000
C	-4.145800000	1.779700000	0.251100000
C	-3.812900000	1.607800000	1.606300000
C	-4.488800000	0.647000000	-0.497200000
C	-3.856900000	0.322100000	2.161700000
H	-3.536600000	2.473400000	2.212000000
C	-4.509400000	-0.622500000	0.116800000
H	-4.730700000	0.748000000	-1.557800000
C	-4.900700000	-1.827000000	-0.677600000
C	-5.203900000	-1.734300000	-2.049100000
C	-5.544500000	-2.896400000	-2.750100000
H	-5.178100000	-0.774300000	-2.567200000
C	-5.253500000	-4.138000000	-0.709800000
C	-5.573200000	-4.118300000	-2.079100000
H	-5.782800000	-2.841900000	-3.814800000
H	-5.260100000	-5.076700000	-0.141200000
H	-5.839900000	-5.038100000	-2.598300000
C	-3.562700000	0.087100000	3.604600000
C	-3.214500000	1.129900000	4.480700000
C	-3.465400000	-1.476400000	5.347300000
C	-2.989600000	0.835500000	5.829400000
H	-3.119300000	2.155100000	4.117600000
C	-3.122500000	-0.480500000	6.275800000
H	-3.598300000	-2.533100000	5.661800000
H	-2.717200000	1.631800000	6.526000000
H	-2.963000000	-0.734300000	7.323600000
N	-4.198100000	-0.797700000	1.435400000
N	-3.672500000	-1.218400000	4.026900000
N	-4.920200000	-3.023400000	-0.012300000
C	-2.670600000	-4.943500000	1.758100000
C	-1.112100000	-3.223400000	1.419900000
C	-1.783000000	-5.926900000	1.260200000
C	-3.989000000	-5.346200000	2.250700000
C	-0.141000000	-4.171100000	0.919100000
C	-0.492300000	-5.509300000	0.835000000
C	-2.201100000	-7.307600000	1.204800000
C	-4.350900000	-6.712000000	2.194000000
H	0.215900000	-6.246800000	0.447600000
C	-3.430900000	-7.681300000	1.648300000
H	-1.498800000	-8.038700000	0.798300000
C	-5.628200000	-7.082400000	2.703300000
C	-6.011800000	-4.751300000	3.285100000
H	-3.752600000	-8.724500000	1.608800000
H	-5.939000000	-8.129000000	2.663000000
C	-6.443900000	-6.119100000	3.244500000
H	-7.425600000	-6.366500000	3.653800000
N	-2.340500000	-3.594200000	1.819900000
N	-4.820700000	-4.358500000	2.780000000
C	1.133000000	-3.710200000	0.526600000
C	2.214000000	-3.261600000	0.218500000
C	3.459700000	-2.676800000	-0.118700000
C	4.501500000	-3.458800000	-0.645600000
C	3.635900000	-1.297100000	0.087100000
C	5.725800000	-2.847600000	-0.959900000
H	4.362700000	-4.529600000	-0.804100000
C	4.866900000	-0.698500000	-0.229800000
H	2.822900000	-0.695100000	0.495500000
C	5.917200000	-1.471800000	-0.756200000
H	6.872300000	-1.004500000	-1.002100000
C	5.039300000	0.692700000	-0.019100000
C	5.193400000	1.877000000	0.175100000
C	5.436100000	3.255700000	0.396300000
C	6.744600000	3.758400000	0.312400000
C	4.390600000	4.145700000	0.700200000
C	6.963400000	5.122900000	0.536600000
H	7.580400000	3.099300000	0.077700000
C	4.690800000	5.500200000	0.908200000
H	3.363600000	3.791200000	0.774000000
H	7.991600000	5.516800000	0.472300000
H	3.889100000	6.218200000	1.143400000
N	5.958800000	5.998800000	0.833200000
C	-2.714700000	10.249900000	-2.656400000
C	-1.586600000	10.098800000	-2.243800000
C	-0.263400000	9.892600000	-1.782400000
C	0.425400000	10.897300000	-1.076700000
C	0.366600000	8.658300000	-2.033900000
C	1.727700000	10.668700000	-0.632900000
H	-0.058100000	11.855500000	-0.888000000
C	1.665200000	8.434200000	-1.583300000
H	-0.164800000	7.883800000	-2.585800000
C	2.357500000	9.436000000	-0.879100000
H	2.269800000	11.450200000	-0.099700000
H	2.157200000	7.483400000	-1.781900000
C	3.735100000	9.173800000	-0.416500000
C	4.755500000	9.098100000	-1.390900000
C	3.989900000	9.038400000	0.951600000
N	6.063500000	8.785500000	-1.176300000
C	4.536600000	9.385200000	-2.844800000
N	5.192000000	8.716800000	1.532000000
C	2.954700000	9.222000000	2.023500000
Zn	6.961600000	8.221400000	0.586200000
C	6.740400000	8.887500000	-2.445500000
C	5.725000000	9.256500000	-3.470800000
H	3.576300000	9.651400000	-3.247800000

C	4.991900000	8.709300000	2.951200000
C	3.556000000	9.028300000	3.213800000
H	1.932100000	9.471800000	1.805500000
N	8.804400000	8.235100000	-0.334500000
N	7.899800000	8.114700000	2.418500000
C	8.060800000	8.704500000	-2.671600000
H	5.965600000	9.389100000	-4.509400000
C	5.924300000	8.461100000	3.899700000
H	3.144800000	9.084900000	4.204200000
C	9.049300000	8.392300000	-1.671800000
C	10.051400000	7.959400000	0.273200000
C	7.315600000	8.186800000	3.644200000
C	9.285700000	7.863500000	2.641200000
H	8.448000000	8.805400000	-3.692100000
H	5.636700000	8.475600000	4.954900000
C	10.460800000	8.215200000	-1.953800000
C	11.075300000	7.946400000	-0.751300000
C	10.265700000	7.792000000	1.655600000
C	8.302000000	7.967400000	4.699100000
C	9.506700000	7.766200000	4.079400000
H	10.906600000	8.291700000	-2.923200000
H	12.116600000	7.762100000	-0.564100000
C	11.663700000	7.534300000	2.095900000
H	8.081600000	7.974200000	5.746100000
H	10.466100000	7.575100000	4.524800000
C	12.134500000	6.213400000	2.232300000
C	12.519200000	8.620700000	2.377400000
C	13.450800000	5.984300000	2.651200000
C	11.231400000	5.062300000	1.927700000
C	13.832800000	8.376000000	2.795800000
C	12.022500000	10.019700000	2.229300000
C	14.301800000	7.062100000	2.935100000
H	13.815400000	4.966100000	2.755300000
H	10.320800000	5.101600000	2.546200000
H	10.911700000	5.084800000	0.875100000
H	11.705700000	4.089700000	2.106100000
H	14.493800000	9.211700000	3.012300000
H	11.682700000	10.214600000	1.198100000
H	11.154900000	10.207400000	2.882400000
H	12.780100000	10.775200000	2.469500000
C	15.709900000	6.814100000	3.368900000
H	16.060700000	7.564700000	4.090600000
H	15.840100000	5.831000000	3.839800000
H	16.392800000	6.853000000	2.506300000
C	-0.758500000	-1.779400000	1.488600000
C	-0.252600000	-1.251000000	2.693200000
C	-0.916300000	-0.979900000	0.340400000
C	0.033500000	0.128900000	2.775500000
C	-0.630800000	0.402400000	0.416500000
C	-0.198800000	0.926000000	1.643400000
C	-6.915900000	-3.768600000	3.917700000
C	-7.999700000	-3.223400000	3.192400000
C	-6.771900000	-3.433500000	5.278600000
C	-8.911700000	-2.369900000	3.815100000
C	-7.684500000	-2.578800000	5.906300000
C	-8.754300000	-2.049700000	5.172300000
H	-9.748300000	-1.949900000	3.261500000
H	-7.568100000	-2.329200000	6.955800000
Br	0.069600000	2.822300000	1.786900000
C	0.568500000	0.735400000	4.030600000
H	1.438600000	1.385300000	3.838000000
H	-0.193200000	1.352400000	4.529600000
H	0.914700000	-0.016800000	4.755700000
C	-0.029400000	-2.149500000	3.867500000
H	0.260200000	-3.168200000	3.573900000
H	0.776500000	-1.779800000	4.523700000
H	-0.939000000	-2.227800000	4.480400000
C	-0.779900000	1.292700000	-0.771900000
H	-1.489000000	2.115400000	-0.583200000

H	0.182500000	1.752700000	-1.049100000
H	-1.144100000	0.767500000	-1.666600000
C	-1.355400000	-1.599200000	-0.946200000
H	-1.911000000	-0.893200000	-1.584500000
H	-0.483300000	-1.944100000	-1.524700000
H	-2.019800000	-2.463200000	-0.805800000
Cu	-3.979400000	-2.531800000	2.514600000
C	-7.168300000	8.406900000	-3.477300000
C	-7.854700000	7.419100000	-3.342900000
C	-8.562100000	6.212200000	-3.126300000
C	-9.048200000	5.440700000	-4.193300000
C	-8.723900000	5.728700000	-1.814900000
C	-9.619300000	4.191400000	-3.951900000
H	-8.976400000	5.819000000	-5.213900000
C	-9.299500000	4.482000000	-1.591800000
H	-8.384100000	6.332500000	-0.972000000
C	-9.733500000	3.656300000	-2.653700000
H	-10.009400000	3.640800000	-4.809500000
H	-9.374000000	4.139400000	-0.560500000
C	-10.268400000	2.292200000	-2.394500000
C	-11.119300000	2.067600000	-1.300200000
C	-9.887700000	1.215200000	-3.249300000
N	-11.636300000	0.851100000	-0.870900000
C	-11.678900000	3.144300000	-0.407200000
N	-10.229900000	-0.102200000	-3.096000000
C	-8.991100000	1.333900000	-4.441600000
C	-12.467500000	1.114600000	0.266900000
Zn	-11.389600000	-0.984800000	-1.673900000
C	-12.468700000	2.578600000	0.518700000
H	-11.467300000	4.193500000	-0.537000000
C	-9.624800000	-0.836300000	-4.182700000
C	-8.855700000	0.118300000	-5.007000000
H	-8.546000000	2.255700000	-4.774600000
C	-13.151500000	0.224500000	1.023200000
N	-12.527300000	-1.837600000	-0.200600000
N	-11.099500000	-2.837400000	-2.478300000
H	-13.034900000	3.037900000	1.310600000
C	-9.703600000	-2.164100000	-4.429400000
H	-8.299800000	-0.168900000	-5.882200000
C	-13.173800000	-1.190900000	0.803000000
H	-13.745400000	0.582800000	1.870200000
C	-12.821400000	-3.229300000	-0.030800000
C	-10.404500000	-3.124800000	-3.632300000
C	-11.588300000	-4.072100000	-2.007300000
H	-9.189100000	-2.572900000	-5.308700000
C	-13.904600000	-2.131600000	1.654000000
C	-13.684400000	-3.377400000	1.140700000
C	-12.382100000	-4.252800000	-0.848400000
C	-10.445300000	-4.539000000	-3.917600000
C	-11.177500000	-5.124600000	-2.905100000
H	-14.492700000	-1.846100000	2.502900000
H	-14.059600000	-4.325800000	1.484400000
C	-12.769500000	-5.644500000	-0.500700000
H	-10.005100000	-5.010900000	-4.771200000
H	-11.419000000	-6.163700000	-2.780200000
C	-12.018400000	-6.370500000	0.444400000
C	-13.890600000	-6.239500000	-1.119400000
C	-12.379700000	-7.687000000	0.756600000
C	-10.844100000	-5.735600000	1.116600000
C	-14.241800000	-7.554200000	-0.792900000
C	-14.701000000	-5.467200000	-2.105600000
C	-13.490000000	-8.282500000	0.141200000
H	-11.797900000	-8.250200000	1.481100000
H	-10.114600000	-5.367600000	0.378700000
H	-11.158700000	-4.868700000	1.719500000
H	-10.315100000	-6.422300000	1.787900000
H	-15.107000000	-8.015000000	-1.265100000
H	-15.141000000	-4.568200000	-1.645800000
H	-14.083400000	-5.118100000	-2.949600000

H	-15.529000000	-6.048000000	-2.531900000
C	-13.879800000	-9.685700000	0.470700000
H	-13.816700000	-10.334600000	-0.415800000
H	-13.248400000	-10.137300000	1.246500000
H	-14.918200000	-9.738800000	0.831600000
C	-8.175300000	-3.563400000	1.739800000
H	-7.289100000	-3.260800000	1.170300000
H	-8.319000000	-4.640100000	1.608400000
H	-9.042900000	-3.053200000	1.306700000
C	-9.745000000	-1.138300000	5.836200000
H	-9.965200000	-0.272400000	5.203300000
H	-10.683800000	-1.672800000	6.018400000
H	-9.376300000	-0.766900000	6.797400000
C	-5.624900000	-3.998900000	6.073300000
H	-5.614800000	-5.092100000	6.014400000
H	-4.673600000	-3.616400000	5.667600000
H	-5.692200000	-3.715900000	7.128700000
C	6.771300000	-3.626600000	-1.484200000
C	7.665400000	-4.309100000	-1.923900000
C	8.703300000	-5.117000000	-2.408500000
C	8.539901620	-6.471385540	-2.650690860
C	9.976092490	-4.508128270	-2.689865320
C	9.626316330	-7.218105600	-3.170694200
H	7.588096340	-6.968530010	-2.448450840
C	10.857649930	-6.546335320	-3.427388620
C	9.500196060	-8.632049690	-3.440458620
C	11.982826990	-7.310610910	-3.966047900
C	10.544701850	-9.340183650	-3.940415140
H	8.541774210	-9.109796190	-3.230432100
C	11.810877830	-8.701467900	-4.216543470
H	10.455639240	-10.408866690	-4.146902210
C	12.909054170	-9.434755930	-4.739950460
C	14.197316220	-7.388990900	-4.712774750
C	14.098732160	-8.788618180	-4.988769440
H	12.797605510	-10.502717190	-4.938499420
H	14.962483130	-9.322694000	-5.388315250
C	15.467889000	-6.682343730	-4.973431450
C	16.312504210	-6.332689230	-3.900647170
C	15.832145910	-6.351980270	-6.294275490
C	17.516488950	-5.662919710	-4.154004390
C	17.038138140	-5.683525140	-6.535238260
C	17.879235370	-5.338130900	-5.467942520
H	18.169868600	-5.392016630	-3.327071910
H	17.322839390	-5.427396500	-7.553964530
C	10.185064710	-3.062068030	-2.431906270
C	10.697290180	-2.656891120	-1.187748670
C	9.871440700	-2.128170160	-3.432964870
C	10.898050270	-1.284519240	-0.931696930
C	10.070596240	-0.752073300	-3.186969480
C	10.579350980	-0.351543620	-1.937165880
C	9.337578060	-2.631533000	-4.745235950
H	8.957573550	-1.828442000	-5.381483980
H	10.136476300	-3.147566430	-5.291943950
H	8.520138510	-3.340758770	-4.581934110
C	11.019927020	-3.713462630	-0.167425410
H	10.165078320	-4.381497890	-0.023917170
H	11.869237940	-4.312961630	-0.518348150
H	11.283530980	-3.293714760	0.806211640
C	11.453993090	-0.862582320	0.401790750
H	11.440142780	0.220075990	0.551427830
H	10.877262450	-1.313995010	1.214417070
H	12.494641480	-1.194085060	0.487544650
C	9.738576170	0.237974550	-4.270069390
H	9.880871900	1.278104870	-3.965614630
H	10.377848150	0.062285310	-5.142214540
H	8.693603130	0.126946590	-4.576703640
C	15.927984820	-6.671609890	-2.490460770
H	15.096742700	-6.029253640	-2.173399270
H	15.602488260	-7.713052560	-2.412768710

H	16.760504020	-6.523415430	-1.797235640
C	14.933489070	-6.708834020	-7.442869090
H	14.797600300	-7.792589520	-7.504383850
H	13.949240390	-6.245811320	-7.309131330
H	15.342632990	-6.364095250	-8.397040510
C	19.169231070	-4.620762630	-5.740544840
H	19.899117260	-5.311572690	-6.174247010
H	19.014138280	-3.799220250	-6.446748030
H	19.598953390	-4.202297120	-4.825666080
N	13.176385450	-6.678627390	-4.219179640
N	11.004090220	-5.201959660	-3.178516050
Br	10.861501580	1.518541410	-1.596854400
C	-6.142000000	13.084600000	-4.833900000
H	-5.960800000	13.909400000	-4.136600000
H	-5.563900000	13.274700000	-5.744500000
H	-7.203700000	13.096100000	-5.099700000

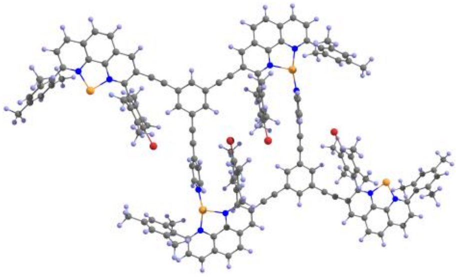

Figure S47. PM6-optimized structure of rotor $[Cu_4(\mathbf{A})_2]^{4+}$.

C	0.183623000	-8.352071000	0.126790000
C	0.605919000	-6.047938000	0.333939000
C	1.553467000	-8.656736000	-0.047224000
C	-0.785420000	-9.445760000	0.129145000
C	2.019214000	-6.279705000	0.138005000
C	2.474100000	-7.576896000	-0.048110000
C	1.973886000	-10.027376000	-0.211217000
C	-0.338421000	-10.776095000	-0.022930000
H	3.539997000	-7.777130000	-0.193916000
C	1.068796000	-11.042306000	-0.195916000
H	3.040453000	-10.225174000	-0.344875000
C	-1.316814000	-11.814573000	0.000426000
C	-3.035943000	-10.121566000	0.302230000
H	1.383702000	-12.083549000	-0.314991000
H	-0.998293000	-12.856164000	-0.110854000
C	-2.641629000	-11.491491000	0.162421000
H	-3.421048000	-12.259912000	0.187684000
N	-0.291048000	-7.054553000	0.310766000
N	-2.130783000	-9.115845000	0.285907000
C	2.949893000	-5.217774000	0.128611000
C	3.797365000	-4.354173000	0.102193000
C	4.800154000	-3.353493000	0.065353000
C	6.155844000	-3.726782000	-0.006984000
C	4.436756000	-1.997430000	0.097731000
C	7.146690000	-2.733715000	-0.048855000
H	6.434090000	-4.781542000	-0.030770000
C	5.438301000	-1.010545000	0.061562000
H	3.383991000	-1.713710000	0.147610000
C	6.791988000	-1.371095000	-0.014706000
C	0.123873000	-4.662899000	0.596394000
C	0.045437000	-4.215935000	1.931217000
C	-0.208174000	-3.819387000	-0.481848000
C	-0.436529000	-2.915800000	2.199456000
C	-0.680873000	-2.513961000	-0.220393000
C	-0.826079000	-2.114090000	1.116161000
C	-4.467346000	-9.796878000	0.464257000
C	-5.042173000	-9.765670000	1.755283000
C	-5.280556000	-9.601995000	-0.674966000

C	-6.409709000	-9.510936000	1.895120000
C	-6.645644000	-9.347818000	-0.513500000
C	-7.219489000	-9.305367000	0.767183000
H	-6.858060000	-9.496463000	2.887941000
H	-7.277410000	-9.208441000	-1.389457000
C	-0.054900000	-4.329287000	-1.877657000
H	-0.389841000	-3.604078000	-2.637599000
H	0.998694000	-4.556124000	-2.106233000
H	-0.631981000	-5.250637000	-2.040247000
C	-1.022911000	-1.569647000	-1.324049000
H	-0.434899000	-0.630285000	-1.242428000
H	-0.817930000	-1.986467000	-2.322615000
H	-2.087703000	-1.289740000	-1.307286000
C	0.472607000	-5.130463000	3.032343000
H	0.356146000	-4.680247000	4.033237000
H	-0.104680000	-6.065990000	3.037176000
H	1.536499000	-5.400499000	2.938794000
C	-0.536314000	-2.394184000	3.593727000
H	-0.141325000	-3.098775000	4.344431000
H	0.034644000	-1.458361000	3.714468000
H	-1.576497000	-2.177406000	3.879444000
Br	-1.642300000	-0.410710000	1.484573000
C	-6.791971000	1.371108000	-0.014706000
C	-7.146689000	2.733723000	-0.048827000
C	-5.438278000	1.010571000	0.061490000
C	-6.155851000	3.726801000	-0.006988000
C	-4.436739000	1.997466000	0.097627000
C	-4.800153000	3.353525000	0.065283000
H	-6.434108000	4.781559000	-0.030745000
H	-3.383970000	1.713752000	0.147458000
C	-3.797376000	4.354219000	0.102081000
C	-2.949897000	5.217815000	0.128470000
C	-2.019215000	6.279743000	0.137844000
C	-2.474068000	7.576927000	-0.048358000
C	-0.605927000	6.047966000	0.333849000
C	-1.553417000	8.656758000	-0.047477000
H	-3.539953000	7.777177000	-0.194233000
C	-0.183585000	8.352089000	0.126616000
C	-1.973819000	10.027389000	-0.211554000
C	0.785471000	9.445764000	0.128962000
C	-1.068718000	11.042311000	-0.196271000
H	-3.040380000	10.225196000	-0.345259000
C	0.338488000	10.776093000	-0.023217000
H	-1.383611000	12.083551000	-0.315411000
C	1.316885000	11.814570000	0.000099000
C	3.035990000	10.121576000	0.302084000
H	0.998371000	12.856154000	-0.111266000
C	2.641692000	11.491495000	0.162158000
H	3.421115000	12.259915000	0.187384000
N	0.291055000	7.054563000	0.310670000
N	2.130832000	9.115857000	0.285806000
C	-0.123910000	4.662930000	0.596379000
C	-0.045565000	4.216005000	1.931220000
C	0.208199000	3.819384000	-0.481817000
C	0.436389000	2.915881000	2.199530000
C	0.680864000	2.513959000	-0.220294000
C	0.825990000	2.114131000	1.116282000
C	4.467385000	9.796886000	0.464184000
C	5.280650000	9.601957000	-0.674989000
C	5.042131000	9.765737000	1.755246000
C	6.645730000	9.347808000	-0.513433000
C	6.409660000	9.511006000	1.895169000
C	7.219500000	9.305408000	0.767286000
H	7.277548000	9.208414000	-1.389347000
H	6.857954000	9.496561000	2.888015000
H	7.567229000	-0.600060000	-0.046757000
C	5.068947000	0.357240000	0.111360000
C	4.731059000	1.517286000	0.165677000
C	4.283717000	2.857202000	0.239973000
C	3.815226000	3.384222000	1.457641000
C	4.257223000	3.673983000	-0.905319000
C	3.304959000	4.685933000	1.482641000
H	3.830344000	2.776047000	2.364609000
C	3.734766000	4.966777000	-0.802475000
H	4.632292000	3.300179000	-1.859292000
H	2.915575000	5.107922000	2.421250000
H	3.701999000	5.619564000	-1.686901000
N	3.245378000	5.481586000	0.369390000
Cu	2.392980000	7.202074000	0.403624000
C	0.055019000	4.329239000	-1.877653000
H	0.390032000	3.604013000	-2.637548000
H	-0.998564000	4.556045000	-2.106313000
H	0.632093000	5.250595000	-2.040231000
C	1.022963000	1.569610000	-1.323901000
H	0.435010000	0.630213000	-1.242246000
H	0.817951000	1.986366000	-2.322487000
H	2.087773000	1.289774000	-1.307115000
C	0.536111000	2.394320000	3.593826000
H	-0.034893000	1.458528000	3.714588000
H	1.576275000	2.177510000	3.879583000
H	0.141127000	3.098960000	4.344488000
C	-0.472808000	5.130568000	3.032288000
H	-0.356590000	4.680311000	4.033193000
H	0.104617000	6.066010000	3.037246000
H	-1.536642000	5.400763000	2.938557000
C	4.701226000	9.695262000	-2.048764000
H	3.740610000	9.175582000	-2.145736000
H	4.531562000	10.749563000	-2.327826000
H	5.369562000	9.283091000	-2.819021000
C	4.208497000	10.030902000	2.965415000
H	3.983649000	11.107177000	3.057230000
H	3.249947000	9.497816000	2.950170000
H	4.715951000	9.747374000	3.899845000
C	8.681932000	9.070162000	0.927722000
H	9.227609000	10.030572000	0.951089000
H	8.930670000	8.549063000	1.862746000
H	9.115761000	8.485321000	0.105257000
Br	1.642191000	0.410763000	1.484797000
H	-7.567207000	0.600067000	-0.046721000
C	-5.068906000	-0.357211000	0.111256000
C	-4.731012000	-1.517255000	0.165549000
C	-4.283676000	-2.857175000	0.239817000
C	-3.815255000	-3.384251000	1.457491000
C	-4.257117000	-3.673904000	-0.905507000
C	-3.305007000	-4.685965000	1.482468000
H	-3.830419000	-2.776106000	2.364479000
C	-3.734675000	-4.966711000	-0.802687000
H	-4.632123000	-3.300063000	-1.859490000
H	-2.915681000	-5.107998000	2.421082000
H	-3.701849000	-5.619454000	-1.687143000
N	-3.245372000	-5.481575000	0.369181000
Cu	-2.393042000	-7.202082000	0.403589000
C	-8.522796000	3.064282000	-0.126385000
C	-9.714367000	3.266932000	-0.193804000
C	-11.119979000	3.380845000	-0.273344000
C	-11.780399000	4.592548000	-0.207947000
C	-11.886339000	2.155060000	-0.423026000
C	-13.202860000	4.623393000	-0.284176000
H	-11.224443000	5.528848000	-0.096881000
N	-13.228258000	2.159809000	-0.492642000
C	-11.124003000	0.880532000	-0.499928000
C	-13.884687000	3.394695000	-0.422847000
C	-13.955831000	5.851353000	-0.223217000
Cu	-14.623556000	0.764407000	-0.675826000
C	-10.674302000	0.430149000	-1.756884000
C	-10.751474000	0.235754000	0.696882000
C	-15.341764000	3.391015000	-0.499947000
C	-15.315875000	5.845289000	-0.296569000

H	-13.403367000	6.789049000	-0.116175000
N	-15.977310000	2.153524000	-0.635033000
C	-9.799377000	-0.677212000	-1.819559000
C	-11.093146000	1.159579000	-2.991371000
C	-9.869031000	-0.864762000	0.639901000
C	-11.259023000	0.758111000	2.001056000
C	-16.046455000	4.610507000	-0.437184000
H	-15.887398000	6.778263000	-0.250312000
C	-17.328597000	2.131919000	-0.709017000
C	-9.399370000	-1.278704000	-0.616654000
C	-9.291350000	-1.190359000	-3.124791000
H	-10.819196000	0.620259000	-3.916112000
H	-12.178386000	1.318339000	-3.038910000
H	-10.607556000	2.146391000	-3.054241000
C	-9.421562000	-1.564457000	1.879269000
H	-11.066324000	0.064498000	2.839008000
H	-10.768981000	1.708286000	2.267127000
H	-12.342695000	0.933947000	1.990887000
C	-17.472550000	4.556809000	-0.517596000
C	-18.096843000	3.342242000	-0.650716000
C	-18.017466000	0.837478000	-0.852110000
Br	-8.125211000	-2.712221000	-0.688548000
H	-8.199697000	-1.075999000	-3.210043000
H	-9.520107000	-2.260632000	-3.258583000
H	-9.731718000	-0.670350000	-3.991975000
H	-8.336187000	-1.463135000	2.033885000
H	-9.908761000	-1.178747000	2.790102000
H	-9.647641000	-2.642930000	1.841992000
H	-18.048483000	5.487497000	-0.471806000
H	-19.188032000	3.260744000	-0.715728000
C	-18.456686000	0.139320000	0.297181000
C	-18.324203000	0.338225000	-2.139770000
C	-19.159654000	-1.058531000	0.149593000
C	-18.203844000	0.685796000	1.663882000
C	-19.028394000	-0.861442000	-2.264340000
C	-17.930552000	1.096278000	-3.364845000
C	-19.453680000	-1.567191000	-1.126462000
H	-19.505487000	-1.596391000	1.032572000
H	-17.204984000	1.120100000	1.782033000
H	-18.937426000	1.473095000	1.909018000
H	-18.311632000	-0.079167000	2.449048000
H	-19.272406000	-1.246516000	-3.254690000
H	-18.640449000	1.919186000	-3.557029000
H	-16.928666000	1.535228000	-3.303042000
H	-17.946415000	0.469264000	-4.270349000
C	-20.229177000	-2.828574000	-1.272143000
H	-21.312160000	-2.617456000	-1.331791000
H	-19.972806000	-3.388724000	-2.183308000
H	-20.094761000	-3.517920000	-0.425825000
C	8.522793000	-3.064278000	-0.126466000
C	9.714359000	-3.266942000	-0.193924000
C	11.119968000	-3.380870000	-0.273514000
C	11.780374000	-4.592587000	-0.208241000
C	11.886340000	-2.155081000	-0.423111000
C	13.202831000	-4.623442000	-0.284502000
H	11.224407000	-5.528890000	-0.097249000
N	13.228259000	-2.159839000	-0.492743000
C	11.124014000	-0.880540000	-0.499914000
C	13.884672000	-3.394738000	-0.423067000
C	13.955788000	-5.851418000	-0.223681000
Cu	14.623576000	-0.764436000	-0.675771000
C	10.674319000	-0.430055000	-1.756835000
C	10.751494000	-0.235848000	0.696944000

C	15.341749000	-3.391072000	-0.500186000
C	15.315829000	-5.845366000	-0.297061000
H	13.403312000	-6.789117000	-0.116719000
N	15.977310000	-2.153575000	-0.635150000
C	9.799395000	0.677313000	-1.819425000
C	11.093161000	-1.159386000	-2.991381000
C	9.869055000	0.864673000	0.640050000
C	11.259051000	-0.758301000	2.001077000
C	16.046424000	-4.610580000	-0.437565000
H	15.887341000	-6.778353000	-0.250911000
C	17.328596000	-2.131982000	-0.709162000
C	9.399389000	1.278711000	-0.616473000
C	9.291368000	1.190560000	-3.124617000
H	10.819187000	-0.620006000	-3.916079000
H	12.178405000	-1.318117000	-3.038946000
H	10.607595000	-2.146206000	-3.054316000
C	9.421592000	1.564279000	1.879472000
H	11.066413000	-0.064720000	2.839070000
H	10.768961000	-1.708463000	2.267111000
H	12.342711000	-0.934199000	1.990870000
C	17.472518000	-4.556895000	-0.518002000
C	18.096826000	-3.342323000	-0.651007000
C	18.017482000	-0.837537000	-0.852132000
Br	8.125222000	2.712227000	-0.688251000
H	8.199715000	1.076208000	-3.209878000
H	9.520128000	2.260842000	-3.258329000
H	9.731734000	0.670615000	-3.991841000
H	8.336219000	1.462939000	2.034089000
H	9.908800000	1.178505000	2.790273000
H	9.647666000	2.642755000	1.842269000
H	18.048438000	-5.487596000	-0.472321000
H	19.188014000	-3.260834000	-0.716037000
C	18.456758000	-0.139522000	0.297235000
C	18.324183000	-0.338138000	-2.139735000
C	19.159741000	1.058323000	0.149764000
C	18.203942000	-0.686162000	1.663875000
C	19.028395000	0.861540000	-2.264189000
C	17.930484000	-1.096031000	-3.364893000
C	19.453733000	1.567137000	-1.126251000
H	19.505630000	1.596079000	1.032786000
H	17.205050000	-1.120393000	1.782016000
H	18.937467000	-1.473557000	1.908868000
H	18.311837000	0.078686000	2.449140000
H	19.272367000	1.246718000	-3.254508000
H	18.640414000	-1.918872000	-3.557246000
H	16.928628000	-1.535048000	-3.303087000
H	17.946251000	-0.468883000	-4.270306000
C	20.229261000	2.828530000	-1.271682000
H	21.312529000	2.617708000	-1.327064000
H	19.975953000	3.386899000	-2.184786000
H	20.091557000	3.519388000	-0.427119000
C	-4.208644000	-10.030825000	2.965531000
H	-3.984612000	-11.107238000	3.057848000
H	-3.249697000	-9.498477000	2.949986000
H	-4.715839000	-9.746463000	3.899846000
C	-4.701056000	-9.695391000	-2.048705000
H	-4.531219000	-10.749702000	-2.327622000
H	-5.369425000	-9.283432000	-2.819058000
H	-3.740530000	-9.175553000	-2.145694000
C	-8.681928000	-9.070113000	0.927535000
H	-8.930695000	-8.548871000	1.862473000
H	-9.115748000	-8.485403000	0.104968000
H	-9.227590000	-10.030530000	0.951033000

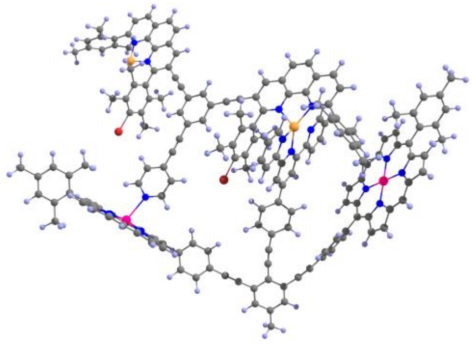

Figure S48. PM6-optimized structure of rotor $[Cu_2(\mathbf{A})(\mathbf{B})]^{2+}$.

C	-4.032500000	10.403400000	-3.152400000
C	-4.433500000	11.621300000	-3.726300000
C	-6.651700000	10.716900000	-4.147400000
C	-6.268700000	9.498900000	-3.574500000
C	-4.950700000	9.330300000	-3.066600000
H	-3.720300000	12.444100000	-3.786100000
H	-7.664200000	10.836100000	-4.532500000
C	-4.594100000	8.084600000	-2.503700000
C	-4.375200000	6.981700000	-2.054200000
C	-5.734300000	11.777400000	-4.221600000
C	-4.282700000	5.691700000	-1.484400000
C	-5.340000000	4.780000000	-1.691500000
C	-3.163400000	5.300400000	-0.726800000
C	-5.282000000	3.506500000	-1.133500000
H	-6.204900000	5.082100000	-2.288500000
C	-3.114100000	4.025300000	-0.164900000
H	-2.340700000	6.000900000	-0.578600000
C	-4.171200000	3.122200000	-0.360400000
H	-6.109000000	2.812500000	-1.289500000
H	-2.241700000	3.727600000	0.419800000
C	-4.145800000	1.779700000	0.251100000
C	-3.812900000	1.607800000	1.606300000
C	-4.488800000	0.647000000	-0.497200000
C	-3.856900000	0.322100000	2.161700000
H	-3.536600000	2.473400000	2.212000000
C	-4.509400000	-0.622500000	0.116800000
H	-4.730700000	0.748000000	-1.557800000
C	-4.900700000	-1.827000000	-0.677600000
C	-5.203900000	-1.734300000	-2.049100000
C	5.917200000	-1.471800000	-0.756200000
H	6.872300000	-1.004500000	-1.002100000
C	5.039300000	0.692700000	-0.019100000
C	5.193400000	1.877000000	0.175100000
C	5.436100000	3.255700000	0.396300000
C	6.744600000	3.758400000	0.312400000
C	4.390600000	4.145700000	0.700200000
C	6.963400000	5.122900000	0.536600000
H	7.580400000	3.099300000	0.077700000
C	4.690800000	5.500200000	0.908200000
H	3.363600000	3.791200000	0.774000000
H	7.991600000	5.516800000	0.472300000
H	3.889100000	6.218200000	1.143400000
N	5.958800000	5.998800000	0.833200000
C	-2.714700000	10.249900000	-2.656400000
C	-1.586600000	10.098800000	-2.243800000
C	-0.263400000	9.892600000	-1.782400000
C	0.425400000	10.897300000	-1.076700000
C	0.366600000	8.658300000	-2.033900000
C	1.727700000	10.668700000	-0.632900000
H	-0.058100000	11.855500000	-0.888000000
C	1.665200000	8.434200000	-1.583300000
H	-0.164800000	7.883800000	-2.585800000
C	2.357500000	9.436000000	-0.879100000
H	2.269800000	11.450200000	-0.099700000
H	2.157200000	7.483400000	-1.781900000
C	-5.544500000	-2.896400000	-2.750100000
H	-5.178100000	-0.774300000	-2.567200000
C	-5.253500000	-4.138000000	-0.709800000
C	-5.573200000	-4.118300000	-2.079100000
H	-5.782800000	-2.841900000	-3.814800000
H	-5.260100000	-5.076700000	-0.141200000
H	-5.839900000	-5.038100000	-2.598300000
C	-3.562700000	0.087100000	3.604600000
C	-3.214500000	1.129900000	4.480700000
C	-3.465400000	-1.476400000	5.347300000
C	-2.989600000	0.835500000	5.829400000
H	-3.119300000	2.155100000	4.117600000
C	-3.122500000	-0.480500000	6.275800000
H	-3.598300000	-2.533100000	5.661800000
H	-2.717200000	1.631800000	6.526000000
H	-2.963000000	-0.734300000	7.323600000
N	-4.198100000	-0.797700000	1.435400000
N	-3.672500000	-1.218400000	4.026900000
N	-4.920200000	-3.023400000	-0.012300000
C	-2.670600000	-4.943500000	1.758100000
C	-1.112100000	-3.223400000	1.419900000
C	-1.783000000	-5.926900000	1.260200000
C	-3.989000000	-5.346200000	2.250700000
C	-0.141000000	-4.171100000	0.919100000
C	-0.492300000	-5.509300000	0.835000000
C	-2.201100000	-7.307600000	1.204800000
C	-4.350900000	-6.712000000	2.194000000
H	0.215900000	-6.246800000	0.447600000
C	-3.430900000	-7.681300000	1.648300000
H	-1.498800000	-8.038700000	0.798300000
C	-5.628200000	-7.082400000	2.703300000
C	-6.011800000	-4.751300000	3.285100000
H	-3.752600000	-8.724500000	1.608800000
H	-5.939000000	-8.129000000	2.663000000
C	-6.443900000	-6.119100000	3.244500000
H	-7.425600000	-6.366500000	3.653800000
N	-2.340500000	-3.594200000	1.819900000
N	-4.820700000	-4.358500000	2.780000000
C	1.133000000	-3.710200000	0.526600000
C	2.214000000	-3.261600000	0.218500000
C	3.459700000	-2.676800000	-0.118700000
C	4.501500000	-3.458800000	-0.645600000
C	3.635900000	-1.297100000	0.087100000
C	5.725800000	-2.847600000	-0.959900000
H	4.362700000	-4.529600000	-0.804100000
C	4.866900000	-0.698500000	-0.229800000
H	2.822900000	-0.695100000	0.49500000

C	3.735100000	9.173800000	-0.416500000
C	4.755500000	9.098100000	-1.390900000
C	3.989900000	9.038400000	0.951600000
N	6.063500000	8.785500000	-1.176300000
C	4.536600000	9.385200000	-2.844800000
N	5.192000000	8.716800000	1.532000000
C	2.954700000	9.222000000	2.023500000
Zn	6.961600000	8.221400000	0.586200000
C	6.740400000	8.887500000	-2.445500000
C	5.725000000	9.256500000	-3.470800000
H	3.576300000	9.651400000	-3.247800000
C	4.991900000	8.709300000	2.951200000
C	3.556000000	9.028300000	3.213800000
H	1.932100000	9.471800000	1.805500000
N	8.804400000	8.235100000	-0.334500000
N	7.899800000	8.114700000	2.418500000
C	8.060800000	8.704500000	-2.671600000
H	5.965600000	9.389100000	-4.509400000
C	5.924300000	8.461100000	3.899700000
H	3.144800000	9.084900000	4.204200000
C	9.049300000	8.392300000	-1.671800000
C	10.051400000	7.959400000	0.273200000
C	7.315600000	8.186800000	3.644200000
C	9.285700000	7.863500000	2.641200000
H	8.448000000	8.805400000	-3.692100000
H	5.636700000	8.475600000	4.954900000

C	10.460800000	8.215200000	-1.953800000
C	11.075300000	7.946400000	-0.751300000
C	10.265700000	7.792000000	1.655600000
C	8.302000000	7.967400000	4.699100000
C	9.506700000	7.766200000	4.079400000
H	10.906600000	8.291700000	-2.923200000
H	12.116600000	7.762100000	-0.564100000
C	11.663700000	7.534300000	2.095900000
H	8.081600000	7.974200000	5.746100000
H	10.466100000	7.575100000	4.524800000
C	12.134500000	6.213400000	2.232300000
C	12.519200000	8.620700000	2.377400000
C	13.450800000	5.984300000	2.651200000
C	11.231400000	5.062300000	1.927700000
C	13.832800000	8.376000000	2.795800000
C	12.022500000	10.019700000	2.229300000
C	14.301800000	7.062100000	2.935100000
H	13.815400000	4.966100000	2.755300000
H	10.320800000	5.101600000	2.546200000
H	10.911700000	5.084800000	0.875100000
H	11.705700000	4.089700000	2.106100000
H	14.493800000	9.211700000	3.012300000
H	11.682700000	10.214600000	1.198100000
H	11.154900000	10.207400000	2.882400000
H	12.780100000	10.775200000	2.469500000
C	15.709900000	6.814100000	3.368900000
H	16.060700000	7.564700000	4.090600000
H	15.840100000	5.831000000	3.839800000
H	16.392800000	6.853000000	2.506300000
C	-0.758500000	-1.779400000	1.488600000
C	-0.252600000	-1.251000000	2.693200000
C	-0.916300000	-0.979900000	0.340400000
C	0.033500000	0.128900000	2.775500000
C	-0.630800000	0.402400000	0.416500000
C	-0.198800000	0.926000000	1.643400000
C	-6.915900000	-3.768600000	3.917700000
C	-7.999700000	-3.223400000	3.192400000
C	-6.771900000	-3.433500000	5.278600000
C	-8.911700000	-2.369900000	3.815100000
C	-7.684500000	-2.578800000	5.906300000
C	-8.754300000	-2.049700000	5.172300000
H	-9.748300000	-1.949900000	3.261500000
H	-7.568100000	-2.329200000	6.955800000
Br	0.069600000	2.822300000	1.786900000
C	0.568500000	0.735400000	4.030600000
H	1.438600000	1.385300000	3.838000000
H	-0.193200000	1.352400000	4.529600000
H	0.914700000	-0.016800000	4.755700000
C	-0.029400000	-2.149500000	3.867500000
H	0.260200000	-3.168200000	3.573900000
H	0.776500000	-1.779800000	4.523700000
H	-0.939000000	-2.227800000	4.480400000
C	-0.779900000	1.292700000	-0.771900000
H	-1.489000000	2.115400000	-0.583200000
H	0.182500000	1.752700000	-1.049100000
H	-1.144100000	0.767500000	-1.666600000
C	-1.355400000	-1.599200000	-0.946200000
H	-1.911000000	-0.893200000	-1.584500000
H	-0.483300000	-1.944100000	-1.524700000
H	-2.019800000	-2.463200000	-0.805800000
Cu	-3.979400000	-2.531800000	2.514600000
C	-7.168300000	8.406900000	-3.477300000
C	-7.854700000	7.419100000	-3.342900000
C	-8.562100000	6.212200000	-3.126300000
C	-9.048200000	5.440700000	-4.193300000
C	-8.723900000	5.728700000	-1.814900000
C	-9.619300000	4.191400000	-3.951900000
H	-8.976400000	5.819000000	-5.213900000
C	-9.299500000	4.482000000	-1.591800000
H	-8.384100000	6.332500000	-0.972000000
C	-9.733500000	3.656300000	-2.653700000
H	-10.009400000	3.640800000	-4.809500000
H	-9.374000000	4.139400000	-0.560500000
C	-10.268400000	2.292200000	-2.394500000
C	-11.119300000	2.067600000	-1.300200000
C	-9.887700000	1.215200000	-3.249300000
N	-11.636300000	0.851100000	-0.870900000
C	-11.678900000	3.144300000	-0.407200000
N	-10.229900000	-0.102200000	-3.096000000
C	-8.991100000	1.333900000	-4.441600000
C	-12.467500000	1.114600000	0.266900000
Zn	-11.389600000	-0.984800000	-1.673900000
C	-12.468700000	2.578600000	0.518700000
H	-11.467300000	4.193500000	-0.537000000
C	-9.624800000	-0.836300000	-4.182700000
C	-8.855700000	0.118300000	-5.007000000
H	-8.546000000	2.255700000	-4.774600000
C	-13.151500000	0.224500000	1.023200000
N	-12.527300000	-1.837600000	-0.200600000
N	-11.099500000	-2.837400000	-2.478300000
H	-13.034900000	3.037900000	1.310600000
C	-9.703600000	-2.164100000	-4.429400000
H	-8.299800000	-0.168900000	-5.882200000
C	-13.173800000	-1.190900000	0.803000000
H	-13.745400000	0.582800000	1.870200000
C	-12.821400000	-3.229300000	-0.030800000
C	-10.404500000	-3.124800000	-3.632300000
C	-11.588300000	-4.072100000	-2.007300000
H	-9.189100000	-2.572900000	-5.308700000
C	-13.904600000	-2.131600000	1.654000000
C	-13.684400000	-3.377400000	1.140700000
C	-12.382100000	-4.252800000	-0.848400000
C	-10.445300000	-4.539000000	-3.917600000
C	-11.177500000	-5.124600000	-2.905100000
H	-14.492700000	-1.846100000	2.502900000
H	-14.059600000	-4.325800000	1.484400000
C	-12.769500000	-5.644500000	-0.500700000
H	-10.005100000	-5.010900000	-4.771200000
H	-11.419000000	-6.163700000	-2.780200000
C	-12.018400000	-6.370500000	0.444400000
C	-13.890600000	-6.239500000	-1.119400000
C	-12.379700000	-7.687000000	0.756600000
C	-10.844100000	-5.735600000	1.116600000
C	-14.241800000	-7.554200000	-0.792900000
C	-14.701000000	-5.467200000	-2.105600000
C	-13.490000000	-8.282500000	0.141200000
H	-11.797900000	-8.250200000	1.481100000
H	-10.114600000	-5.367600000	0.378700000
H	-11.158700000	-4.868700000	1.719500000
H	-10.315100000	-6.422300000	1.787900000
H	-15.107000000	-8.015000000	-1.265100000
H	-15.141000000	-4.568200000	-1.645800000
H	-14.083400000	-5.118100000	-2.949600000
H	-15.529000000	-6.048000000	-2.531900000
C	-13.879800000	-9.685700000	0.470700000
H	-13.816700000	-10.334600000	-0.415800000
H	-13.248400000	-10.137300000	1.246500000
H	-14.918200000	-9.738800000	0.831600000
C	-8.175300000	-3.563400000	1.739800000
H	-7.289100000	-3.260800000	1.170300000
H	-8.319000000	-4.640100000	1.608400000
H	-9.042900000	-3.053200000	1.306700000
C	-9.745000000	-1.138300000	5.836200000
H	-9.965200000	-0.272400000	5.203300000
H	-10.683800000	-1.672800000	6.018400000
H	-9.376300000	-0.766900000	6.797400000
C	-5.624900000	-3.998900000	6.073300000
H	-5.614800000	-5.092100000	6.014400000
H	-4.673600000	-3.616400000	5.667600000
H	-5.692200000	-3.715900000	7.128700000
C	6.771300000	-3.626600000	-1.484200000
C	7.665400000	-4.309100000	-1.923900000
C	8.703300000	-5.117000000	-2.408500000
C	8.539901620	-6.471385540	-2.650690860
C	9.976092490	-4.508128270	-2.689865320
C	9.626316330	-7.218105600	-3.170694200
H	7.588096340	-6.968530010	-2.448450840
C	10.857649930	-6.546335320	-3.427388620
C	9.500196060	-8.632049690	-3.440458620
C	11.982826990	-7.310610910	-3.966047900

C	10.544701850	-9.340183650	-3.940415140
H	8.541774210	-9.109796190	-3.230432100
C	11.810877830	-8.701467900	-4.216543470
H	10.455639240	-10.408866690	-4.146902210
C	12.909054170	-9.434755930	-4.739950460
C	14.197316220	-7.388990900	-4.712774750
C	14.098732160	-8.788618180	-4.988769440
H	12.797605510	-10.502717190	-4.938499420
H	14.962483130	-9.322694000	-5.388315250
C	15.467889000	-6.682343730	-4.973431450
C	16.312504210	-6.332689230	-3.900647170
C	15.832145910	-6.351980270	-6.294275490
C	17.516488950	-5.662919710	-4.154004390
C	17.038138140	-5.683525140	-6.535238260
C	17.879235370	-5.338130900	-5.467942520
H	18.169868600	-5.392016630	-3.327071910
H	17.322839390	-5.427396500	-7.553964530
C	10.185064710	-3.062068030	-2.431906270
C	10.697290180	-2.656891120	-1.187748670
C	9.871440700	-2.128170160	-3.432964870
C	10.898050270	-1.284519240	-0.931696930
C	10.070596240	-0.752073300	-3.186969480
C	10.579350980	-0.351543620	-1.937165880
C	9.337578060	-2.631533000	-4.745235950
H	8.957573550	-1.828442000	-5.381483980
H	10.136476300	-3.147566430	-5.291943950
H	8.520138510	-3.340758770	-4.581934110
C	11.019927020	-3.713462630	-0.167425410
H	10.165078320	-4.381497890	-0.023917170
H	11.869237940	-4.312961630	-0.518348150
H	11.283530980	-3.293714760	0.806211640

C	11.453993090	-0.862582320	0.401790750
H	11.440142780	0.220075990	0.551427830
H	10.877262450	-1.313995010	1.214417070
H	12.494641480	-1.194085060	0.487544650
C	9.738576170	0.237974550	-4.270069390
H	9.880871900	1.278104870	-3.965614630
H	10.377848150	0.062285310	-5.142214540
H	8.693603130	0.126946590	-4.576703640
C	15.927984820	-6.671609890	-2.490460770
H	15.096742700	-6.029253640	-2.173399270
H	15.602488260	-7.713052560	-2.412768710
H	16.760504020	-6.523415430	-1.797235640
C	14.933489070	-6.708834020	-7.442869090
H	14.797600300	-7.792589520	-7.504383850
H	13.949240390	-6.245811320	-7.309131330
H	15.342632990	-6.364095250	-8.397040510
C	19.169231070	-4.620762630	-5.740544840
H	19.899117260	-5.311572690	-6.174247010
H	19.014138280	-3.799220250	-6.446748030
H	19.598953390	-4.202297120	-4.825666080
N	13.176385450	-6.678627390	-4.219179640
N	11.004090220	-5.201959660	-3.178516050
Br	10.861501580	1.518541410	-1.596854400
C	-6.142000000	13.084600000	-4.833900000
H	-5.960800000	13.909400000	-4.136600000
H	-5.563900000	13.274700000	-5.744500000
H	-7.203700000	13.096100000	-5.099700000
Cu	12.762214220	-4.902155220	-3.687570000

11. References

[1] A. Goswami, I. Paul, M. Schmittel, *Chem. Commun.* **2017**, *53*, 5186−5189.

[2] M. Schmittel, C. Michel, A. Wiegrefe, V. Kalsani, *Synthesis* **2001**, 1561 – 1567.
